# Development of ArgTag for Scalable Solid-Phase Synthesis of Aggregating Peptides

**DOI:** 10.1021/acschembio.5c00662

**Published:** 2025-10-23

**Authors:** Vincent Freiburghaus, Aliénor Jeandin, Łukasz Frankiewicz, Jie Yang, Nina Hartrampf

**Affiliations:** † Department of Chemistry, 27217 University of Zurich, Winterthurerstrasse 190, 8057 Zurich, Switzerland; ‡ PeptiSystems AB, Uppsala Business Park, Virdings allé 22, 754 50 Uppsala, Sweden

## Abstract

Aggregation during solid-phase peptide synthesis (SPPS) remains a key limitation, often leading to low coupling efficiencies and poor crude purities. Our previously introduced synthesis tag (“SynTag”) for chemical protein synthesis combines six C-terminal Arg­(Pbf) residues with a MeDbz linker to suppress aggregation via helical structure induction and serves as a handle for native chemical ligation (NCL). To apply the concept to short, yet aggregation-prone sequences, some practical limitations need to be addressed: Tag removal needs to be simplified, its utility demonstrated on more commonly used resin types and loadings, and the method must be effective on larger scale. To this end, we developed a simplified C-terminal hexaarginine tag (“ArgTag”) and refined an enzymatic method for efficient removal with Carboxypeptidase B, enabling selective cleavage under mild, linker-free conditions. We evaluated the ArgTag across six solid supports (resins) of varying polarity and loading. Using automated fast-flow SPPS (AFPS), we observed consistent aggregation suppression and improved crude purities across all resin types. We finally demonstrated the efficiency of our ArgTag on larger scale using more economical synthesis parameters. This work broadens the applicability of the SynTag strategy to short, yet difficult peptide sequences and offers a more scalable solution to improve SPPS efficiency for challenging targets.

## Graphical abstract

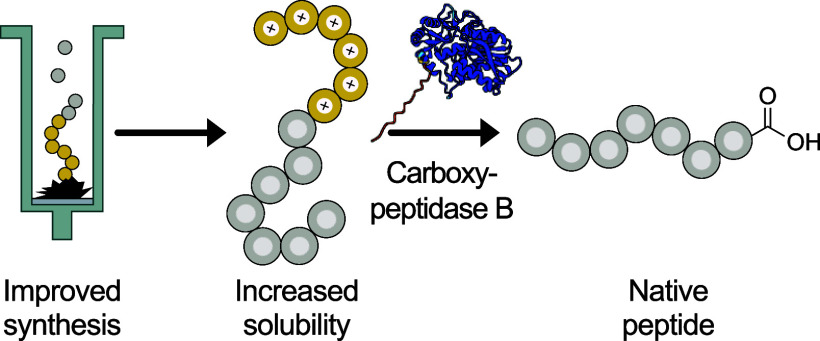

## Introduction

Chemical peptide synthesis provides access to natural, chemically modified and *de novo* peptides and proteins for applications in medicinal chemistry, chemical biology, synthetic biology or material science.^1^ Solid-phase peptide synthesis (SPPS) is still the method of choice for the stepwise chemical assembly of peptides, and allows for the site-selective incorporation of noncanonical amino acids, post-translational modifications (PTMs), and functional handles.^2^ Flow-SPPS is becoming increasingly popular, in part due to its ability to detect aggregation-prone sequences through real-time monitoring,^3,4^ thereby facilitating the identification of difficult peptides.^5,6^ In spite of these considerable advances, two major sequence-specific challenges remain: (i) aggregation of growing peptide chains during synthesis, and (ii) poor solubility of the cleaved peptides.^7,8^

Peptide aggregation during SPPS is driven by β-sheet formation between elongating, fully side-chain protected peptide chains.^9^ These interactions can cause the resin to collapse, ultimately reducing coupling efficiency and increasing side product formation (Figure 1A).^10–12^ The amino acid sequence, but also the resin type and linker, have all shown to influence aggregation propensity and crude peptide purity.^13–16^ Polystyrene (PS)-based resins, though inexpensive and high-loading, swell poorly and often show reduced performance with difficult sequences.^17^ PEG-based and hybrid PEG–PS resins show better swelling properties, have a higher polarity and can enhance the synthesis of aggregation-prone peptides. However, these resins are often more expensive than PS-based supports and typically offer lower loading capacities. In addition, the availability of pure PEG resins is limited, as key commercial products such as ChemMatrix and NovaPEG have been discontinued or are subject to supply constraints.^15,17,18^ Alternative polar resins such as the polyacrylamide-based Li-resin represent promising replacements, combining polar properties with high loadings (Figure 1B).^19^ Overall, the choice of resin remains a multifactorial decision involving trade-offs in cost, swelling, loading, and synthesis performance.

**Figure 1:**
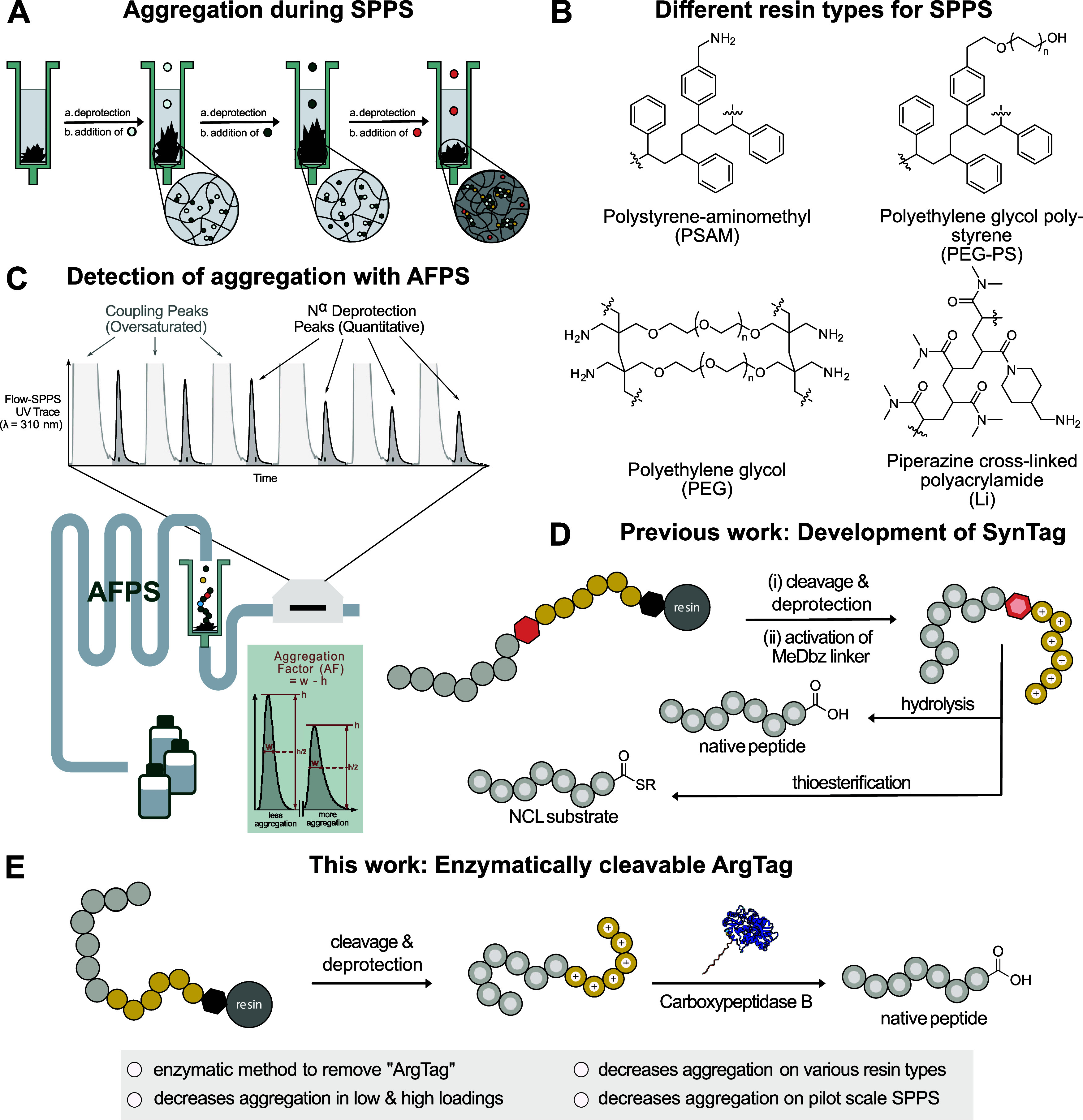
Flow-based solid-phase peptide synthesis enabled the development of an enzymatically cleavable “Arginine Tag” (ArgTag), that improves peptide crude purities across various resins, resin loadings and scales. (A) Iterative coupling and deprotection steps in SPPS. Left to right: the first two additions illustrate efficient coupling and deprotection reactions. Upon the third addition, the resin aggregates, leading to inefficient synthesis. (B) Different categories of solid supports investigated in this study ranging from apolar pure PS polymers or PEG–PS copolymers (top) and the most polar resins consisting either of pure PEG or piperazine cross-linked polyacrylamide (bottom). (C) AFPS technology with in-line, time-resolved UV–vis analysis enables the detection of aggregation onset during synthesis. The aggregation factor (AF) was defined as the difference between peak width at half-maximum (w) and peak height (h), providing a simple descriptor of peak broadening and flattening. The schematic values shown are for illustrative purposes only; all reported AF values in the manuscript were calculated directly from experimental UV traces. Lower AF values indicate efficient deprotection and a nonaggregated resin, whereas higher values reflect aggregation. (D) Previous work: Development of SynTag as a synthetic handle consisting of six Arg­(Pbf) residues and a MeDbz linker which upon activation can be hydrolyzed or converted into thioester for NCL. (E) This work: Investigation of the versatility of the ArgTag on various resin types and loadings, including an enzymatic tag cleavage, eliminating the need for the MeDbz linker as cleavage handle.

Flow-SPPS platforms, such as the automated fast-flow peptide synthesizer (AFPS), enable time-resolved, in-line UV monitoring of aggregation via Fmoc deprotection steps, facilitating method development (Figure 1C).^4,6,20,21^ We previously introduced the SynTag strategy, which uses six C-terminal Arg­(Pbf) residues in combination with a cleavable MeDbz linker (Figure 1D). It mitigates aggregation by promoting the formation of a helical structure in the growing peptide chain, resulting in improved synthesis outcomes.^9^ The presence of the MeDbz linker allows for either a NCL step or post-synthesis removal of the SynTag, but concurrently requires additional removal steps and can reduce crude purity in certain cases: When aggregation re-emerges in later synthesis stages, β-sheet formation can restrict chain flexibility, hindering MeDbz linker cyclization and therefore tag removal, even for short peptides such as Barstar[75–90].^9^ Furthermore, although the SynTag has proven effective on the comparatively low-loading and high-cost NovaPEG-RAM resin, its broader applicability across resin types and synthesis conditions has not yet been explored. Finally, the original approach was evaluated at small scale and comparatively low-loading resins, making it less attractive for the synthesis of short peptides.^9,22^

Here, we developed a simplified C-terminal hexaarginine tag (“ArgTag”) for the synthesis of difficult sequences with a native C-terminal carboxylic acid, and demonstrated its enzymatic removal, and applicability to a wide variety of resins, loadings and scales. To this end, we first established a mild enzymatic method for ArgTag removal using recombinant Carboxypeptidase B (CPB), enabling selective cleavage of the six C-terminal arginines under linker-free conditions. Building on this strategy, we then expanded the scope of the ArgTag to six different solid supports spanning a range of polarities and loadings. Across all resins, we observed consistent suppression of aggregation and improved crude purities, both at low and high loading. Finally, we validated the approach under pilot-scale conditions using more economical synthesis parameters, demonstrating the application of ArgTag to efficient, scalable SPPS of aggregation-prone peptides (Figure 1E).

## Results and Discussion

### Carboxypeptidase B Selectively Removes ArgTag and Yields the Native C-Terminal Carboxylic Acid

To establish a protocol for removing the six C-terminal Arg residues without relying on the MeDbz linker, we first evaluated a recently reported chemical method that mimics CPB activity.^23^ Using 9,10-phenanthrenequinone under basic conditions, selective removal of C-terminal Arg residues was achieved for two model peptides (SI Figures 2–6). However, peptides containing oxidation-sensitive residues such as Cys or Met, or internal Arg residues, presented challenges under these conditions, and the native sequence could not be consistently obtained (SI Figures 7–9).

We therefore turned to an enzymatic approach using CPB, which cleaves basic residues (Arg, Lys) from the C-terminus under mild conditions.^24^ Initial experiments using porcine-derived enzyme and Barstar[75–90]-ArgTag as a substrate showed promising activity (SI Figures 10 and 11). However, when applied to GLP-1[7–37]-ArgTag, nonspecific peptide degradation was observed. This issue was resolved by switching to high-purity recombinant CPB and subsequent reaction optimization: Initially, a low enzyme-to-peptide molar ratio of 1:50 and an enzyme concentration of 8 U/mL were chosen, which resulted in incomplete ArgTag removal (Figure 2A, **entry 1**). Next, the enzyme-to-peptide molar ratio was adjusted to 1:5 while keeping the enzyme concentration at 8 U/mL, resulting in complete ArgTag removal within 40 h (**entry 2**). To accelerate ArgTag removal, enzyme concentration was drastically increased to 229 U/mL while keeping the ratio to the peptide at 1:5. However, while initially increasing the rate of product formation, complete conversion to the desired GLP-1[7–37] was not achieved as the peptide was degraded (overdigested) (**entry 3**). Under the final conditions (pH 7.8, 37 °C, 40 h, 8 U/mL CPB, peptide substrate concentration 4.6 μM), ArgTag removal from GLP-1[7–37]-ArgTag was achieved without unspecific amide bond cleavage and the desired native GLP-1[7–37] could be isolated by standard reverse-phase HPLC (Figure 2B, for detailed conditions see SI Section 3.2.2). Interestingly, no intermediate species (e.g., GLP-Arg_5_, GLP-Arg_4_, GLP-Arg_3_) were detected during ArgTag removal, and only starting material and fully detagged product were observed. This likely reflects either rapid sequential hydrolysis of arginine residues once the peptide is bound to CPB, or tight substrate–enzyme association until no further C-terminal basic residues remain.^25,26^ Applying the optimized ArgTag removal conditions to MYC[123–143]-ArgTag (contains one Met and one Cys residue), and Barstar[75–90]-ArgTag (contains one Cys residue) demonstrated that the method is generally effective. For MYC[123–143] intermolecular disulfide formation was observed and led to slower and therefore incomplete conversion. The effects caused by the disulfide bond formation could be readily mitigated by employing orthogonal cysteine protection (e.g., Acm), which would be expected to further improve conversion efficiency (SI Section 3.2.3 and SI Figures 29–37). These observations highlight sequence-dependent differences in peptide reactivity, yet demonstrate that this enzymatic strategy provides a mild and selective approach for ArgTag removal, enabling access to native peptide sequences without the need for additional synthetic handles. To directly compare ArgTag against the previously reported SynTag approach, we synthesized GLP-1[7–37] on both systems. While the crude purity after SPPS was already higher with ArgTag (Figure 2C top, 71%) than with SynTag (Figure 2C middle, 60%), the MeDbz activation for SynTag removal introduced a major practical hurdle. Complete activation required 15 successive DIPEA washes, and the resulting crude peptide showed extensive formation of unidentifiable side-products (SI Figures 43–46). This led to a pronounced decrease in crude purity to 43% (including the peak area of the already formed native GLP-1[7–37]), likely severely complicating downstream purification (Figure 2C bottom). The ArgTag strategy, including the enzymatic removal of the C-terminal Arg residues, thus not only avoids the need for additional synthetic handles such as MeDbz but also delivers superior crude peptide quality and synthetic simplicity, enabling broader applicability and scalability.

**Figure 2:**
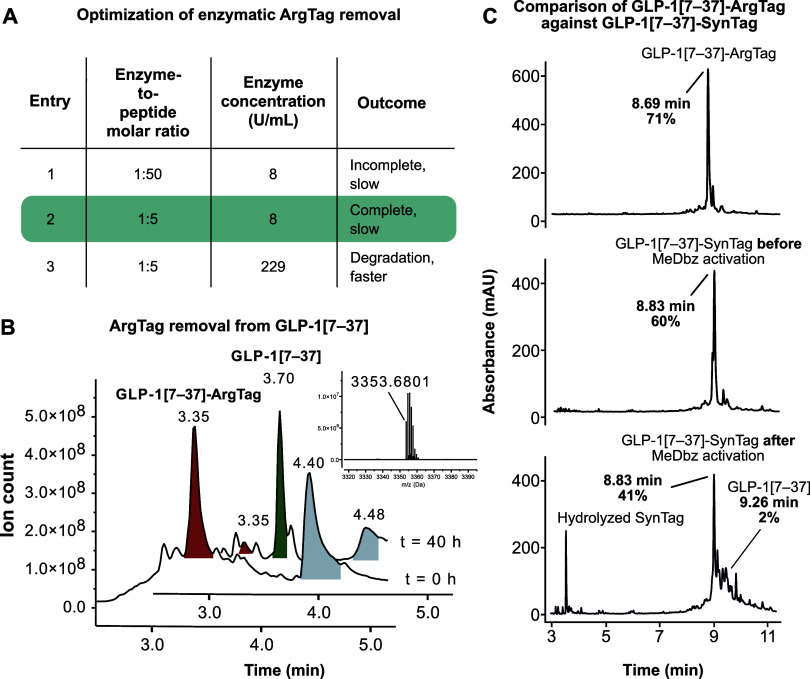
(A) Optimization table for ArgTag removal by CPB from GLP-1[7–37]. (B) Total ion count (TIC) chromatograms at time points 0 and 40 h. Red peak: starting peptide GLP-1[7–37]-ArgTag. Green peak: desired peptide GLP-1[7–37]. Gray peak: CPB. Insert: Deconvoluted mass spectrum of reaction mixture after 40 h at retention time 3.70 min (green peak). Calculated: 3353.6681, found: 3353.6801. (C) Comparison of crude purity of GLP-1[7–37]-ArgTag (top) against unactivated (middle) and activated (bottom) GLP-1[7–37]-SynTag (UHPLC; absorbance measured at 214 nm).

### C-Terminal ArgTag Improves the Synthesis of Aggregating Peptides Independently from Resin Type and Loading

We next investigated whether the ArgTag could be applied to a wide range of solid supports. For this purpose, Barstar[75–90] was used as a short and severely aggregating test peptide.^9^ Six different types of resins were tested at comparable loadings ranging from 0.20–0.35 mmol/g, all with a Rink amide linker (RAM). The range of resins extends from apolar polymers based on PS (Polystyrene-aminomethyl-RAM (PSAM-RAM) and methylbenzhydryl amine-RAM (MBHA-RAM)) to intermediate polarity resins consisting of PEG grafted on PS polymers (TentaGelXV-RAM and NovaGel-RAM) to polar resins based on PEG or piperazine linked polyacrylamide linked polymers (NovaPEG-RAM and LiQ-RAM, Figure 1B). We were pleased to observe delayed or suppressed aggregation on all resin types, as evidenced by in-line UV–vis data collected from the AFPS system (Figure 3A). Across all tested resins, the dashed lines corresponding to the synthesis of Barstar[75–90] show the typical steep increase in aggregation factor after coupling T[86]. However, incorporation of a C-terminal “ArgTag” (synthesis of Barstar[75–90]-ArgTag) results either in a delayed start of aggregation until after G[80] (MBHA-RAM) or E[81] (PSAM-RAM, NovaGel-RAM, LiQ-RAM) or in complete suppression of aggregation (TentaGelXV-RAM, NovaPEG-RAM).

**Figure 3:**
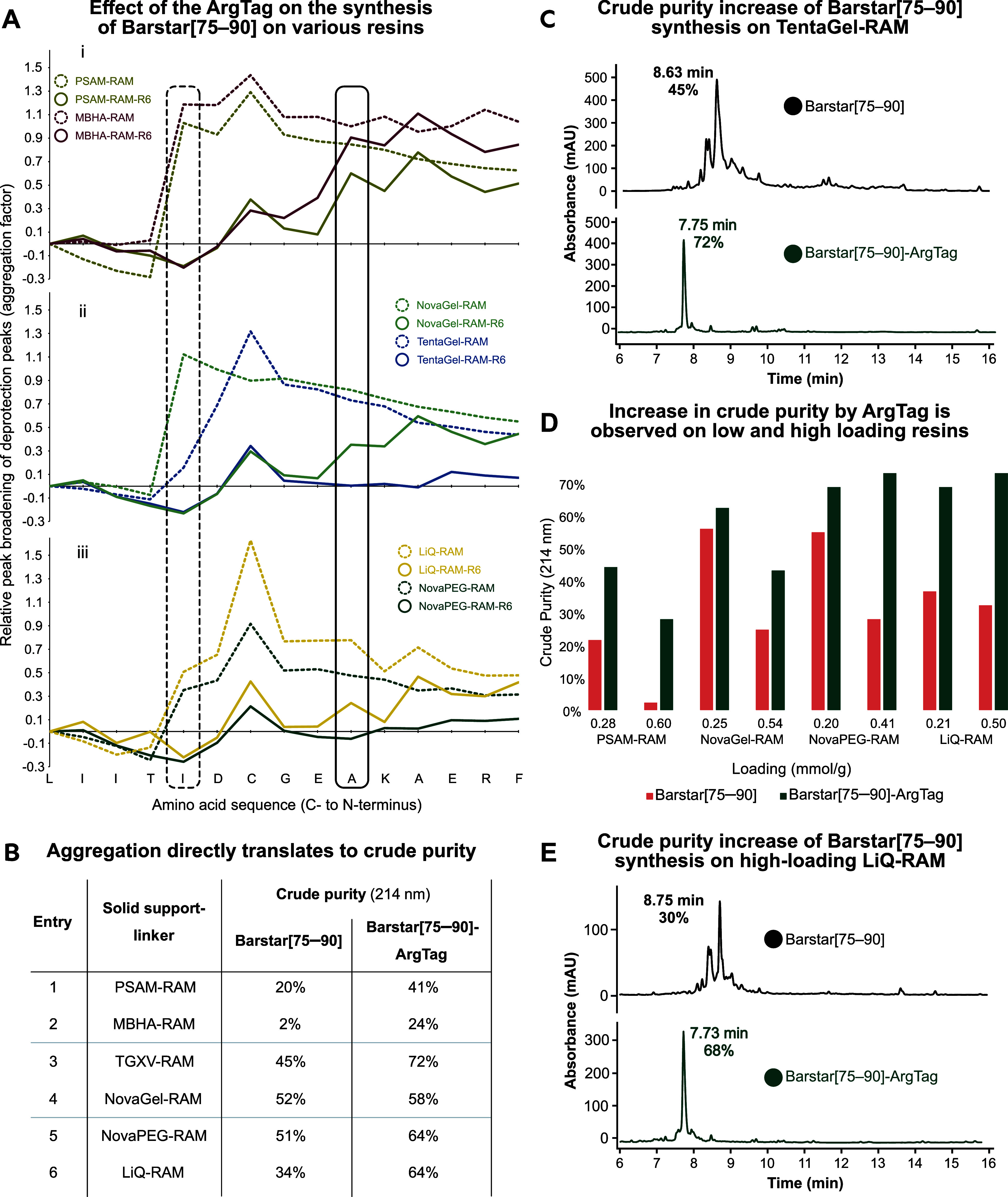
ArgTag improves crude peptide purity across several resins and resin loadings. (A) Aggregation as a function of Fmoc deprotection peak broadening monitored by in-line UV–vis (310 nm) for various resins. Solid lines represent syntheses of Barstar[75–90] with the ArgTag, and dashed lines represent syntheses without ArgTag. The aggregation factor was normalized to L[89], as the loading of several resins had to be reduced prior to synthesis. In the untagged sequence, Barstar[75–90] begins to aggregate after T[86] (dashed box). Addition of the ArgTag either delays the onset of aggregation or eliminates it entirely, with the delayed onset occurring after E[86] (solid box). The pronounced peak broadening observed during cysteine coupling reflects the reduced reaction temperature (60 °C vs 90 °C for other residues) and not a distinct aggregation event. (B) Delayed or suppressed aggregation directly translates to improved crude purity for all resins. Crude purity was determined via UHPLC (214 nm). (C) UHPLC of crude Barstar[75–90] and Barstar[75–90]-ArgTag synthesized on TentaGel-RAM resin. (D) ArgTag improves crude purity on all tested resin types independent of resin loading. (E) UHPLC of crude Barstar[75–90] and Barstar[75–90]-ArgTag synthesized on high-loading LiQ-RAM resin.

These observations also directly translate into substantially improved crude Barstar[75–90] purities on all resin types (Figure 3B,C, for remaining UHPLC traces see SI). The use of the ArgTag improves crude purity on apolar PS-based resins: The crude purity on PSAM-RAM resin was approximately doubled from 20 to 41% (Figure 3B, **entry 1**). Synthesis on MBHA-RAM resulted in a crude purity of 24% with a clearly defined peak in the UHPLC chromatogram when only trace amounts were obtained without the ArgTag (**entry 2**). As for the PEG–PS polymer mixed resins, good crude purities were obtained already without the ArgTag. Nonetheless, the synthesis on TentaGel XV-RAM particularly benefited from the aggregation-suppressing effect of the ArgTag, as evidenced by a substantial increase in crude purity from 45% to 72% (**entry 3**). A modest improvement from 52 to 58% was also observed on NovaGel-RAM (**entry 4**). Finally, among the most polar solid supports, NovaPEG-RAM outperformed LiQ-RAM without ArgTag (51 vs 34% crude purity). However, introduction of the ArgTag on LiQ-RAM results in a greater improvement in crude purity, reaching 64%—matching the performance of NovaPEG-RAM with the ArgTag (**entries 5 and 6**). In summary, the ArgTag consistently and significantly improved the crude purity of the aggregating sequence Barstar[75–90] across all resin categories.

To further demonstrate the versatility of the ArgTag, we investigated its ability to suppress aggregation on higher loading resins. This is particularly relevant for industrial applications, where high-loading resins are preferably used to reduce the consumption of hazardous and expensive solvents. For this investigation, we tested resins from each category at both low loading (manually lowered via acetyl capping) and high loading (as commercially available). In the apolar category, PSAM-RAM at 0.28 and 0.60 mmol/g was tested. NovaGel-RAM at loadings of 0.25 and 0.54 mmol/g was chosen in the intermediate polarity category, and the purely PEG-based resin NovaPEG-RAM at 0.20 and 0.41 mmol/g was investigated in the polar category along with LiQ-RAM resin at 0.21 and 0.50 mmol/g. Generally, known trends such as loading dependency of aggregation were confirmed, as demonstrated by higher crude purities for synthesis on low-loading resins.^21^ Importantly, the ArgTag also facilitated a cleaner synthesis on the high-loading variants of all resins evaluated. For both most polar resins (NovaPEG-RAM and LiQ-RAM), synthesis on the high-loading resins yielded higher crude purities of Barstar[75–90]-ArgTag than their low-loading counterparts (Figure 3D,E, for remaining UHPLC traces see SI). Interestingly, the improvement in crude purity is in general more pronounced on the higher loading versions of the resins compared to the low-loading ones, indicating the potential utility of the ArgTag on industrially relevant solid supports with comparably higher loading.

### ArgTag Improves the Synthesis of Aggregating Peptides Independent from Synthesis Scale and Conditions

To assess the preparative applicability of the ArgTag strategy, we next evaluated its performance on a pilot-scale flow synthesis platform (PeptiPilot, PeptiSystems AB). Compared to the AFPS system, which operates at 90 °C with short cycle times (∼3 min per residue) and high reagent excess (20–40 equiv), the PeptiPilot usually employs more economical conditions suggested by the manufacturer (2 equiv as a starting point for process optimization of unproblematic sequences). Because of the high complexity of the Barstar peptide, the couplings were performed at 50 °C with 5 equiv of amino acid, DIC/Oxyma as activating agents, and significantly longer cycle times (∼60 min per residue). For this investigation a 12.5 mL reactor column was used, allowing synthesis at substantially larger scale (Figure 4A). Using UV data collected from the PeptiPilot, we calculated and plotted the aggregation factor, following the same approach previously applied on the AFPS system.^27^ This analysis confirmed that Barstar[75–90] exhibits aggregation at the same point, whereas Barstar[75–90]-ArgTag shows reduced aggregation (Figure 4B), resulting in substantially improved crude purity (Figure 4C). Under these conditions, the synthesis of Barstar[75–90] on high-loading PS-2CTC resin (0.75 mmol/g) yielded a crude purity of 27%, which was improved to 50% for Barstar[75–90]-ArgTag on resin with a comparable loading (0.69 mmol/g) (SI Figures 107–112). The improved crude purity is primarily attributed to elevated coupling efficiencies at residues C[83] and D[84], which eliminates two major impurity peaks at 7.86 and 8.55 min, corresponding to acetyl truncations prior to C[83] and D[84], respectively. The impurity observed at 9.69 min in the Barstar[75–90]-ArgTag synthesis is identical to the peak at 13.66 min in the Barstar[75–90] synthesis and corresponds to an Fmoc truncation prior to D[84]. These findings are consistent with the aggregation behavior inferred from the aggregation factor analysis (Figure 4B).

**Figure 4:**
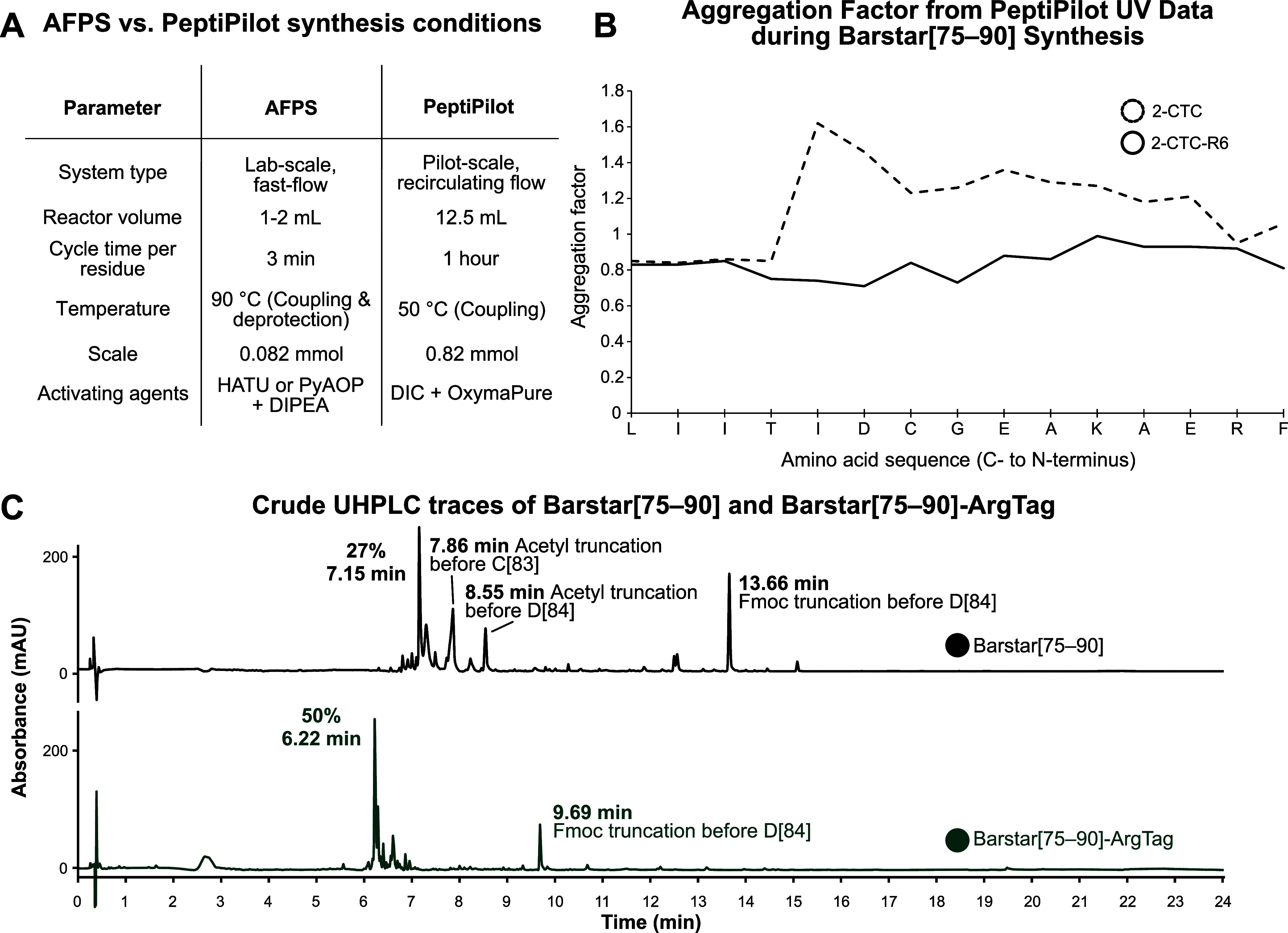
ArgTag improves peptide crude purity under scalable synthesis conditions. (A) Comparison table of synthesis conditions between AFPS and PeptiPilot systems. (B) Aggregation as a function of Fmoc deprotection peak broadening by in-line UV–vis (310 nm) recorded on the PeptiPilot system. The same point of aggregation after T[86] is observed as on the AFPS. Addition of ArgTag suppresses aggregation. (C) UHPLC chromatograms of crude Barstar[75–90] and Barstar[75–90]-ArgTag synthesized on PeptiPilot. The suppressed aggregation directly translates into an improved crude purity of Barstar[75–90]-ArgTag.

This result demonstrates that the ArgTag strategy remains effective under scalable synthesis conditions and with more economical reagents. Together with the improvements observed across diverse resins at varying loadings, this highlights the practical versatility of ArgTag and its potential for application in preparative peptide synthesis.

## Conclusions

In this study, we expanded and refined the use of a C-terminal hexaarginine tag (“ArgTag”) as a practical strategy to improve the synthesis of aggregation-prone peptides by SPPS. The ArgTag builds on our previously developed SynTag concept, offering a simplified, linker-free alternative with broader applicability. We first optimized a mild enzymatic method for ArgTag removal using high-purity recombinant CPB. This protocol enabled cleavage of the six C-terminal arginine residues under aqueous conditions, allowing recovery of the native peptide sequence without the need for an additional linker. We evaluated the ArgTag across six resin types spanning a range of polarities and loadings. In all cases, the ArgTag effectively suppressed aggregation and led to improved crude purities, including on commonly used high-loading PS-based resins. Importantly, the benefits were consistent across both low- and high-loading variants, underscoring the tag’s versatility. Finally, we validated the ArgTag strategy under preparative-scale synthesis conditions using the PeptiPilot platform. Despite significantly lower amino acid equivalents, milder temperatures, and longer cycle times compared to AFPS, the ArgTag still yielded improved crude purities, confirming its effectiveness under economical and scalable synthesis conditions. Altogether, the ArgTag provides a robust, removable aggregation-suppressing tool for SPPS that is compatible with a wide range of resins, synthesis scales, and process constraints. This approach offers a valuable solution for both academic and industrial peptide synthesis workflows targeting difficult sequences.

## Supplementary Material



## Data Availability

Data for this paper, including raw NMR files, LCMS, UHPLC and AFPS and PeptiPilot synthesis data are available at Zenodo at 10.5281/zenodo.15615282.

## Notes

Materials Detailed experimental procedures AFPS synthesis plots UHPLC chromatograms LCQTOF chromatograms and spectra ^1^H NMR spectra of compounds isolated from chemical ArgTag removal (PDF)

## References

[1] Kent S. B. H. (2009). Total
Chemical Synthesis of Proteins. Chem. Soc. Rev..

[2] Merrifield R. B. (1963). Solid Phase
Peptide Synthesis. I. The Synthesis of a Tetrapeptide. J. Am. Chem. Soc..

[3] Atherton, E. ; Sheppard, R. C. Detection of Problem Sequences in Solid Phase Synthesis. In Peptides, Structure and Function. Proceedings of the Ninth American Peptide Symposium; Pierce Chemical Company: Rockford, Illinois, US, 1985; pp 415–418.

[4] Sletten E. T., Nuño M., Guthrie D., Seeberger P. H. (2019). Real-Time
Monitoring of Solid-Phase Peptide Synthesis Using a Variable Bed Flow
Reactor. Chem. Commun..

[5] Saebi A., Brown J. S., Marando V. M., Hartrampf N., Chumbler N. M., Hanna S., Poskus M., Loas A., Kiessling L. L., Hung D. T., Pentelute B. L. (2023). Rapid Single-Shot
Synthesis of the 214 Amino Acid-Long N-Terminal Domain of Pyocin S2. ACS Chem. Biol..

[6] Hartrampf N., Saebi A., Poskus M., Gates Z. P., Callahan A. J., Cowfer A. E., Hanna S., Antilla S., Schissel C. K., Quartararo A. J., Ye X., Mijalis A. J., Simon M. D., Loas A., Liu S., Jessen C., Nielsen T. E., Pentelute B. L. (2020). Synthesis
of Proteins by Automated Flow Chemistry. Science.

[7] Mueller L. K., Baumruck A. C., Zhdanova H., Tietze A. A. (2020). Challenges and Perspectives
in Chemical Synthesis of Highly Hydrophobic Peptides. Front. Bioeng. Biotechnol..

[8] Sun H., Brik A. (2019). The Journey for the
Total Chemical Synthesis of a 53 KDa Protein. Acc. Chem. Res..

[9] Bürgisser H., Williams E. T., Jeandin A., Lescure R., Premanand A., Wang S., Hartrampf N. (2024). A Versatile
“Synthesis Tag”
(SynTag) for the Chemical Synthesis of Aggregating Peptides and Proteins. J. Am. Chem. Soc..

[10] Srisailam S., Kumar T. K. S., Srimathi T., Yu C. (2002). Influence of Backbone
Conformation on Protein Aggregation. J. Am.
Chem. Soc..

[11] Shivu B., Seshadri S., Li J., Oberg K. A., Uversky V. N., Fink A. L. (2013). Distinct β-Sheet Structure in Protein Aggregates
Determined by ATR-FTIR Spectroscopy. Biochemistry.

[12] Paradís-Bas M., Tulla-Puche J., Albericio F. (2016). The Road to the Synthesis of “Difficult
Peptides. Chem. Soc. Rev..

[13] Vaino A. R., Janda K. D. (2000). Solid-Phase Organic
Synthesis: A Critical Understanding
of the Resin. J. Comb. Chem..

[14] Lee M. A., Brown J. S., Loas A., Pentelute B. L. (2024). Investigation
of Commercially Available Resins for the Automated Flow Synthesis
of Difficult or Long Peptide Sequences. Pept.
Sci..

[15] Rodionov I. L., Peshenko I. A., Baidakova L. K., Ivanov V. T. (2007). Swellographic Study
of Peptide Resin Swelling Behavior during Solid Phase Peptide Synthesis. Int. J. Pept. Res. Ther..

[16] Wo T., Wahl F., Nefzi A., Rohwedder B., Sato T., Sun X., Mutter M. (1996). Pseudo-Prolines
as
a Solubilizing, Structure-Disrupting Protection Technique in Peptide
Synthesis. J. Am. Chem. Soc..

[17] Jensen, K. J. ; Shelton, T. P. ; Pedersen, S. L. Peptide Synthesis and Applications, 2nd ed.; Methods in Molecular Biology; Springer: New York, 2013; Vol. 1047. http://www.springer.com/series/7651.

[18] Quarrell R., Claridge T. D. W., Weaver G. W., Lowe G. (1995). Structure and Properties
of TentaGel Resin Beads: Implications for Combinatorial Library Chemistry. Mol. Divers..

[19] Akintayo D. C., de la Torre B. G., Li Y., Albericio F. (2022). Amino-Li-Resin—A
Fiber Polyacrylamide Resin for Solid-Phase Peptide Synthesis. Polymers.

[20] Mohapatra S., Hartrampf N., Poskus M., Loas A., Gómez-Bombarelli R., Pentelute B. L. (2020). Deep Learning for Prediction and Optimization of Fast-Flow
Peptide Synthesis. ACS Cent. Sci..

[21] Tamás B., Willi P. L., Bürgisser H., Hartrampf N. (2024). A Robust Data
Analytical Method to Investigate Sequence Dependence in Flow-Based
Peptide Synthesis. React. Chem. Eng..

[22] Blanco-Canosa J. B., Nardone B., Albericio F., Dawson P. E. (2015). Chemical Protein
Synthesis Using a Second-Generation N-Acylurea Linker for the Preparation
of Peptide-Thioester Precursors. J. Am. Chem.
Soc..

[23] Prosser L. C., Talbott J. M., Garrity R. P., Raj M. (2023). C-Terminal Arginine-Selective
Cleavage of Peptides as a Method for Mimicking Carboxypeptidase B. Org. Lett..

[24] Folk, J. E. [32] Carboxypeptidase B (Porcine Pancreas). In Methods in Enzymology; Elsevier, 1970; Vol. 19, pp 504–508 10.1016/0076-6879(70)19036-7.

[25] Simpson M. C., Harding C. J., Czekster R. M., Remmel L., Bode B. E., Czekster C. M. (2023). Unveiling the Catalytic
Mechanism of a Processive Metalloaminopeptidase. Biochemistry.

[26] Zisapel N., Kurn-Abramowitz N., Sokolovsky M. (1973). Basic and
Non-Basic Substrates of
Carboxypeptidase B. Eur. J. Biochem..

[27] Hartrampf N., Saebi A., Poskus M., Gates Z. P., Callahan A. J., Cowfer A. E., Hanna S., Antilla S., Schissel C. K., Quartararo A. J., Ye X., Mijalis A. J., Simon M. D., Loas A., Liu S., Jessen C., Nielsen T. E., Pentelute B. L. (2020). Synthesis of Proteins by Automated Flow Chemistry. Science.

