# Supplementary material for: Development of ArgTag for Scalable Solid-Phase Synthesis of Aggregating Peptides
Source: ACS Chem Biol. 2025 Oct 23;20(11):2733–40. doi: 10.1021/acschembio.5c00662 (PMC12645436; doi:10.1021/acschembio.5c00662)
Supplement: Supplementary file 1 [file cb5c00662_si_001.pdf]

Supplementary Material

Title: Development of ArgTag for scalable solid-phase synthesis of aggregating peptides

Authors: Vincent Freiburghaus[1], Aliénor Jeandin[1], Łukasz Frankiewicz[2], Jie Yang[2], Nina Hartrampf[1,*]

Affiliations:
[1] Department of Chemistry, University of Zurich, Winterthurerstrasse 190, 8057 Zurich, Switzerland

[2] PeptiSystems AB, Uppsala Business Park, Virdings allé 22, 754 50, Uppsala, Sweden

* Corresponding author. Email: nina.hartrampf@chem.uzh.ch

1 Table of Contents

1 Table of Contents 2
2 Material and general methods 3
2.1 Reagents and solvents 3
2.2 Peptide Synthesis 3
2.3 Coupling of Rink amide linker to SPPS resin 5
2.4 Manual lowering of resin loading 5
2.5 Determination of resin loading 5
2.6 TFA-mediated peptidyl-resin cleavage and global deprotection 6
2.7 Analytical Ultra-High Performance Liquid Chromatography (UHPLC) 6
2.8 Liquid Chromatography with High-Resolution Electrospray Ionization Quadrupole Time-of-Flight (LC-HR-ESI-QTOF) 6
2.9 Nuclear Magnetic Resonance Spectroscopy 7
2.10 Semi-Preparative Reverse-Phase High Performance Liquid Chromatography (RP-HPLC) 8
3 Removal of ArgTag 8
3.1 Chemical removal of ArgTag 8
3.2 Enzymatic ArgTag removal 15
4 Resin screening 41
4.1 Polystyrene aminomethyl Rink Amide (PSAM-RAM, 0.28 mmol/g loading): Barstar[75–90] 44
4.2 Polystyrene aminomethyl Rink Amide (PSAM-RAM, 0.28 mmol/g loading): Barstar[75–90]-ArgTag 46
4.3 Polystyrene aminomethyl Rink Amide (PSAM-RAM, 0.60 mmol/g loading): Barstar[75–90] 48
4.4 Polystyrene aminomethyl Rink Amide (PSAM-RAM, 0.60 mmol/g loading): Barstar[75–90]-ArgTag 50
4.5 Methylbenzhydryl amine Rink Amide (MBHA-RAM, 0.35 mmol/g loading): Barstar[75–90] 52
4.6 Methylbenzhydryl amine Rink Amide (MBHA-RAM, 0.35 mmol/g loading): Barstar[75–90]-ArgTag 53
4.7 Rink Amide TentagelXV (TGXV-RAM, 0.23 mmol/g loading): Barstar[75–90] 55
4.8 Rink Amide TentagelXV (TGXV-RAM, 0.23 mmol/g loading): Barstar[75–90]-ArgTag 57
4.9 Rink Amide NovaGel (NovaGel-RAM, 0.25 mmol/g loading): Barstar[75–90] 59
4.10 Rink Amide NovaGel (NovaGel-RAM, 0.25 mmol/g loading): Barstar[75–90]-ArgTag 61
4.11 Rink Amide NovaGel (NovaGel-RAM, 0.54 mmol/g loading): Barstar[75–90] 63
4.12 Rink Amide NovaGel (NovaGel-RAM, 0.54 mmol/g loading): Barstar[75–90]-ArgTag 64
4.13 Rink Amide LiQ (LiQ-RAM, 0.21 mmol/g loading): Barstar[75–90] 66
4.14 Rink Amide LiQ (LiQ-RAM, 0.21 mmol/g loading): Barstar[75–90]-ArgTag 68
4.15 Rink Amide LiQ (LiQ-RAM, 0.50 mmol/g loading): Barstar[75–90] 70
4.16 Rink Amide LiQ (LiQ-RAM, 0.50 mmol/g loading): Barstar[75–90]-ArgTag 72
4.17 Rink Amide NovaPEG (NovaPEG-RAM, 0.20 mmol/g loading): Barstar[75–90] 74
4.18 Rink Amide NovaPEG (NovaPEG-RAM, 0.20 mmol/g loading): Barstar[75–90]-ArgTag 76
4.19 Rink Amide NovaPEG (NovaPEG-RAM, 0.41 mmol/g loading): Barstar[75–90] 77
4.20 Rink Amide NovaPEG (NovaPEG-RAM, 0.41 mmol/g loading): Barstar[75–90] 79
5 Evaluation of ArgTag on PeptiPilot system 81
5.1 2-CTC polystyrene (PS-2 CTC, 0.75 mmol/g loading): Barstar[75–90] 81
5.2 2-CTC polystyrene (PS-2 CTC, 0.69 mmol/g loading): Barstar[75–90]-ArgTag 83
6 References 85

2 Material and general methods

2.1 Reagents and solvents

Fmoc- and side chain-protected L-amino acids (Fmoc-Ala-OH, Fmoc-Arg(Pbf)-OH, Fmoc-Asn(Trt)-OH, Fmoc-Asp(O*t*Bu)-OH, Fmoc-Cys(Trt)-OH, Fmoc-Gln(Trt)-OH, Fmoc-Glu(O*t*Bu)-OH, Fmoc-Gly-OH, Fmoc-His(Trt)-OH, Fmoc-Ile-OH, Fmoc-Leu-OH, Fmoc-Lys(Boc)-OH, Fmoc-Met-OH, Fmoc-Phe-OH, Fmoc-Pro-OH, Fmoc-Ser(*t*Bu)-OH, Fmoc-Thr(*t*Bu)-OH, Fmoc-Trp(Boc)-OH, Fmoc-Tyr(*t*Bu)-OH, Fmoc-Val-OH) were purchased from Bachem AG; *O*-(7-azabenzotriazol-1-yl)-*N,N,N',N'*-tetramethyluronium hexafluorophosphate (HATU) was purchased from Bachem AG and (7-azabenzotriazol-1-yloxy)tripyrrolidinophosphonium hexafluorophosphate (PyAOP) were purchased from Advanced ChemTech CreoSalus; *N,N*-diisopropylethylamine (*i*Pr_2NEt, DIPEA, 99.5%) and acetic anhydride (Ac_2O, ≥98%) were purchased from Sigma-Aldrich; trifluoroacetic acid (TFA, for HPLC, ≥99.0%), triisopropylsilane (TIPS, 98%) and 3,6-dioxa-1,8-octane-dithiol (DODT, 95%) were purchased from Sigma-Aldrich. *N,N*-Dimethylformamide (DMF) was purchased from VWR International (Avantor) and treated for >24 h with AldraAmine trapping packets purchased from Sigma-Aldrich; dichloromethane (DCM, ≥99.8%) was purchased from Fisher Scientific Ltd.; diethyl ether was purchased from Honeywell Riedel-de Haën; acetonitrile (MeCN, for HPLC gradient grade, ≥99.9%) was purchased from Sigma-Aldrich. NovaPEG Rink Amide resin (0.41 or 0.20 mmol/g loading) and Rink Amide NovaGel resin (0.54 mmol/g loading) were purchased from the Novabiochem-line from Sigma-Aldrich Canada Ltd; 2-CTC-PS resin (1.06 mmol/g loading) was purchased from Sigma-Aldrich. TentagelXV Rink amide resin (0.23 mmol/g loading) and Polystyrene Aminomethyl Rink Amide resin (0.60 mmol/g loading) was purchased from Rapp Polymere GmbH; LiQ Amino Resin (0.50 mmol/g loading) was provided by Iris Biotech GmbH; Rink Amide MBHA resin (0.35 mmol/g loading) was purchased from Fluorochem Ltd.. 4-[(2,4-Dimethoxyphenyl)(Fmoc-amino)methyl]phenoxyacetic acid (Fmoc-Rink amide linker-OH) was purchased from Fluorochem Ltd. Carboxypeptidase B from porcine pancreas was purchased from Sigma-Aldrich (Product No. 217356). Recombinant Carboxypeptidase B was purchased from Sigma-Aldrich (product of Roche CustomBiotech, Material No. 03358682103, Lot No. 84572500, HPLC purity 95%).

2.2 Peptide Synthesis

2.2.1 Automated flow-based peptide synthesis (AFPS)

Peptides were synthesized on an automated-flow system built in the Hartrampf lab, which is similar to the published AFPS system.[1] Capitalized letters refer to L-amino acids. Unless otherwise noted, the following settings were used for peptide synthesis: flow rate = 20 mL/min for coupling and deprotection steps (as specified), wherein the reactor base (containing resin) was kept at 90 °C, with pre-activation at 90 °C or 60 °C (heating loop) as specified. The standard synthetic cycle involves a first step of prewashing the resin at 90 °C for 60 s at 20 mL/min. During the coupling step, three HPLC pumps are used: a 50 mL/min pump head pumps the activating agent, a second 50 mL/min pump head pumps the amino acid, and a 5.0 mL/min pump head pumps *i*Pr_2NEt (*neat*). The 50 mL/min pump head pumps delivered 0.398679 mL of liquid per pump stroke, the 5.0 mL/min pump head pumps 3.9239×10^{-2} mL of liquid per pump stroke.

All peptides were prepared by AFPS on the specified resin and standard Fmoc/*t*Bu protected amino acids (0.40 M in DMF, 0.20 M final concentration) were coupled using HATU (0.38 M in DMF, 0.19

M final concentration) or PyAOP (0.38 M in DMF, 0.19 M final concentration) with DIPEA (delivered *neat*, approx. 0.27 M final concentration). The aggregation factor (AF) was calculated from in-line UV–Vis chromatograms recorded at 310 nm as the difference between peak width at half maximum (w) and peak height (h). For comparability across different syntheses, AF values were normalized to a reference deprotection step to yield relative AF values. In this work, the first common amino acid deprotection between a sequence and its ArgTag-derivative was used as the reference step.

2.2.2 AFPS method at 20 mL/min flow rate

For 20 mL/min method, for amino acids D, E, F, G, I, K, L a total volume of 6.4 mL of the "coupling solution" (i.e., amino acid [0.20 M], HATU or PyAOP [0.19 M], and DIPEA [0.27 M] in DMF) was applied for each coupling. For amino acids A, C, R, S, T, a total of 10.4 mL of "coupling solution" was applied for each coupling. Removal of the N^{α}-Fmoc group was achieved using 20% piperidine with 1% formic acid in DMF (6.4 mL, *v*/*v*/*v*) at a flow rate of 20 mL/min with preheating at 90 °C for all Fmoc-protected amino acids except C, for which preheating of the deprotection solution was at 60 °C. Between each coupling and deprotection step, the resin was washed with DMF (32 mL) at a flow rate of 20 mL/min with preheating at 90 °C for all amino acids except C, for which the DMF was preheated at 60 °C. After completion of the peptide sequence, the resins were manually washed with DCM (3 × 5 mL) and dried under reduced pressure.

2.2.3 Pilot scale peptide synthesis in flow on a PeptiPilot system

Evaluation of the ArgTag at higher synthesis scales and lower amino acid equivalents were performed on a PeptiPilot system by PeptiSystems AB. The instrument is equipped with a 12.5 mL reactor column. Couplings are performed at 50 °C using amino acid stock solutions of 0.4 M in DMF (5.0 eq.). Stock solutions of *N, N*-diisopropylcarbodiimide (DIC, 1.0 M, 10.0 eq.) and OxymaPure (0.5 M, 5.0 eq.) in DMF are used as activating agents. Amino acid solutions are pre-activated in a mixer (start flow 10 mL/min, maximum flow 40 mL/min, flow increment 1.0 mL/min) for 5 min then transferred to the reactor column (mixer-to-column flow rate 2.05 mL/min) and then recirculated through the reactor column for 20 min (circulation flow rate 16.36 mL/min). Subsequently, the capping mix (acetic anhydride 0.3 M, pyridine 0.3 M in DMF) flows through the reactor column for 5 min (capping flow rate 8.18 mL/min). Fmoc deprotection is achieved by flowing 20% piperidine in DMF through the reactor column (deprotection flow rate 8.18 mL/min) until the UV trace at 294 nm falls below 80 mAU or a maximum of 10 column volumes of deprotection solution are delivered. In addition to that, the flow stops, and the resin is incubated for 10 min in 20% piperidine in DMF to ensure complete deprotection before the reactor column is washed with DMF. After complete synthesis, the resin is removed from the reactor and washed with DCM, before being dried under airflow and in vacuum.

2.2.4 Batch Solid-Phase Peptide Synthesis (Batch-SPPS)

If required to enable resin loading determination, the first amino acid was coupled *via* batch-SPPS without subsequent Fmoc deprotection. The resin loading was confirmed using the protocol described in **Section 2.4**.

Unless otherwise noted, the amino resin (50 mg, 21 µmol, 1.0 eq.) was swelled with DCM (1 × 5 mL) for 1 min, drained, and washed with DMF (1 × 5 mL). For each coupling, a solution of Fmoc- and side-chain-protected amino acid (0.50 mL, 0.20 M in DMF, 5.0 eq.) and HATU

(0.50 mL, 0.19 M in DMF, 4.8 eq.) was prepared. To this solution, DIPEA (36 μL, 0.20 mmol, 10 eq.) was added, and the solution was gently agitated at 23 °C for 1 min. The solution was then added to the resin, and the reaction was gently stirred for 20 s, then left at 23 °C for 30 min. The resin was then drained, washed with DMF (3 × 5 mL) and DCM (3 × 5 mL). For each deprotection step, 20% piperidine in DMF (*v*/*v*) (3.0 mL) was added to the resin, and the reaction was gently stirred for 20 s then left at 23 °C for 5 min. The resin was then washed with DMF (5 mL) and the deprotection step was repeated. The resin was then drained, then washed with DMF (3 × 5 mL) and DCM (3 × 5 mL).

2.3 Coupling of Rink amide linker to SPPS resin

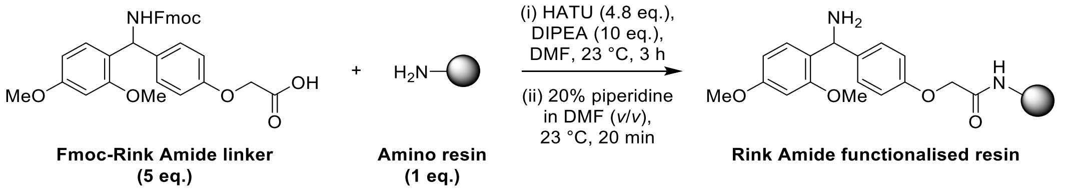

SI Figure 1. Coupling of Fmoc-Rink Amide linker to an amino resin.

Whenever manual Rink amide coupling was required, the following protocol was used: The amino resin (1.0 eq.) was swelled with DCM (1 × 5 mL) for 1 min, washed with DMF (1 × 5 mL) and the solvent was then removed by filtration under reduced pressure. To a solution of Fmoc-Rink linker (0.15 M in DMF, 5 eq.), HATU (0.38 M in DMF, 4.8 eq.) and DIPEA (10 eq.) were added, and the solution was gently agitated at 23 °C for 1 min. The solution was then added to the resin and the reaction was gently agitated at 23 °C for 3 h (**SI Figure 1**). Then the resin was drained, washed with DMF (3 × 5 mL) and DCM (3 × 5 mL), and dried under reduced pressure. Then, a Fmoc-loading test was performed as described in **Section 2.4**. Finally, the Fmoc group was removed by treating the resin with a solution of 20% piperidine in DMF (*v*/*v*) (5.0 mL, 3 × 5 min). The resin was then drained, washed with DMF (2 × 5 mL) and DCM (2 × 5 mL), then dried under reduced pressure.

2.4 Manual lowering of resin loading

To adjust higher loading resins to a similar level as the lower loading resins for screening of the ArgTag, Rink Amide linker resins with known loading were manually downloaded by acetic anhydride capping according to the following protocol:

The specific resin (1.0 eq.) was swelled and suspended with DCM (1 × 5 mL) for 1 min. DIPEA (2.0 eq.) was added neat to the suspension before Ac_2O (X eq., equivalents depending on the desired degree of lowering the resin loading; e.g. if the initial loading of 0.60 mmol/g needs to be lowered to 0.30 mmol/g X is 0.5 eq.) was added neat. The mixture was gently agitated at 23 °C for 5 min. The resin was then drained, washed with DCM (3 × 5 mL) and dried under reduced pressure. A Fmoc-protected amino acid was then coupled according to the procedure in **Section 2.2.4** and then the loading was determined according to the procedure in **Section 2.5.** The Fmoc group was removed by treating the resin with a solution of 20% piperidine in DMF (*v*/*v*) (5.0 mL, 2 × 5 min). The resin was then drained, washed with DMF (3 × 5 mL) and DCM (3 × 5 mL), then dried under reduced pressure.

2.5 Determination of resin loading

Three samples of the resin (2–3 mg each, accurately weighed to three significant figures) were treated with a solution of 20% piperidine in DMF (*v/v*) (1.5 mL) and left to stand at 23 °C for 20 min with occasional agitation. UV absorbance (λ = 290 nm) of each sample was then measured in triplicate, with 20% piperidine in DMF (*v/v*) as a blank. The loading was calculated with the following equation:

$$\frac{Absorbance\ measurement}{Mass\ of\ resin\ (mg) \times 1.75} = Fmoc\ loading\ (mmol/g)$$

Wherein 'Absorbance measurement' is the mean average of triplicates for each sample. The final loading value was obtained by mean average of the calculated loadings from the three resin samples.

2.6 TFA-mediated peptidyl-resin cleavage and global deprotection

The peptides were cleaved using a solution of TFA/TIPS/DODT/H_2O (94:1:2.5:2.5, *v/v/v/v*, 1–3 mL) for 2 h at 23 °C with gentle mixing. TFA was then removed by evaporation under a light stream of N_2, and the peptides were precipitated and isolated by centrifugation from ice-cold diethyl ether (2 × 15 mL), twice. The resulting peptide pellets were then briefly dried under a light stream of N_2, then dissolved in an aqueous solution containing 10–50% MeCN and 0.1% TFA, and lyophilized. Crude peptides were then analyzed by LC-QTOF and UHPLC.

2.7 Analytical Ultra-High Performance Liquid Chromatography (UHPLC)

For determination of purity by UHPLC, the filtered peptide solution was diluted in 10–50% acetonitrile (MeCN) in water with 0.1% TFA (500 μL) to a final concentration of approximately 0.5 mg/mL. The samples were analyzed on Agilent 1290 Infinity II Series, using Agilent OpenLab CDS and ChemStation software.

For standard analysis of all peptide samples, analytical UHPLC spectra were recorded on an analytical Agilent Zorbax 300SB-C18 RRHD column (2.1 mm × 50 mm, 1.8 μm particle size) kept at 40 °C, at a flow rate of 0.80 mL/min with UV detection at 214 nm. A binary solvent system was used, wherein Solvent A was 5% MeCN in 95% water with 0.1% TFA, and Solvent B was 95% MeCN containing 5% water and 0.1% TFA. A linear gradient of 0–100% Solvent B, corresponding to 5–95% MeCN, over 20 min (*ca.* 4.5% MeCN/min). At the end of the gradient, 100% Solvent B was pumped at 0.80 mL/min for 3 min. Then, the column was re-equilibrated at 0% Solvent B for 5 min. Purities of the crude and purified peptides were determined by ChemStation integration of all UHPLC signals at 214 nm within of 3–20 min (20 min gradient).

2.8 Liquid Chromatography with High-Resolution Electrospray Ionization Quadrupole Time-of-Flight (LC-HR-ESI-QTOF)

For determination of peptide masses and purity by LC-HR-ESI-QTOF, the filtered peptide solution was diluted in 10–50% acetonitrile (MeCN) in water with 0.1% TFA (60–500 μL) to a final concentration of approximately 0.01 mM. The samples were analyzed on an Agilent 1290 Infinity II Series UHPLC, which is connected to an Agilent 1260 Infinity II Series VWD, and an Agilent 6546 LC/Q-TOF.

For standard analysis of all peptide samples, LC-HR-ESI-QTOF spectra were recorded on an Agilent Zorbax 300SB-C18 HPLC column (2.1 × 150 mm, 5 μm particle size, **gradient A**) or on an Agilent Poroshell 300SB-C8 HPLC column (2.1 x 75 mm, 5 μm particle size, **gradient B**) kept

at 50 °C at a flow rate of 0.80 mL/min with UV detection at 214 nm and an injection volume of 5 μL. A binary solvent system was used, wherein Solvent A was 5% MeCN in water containing 0.1% formic acid, and Solvent B was 5% water in MeCN containing 0.1% formic acid.

LC-HR-ESI-QTOF Gradient A: Isocratic at 0% Solvent B for 3 min, then linear gradient of 0–100% Solvent B over 10 min, followed by isocratic at 100% for 2 min.

LC-HR-ESI-QTOF Gradient B: Isocratic at 0% Solvent B for 1.5 min, then linear gradient of 0–100% Solvent B over 10 min, followed by isocratic at 100% for 2 min.

UV spectra were recorded at 1.2 nm resolution and 20 points s^{-1}; Ion source parameters for ESI were: positive ionization mode, capillary voltage 3.5 kV, nozzle voltage 1 kV, gas temperature 320 °C, N_2 drying gas flow 8 L/min, nebulizer pressure 35 psi, sheath gas temperature 350 °C, N_2 sheath gas flow 11 L/min, fragmentor voltage 100 V, and skimmer voltage 65 V. Parameters for the mass analyzer in MS (Seg) mode were: mass range 10–3200 *m/z* with an acquisition rate of 1 spectra/sec and time of 1000 ms/spectra; Mass calibration took place using the Agilent low-concentration tune mix.

All mass spectra show deconvoluted masses from the raw *m/z* values, calculated using Mestrelab Research S.L.© MestReNova v. 14.1 Mnova MS Suite. Purity based on LCMS was calculated by calculating the Area Under the Curve (AUC) of desired product peak as a percentage of the AUC of all peaks (within 3–13 min) of the absorbance chromatogram (λ = 214 nm). Monoisotopic and average masses were calculated using ChemDraw Version 18.2.

2.9 Nuclear Magnetic Resonance Spectroscopy

NMR spectra were recorded at 298 K on an AV2 400 Bruker spectrometer. The spectra are calibrated to the residual ^{1}H and ^{13}C signals of the solvents. Chemical shifts are reported in ppm with the resonance resulting from incomplete deuteration of the solvent as the internal standard. Multiplicities are abbreviated as follows: singlet (s), doublet (d), triplet (t), quartet (q), doublet-doublet (dd), quintet (quint), multiplet (m), and broad (b).

2.10 Semi-Preparative Reverse-Phase High Performance Liquid Chromatography (RP-HPLC)

Semi-preparative RP-HPLC was performed on a Shimadzu prominence HPLC system (Shimadzu Corp., Japan) with a CBM-40 system controller module, an FRC-10A fraction collector, two LC-20AR pumps, and an SPD-40 UV/VIS detector, using an Agilent Zorbax 300SB-C18 Semi-Preparative column (9.4 × 250 mm, 5 µm particle size) kept at 23 °C, with a flow rate of 3.5 mL/min. A binary solvent system was used, wherein Solvent A was H_2O containing 0.1% TFA, and Solvent B was MeCN containing 0.1% TFA. Purifications were executed using the following gradient:

> **Semi-preparative RP-HPLC gradient:** Isocratic at 5% Solvent B for 5 min, then linear gradient of 5–95% Solvent B over 60 min, followed by isocratic at 95% for 5 min.

3 Removal of ArgTag

3.1 Chemical removal of ArgTag

Chemical removal of the ArgTag was performed following a modified procedure from *Prosser* et al.[2] The peptide bearing the ArgTag (1.17 µmol, 1.0 eq) was dissolved in H_2O (758 µL). 9,10-phenanthrenequinone (21.14 µmol, 18 eq.) was dissolved in MeCN (142 µL) and added to the peptide solution before adding 100 µL of NaOH solution (0.8 M). The reaction solution was gently agitated at 37 °C for 3 h. The reaction solution was then acidified with 10 µL of TFA and analyzed by LC-QTOF.

3.1.1 AKFALFA-ArgTag

```
                10
H2N-AKFALFARRR RRR-CONH2      (13 AA)
```

Removal of the ArgTag from AKFALFA-ArgTag was performed according to the method described in **Section 3.1**. Crude AKFALFA-ArgTag (synthesized according to method described in **Section 2.2.2**) was used (**SI Figure 2**) and the reaction was complete after 3 h (**SI Figure 3**). Besides the desired peptide, the expected fluorophore side product **2** was detected alongside excess of reagent **1** and an unexpected side product **3**. We hypothesized that **3** can be formed through a benzyilic rearrangement of **1**. ^{1}H-NMR data (**SI Figure 4**) collected after isolating **3** supports this hypothesis.

LC-QTOF of crude AKFALFA-ArgTag

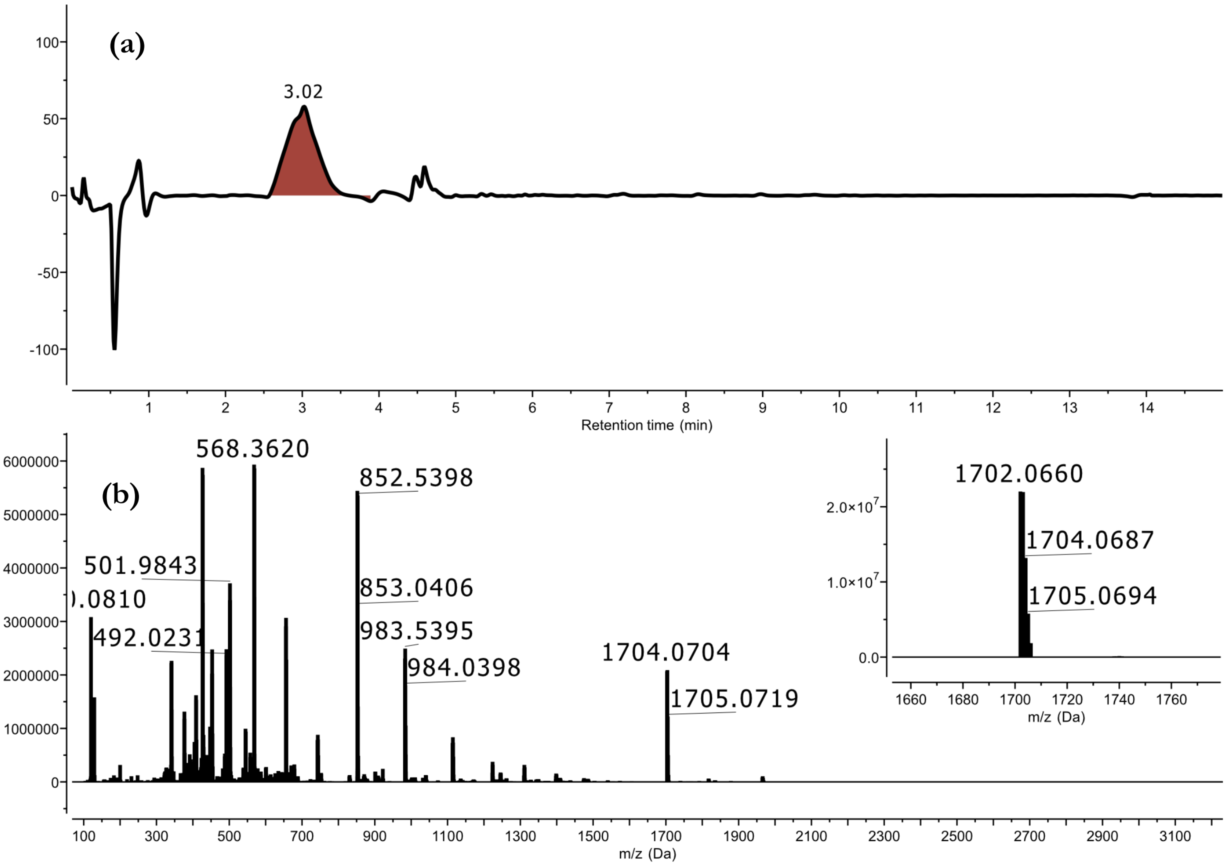

SI Figure 2. LC-HR-ESI-QTOF Profile of crude AKFALFA bearing the ArgTag. (a) UV chromatogram (214 nm) of crude AKFALFA-ArgTag; Rt 3.02 min. (b) ESI-TOF spectrum found within Rt 3.02 min (insert: deconvoluted masses). Monoisotopic mass (ESI+) calcd. for $C_{75}H_{131}N_{33}O_{13}$ 1702.0604, found 1702.0660. LCMS Gradient A (**Section 2.8**).

LC-QTOF of reaction mixture after 3 h

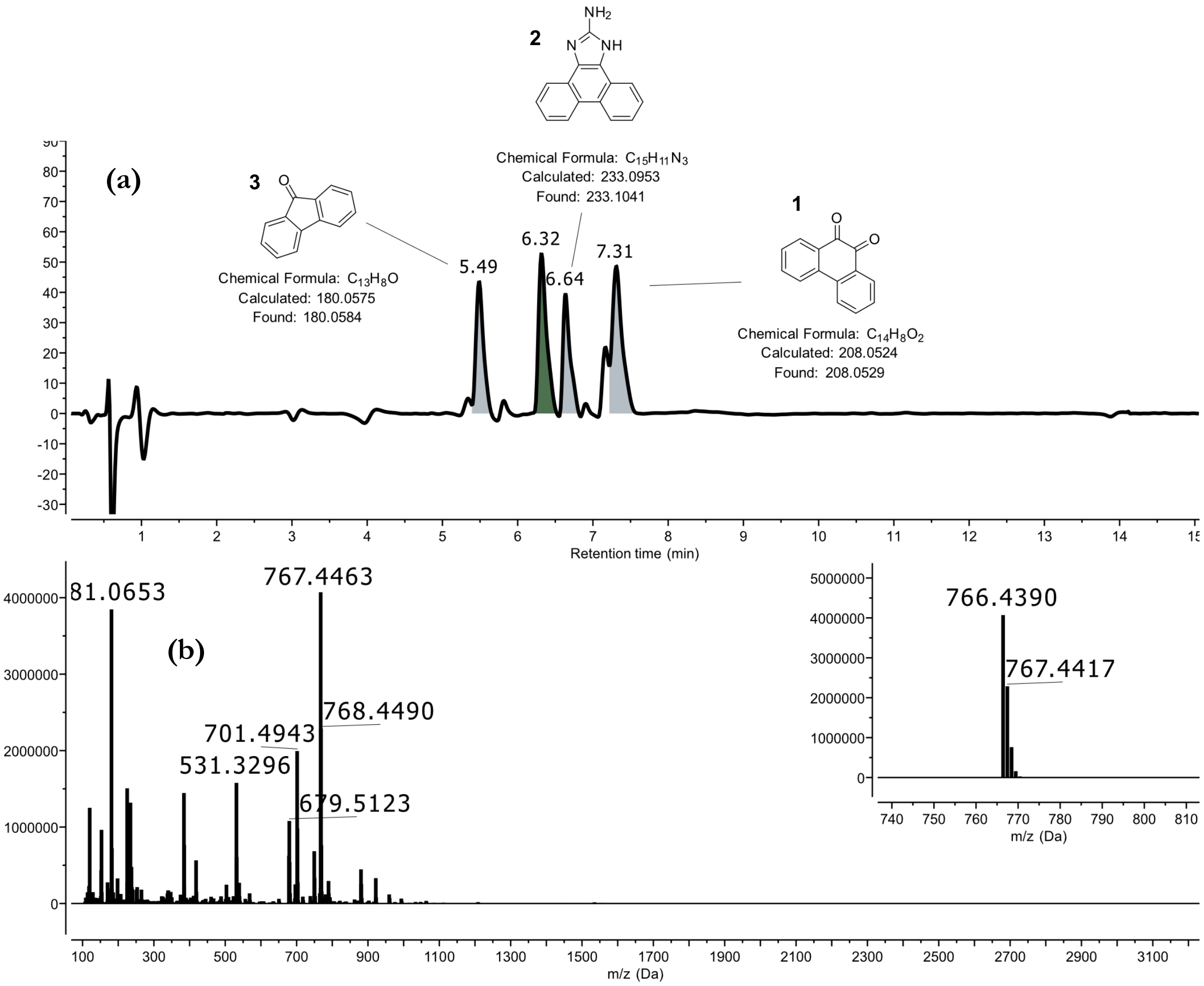

SI Figure 3. LC-HR-ESI-QTOF Profile of reaction mixture after 3 h reaction time. (a) UV chromatogram (214 nm) showing crude AKFALFA; Rt 6.32 min. Unexpected side product 3; Rt 5.49 min (grey). Expected fluorophore side product 2; Rt 6.64 (grey). Excess reagent 1; Rt 7.31(grey). (b) ESI-TOF spectrum found within Rt 6.32 min (insert: deconvoluted masses). Monoisotopic mass (ESI+) calcd. for $C_{39}H_{58}N_8O_8$ 766.4378, found 766.4390. LCMS Gradient A (**Section 2.8**).

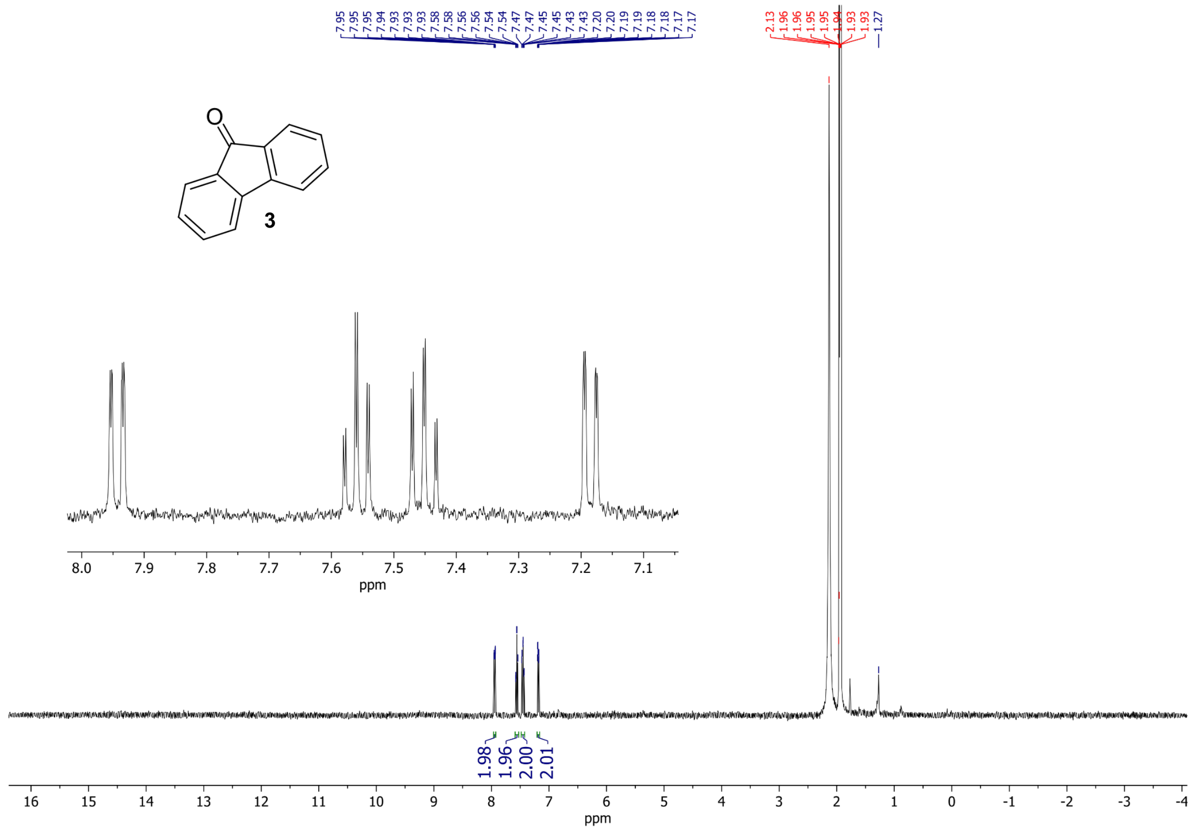

SI Figure 4. ^{1}H-NMR of isolated **3** recorded in MeCN-*d3.*

^{1}H NMR (400 MHz, CD_3CN): δ 7.96 – 7.92 (ddd, *J* = 7.6, 1.4, 0.5 Hz, 2H), 7.56 (td, *J* = 7.5, 1.4 Hz, 2H), 7.45 (td, *J* = 7.7, 1.4 Hz, 2H), 7.19 (ddd, *J* = 7.6, 1.4, 0.5 Hz, 2H).

3.1.2 EFGIKLNGTWIY-ArgTag

```
                 10
H2N-EFGIKLNGTW IYRRRRRR-CONH2    (18 AA)
```

Removal of the ArgTag from EFGIKLNGTWIY-ArgTag was performed according to the method described in **Section 3.1** with 6.0 eq. of 9,10-phenanthrenequinone. Crude EFGIKLNGTWIY - ArgTag (synthesized according to method described in **Section 2.2.2**) was used (**SI Figure 5**) and the reaction was complete after 3 h (**SI Figure 6**). Besides the desired peptide, the expected fluorophore side product **2** was detected alongside excess of reagent **1** and the side product **3**. Other side products could not be identified.

LC-QTOF of crude EFGIKLNGTWIY-ArgTag

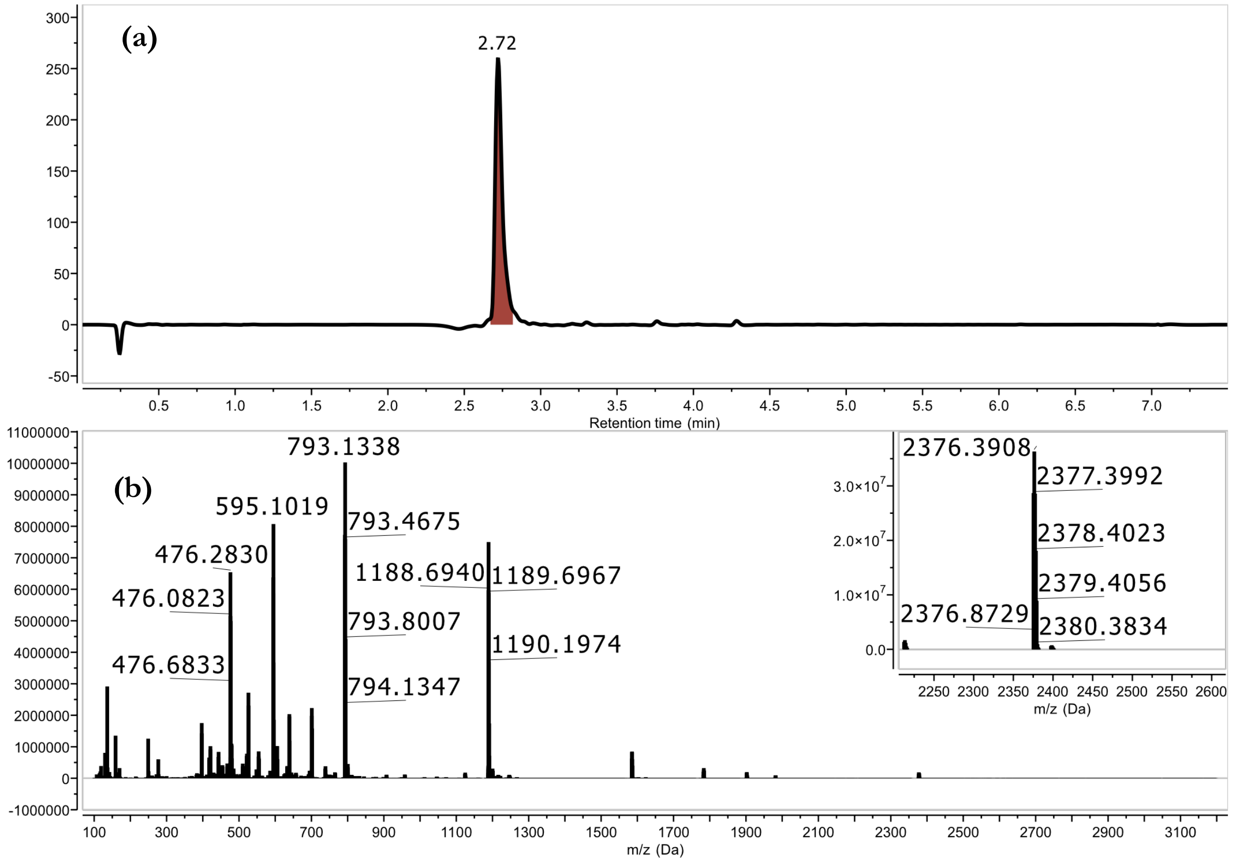

SI Figure 5. LC-HR-ESI-QTOF Profile of crude EFGIKLNGTWIY bearing the ArgTag. (a) UV chromatogram (214 nm) of crude EFGIKLNGTWIY-ArgTag; Rt 2.72 min. (b) ESI-TOF spectrum found within Rt 2.72 min (insert: deconvoluted masses). Monoisotopic mass (ESI+) calcd. for $C_{106}H_{174}N_{40}O_{23}$ 2375.3676, found 2375.3876. LCMS Gradient B (**Section 2.8**).

LC-QTOF of reaction mixture after 3 h

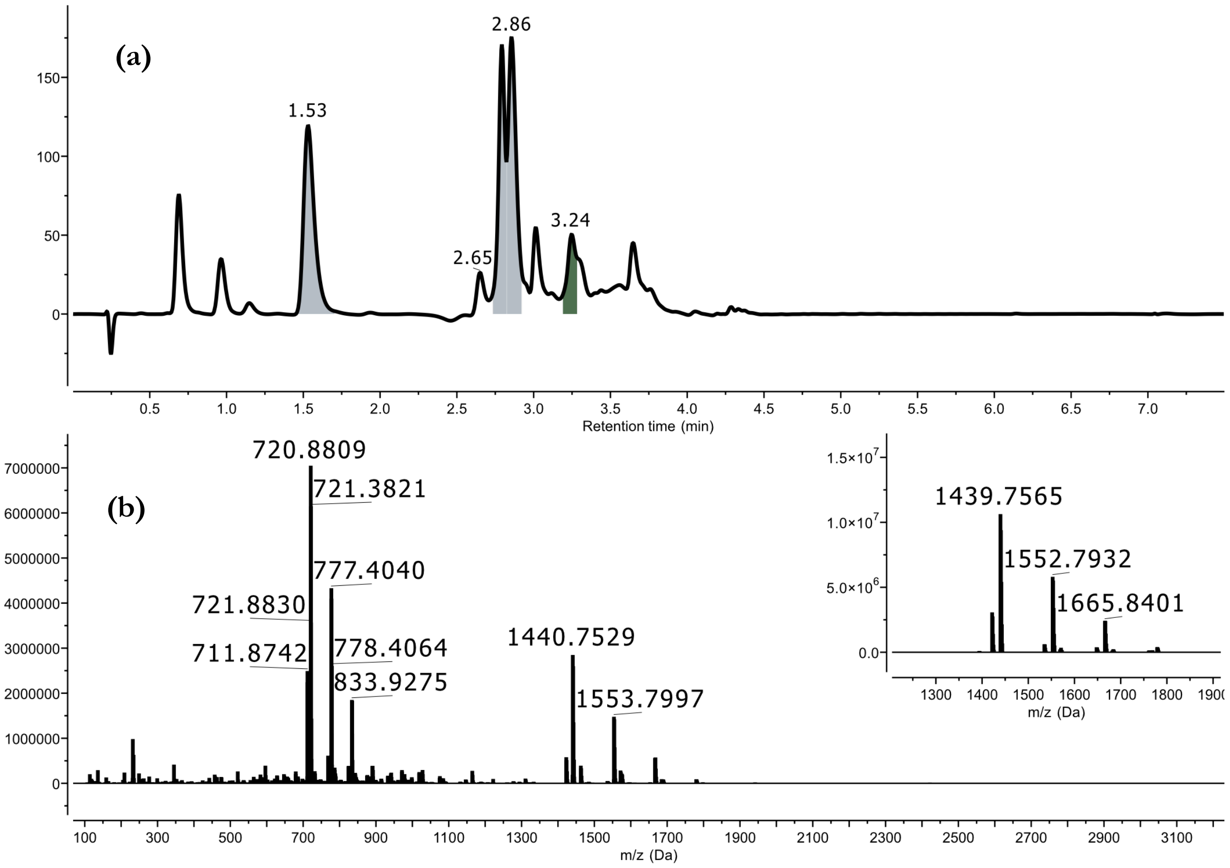

SI Figure 6. LC-HR-ESI-QTOF Profile of reaction mixture after 3 h reaction time. (a) UV chromatogram (214 nm) showing crude EFGIKLNGTWIY; Rt 3.24 min (green). Unexpected side product 3; Rt 1.53 min (grey). Expected fluorophore side product 2; Rt 2.79 min (grey). Excess reagent 1; Rt 2.86 min (grey). (b) ESI-TOF spectrum found within Rt 3.24 min (insert: deconvoluted masses). Monoisotopic mass (ESI+) calcd. for $C_{70}H_{101}N_{15}O_{18}$ 1439.7449, found 1439.7565. LCMS Gradient B (**Section 2.8**).

3.1.3 Barstar[75–90]-ArgTag

```
             10         20
H2N-FREAKAEGCD ITIILSRRRR RR-CONH2        (22 AA)
```

Removal of the ArgTag from Barstar[75–90]-ArgTag was performed according to the method described in **Section 3.1** with 6.0 eq. of 9,10-phenanthrenequinone. Purified Barstar[75–90]-ArgTag (according to method described in **Section 2.9**) was used (**SI Figure 7**) and no remaining starting material could be detected after 1 h (**SI Figure 8**). Under the conditions described, C[83] is oxidized to cysteic acid and R76 is converted to the corresponding aldehyde (**SI Figure 9**). The expected fluorophore side product **2** was detected alongside excess of reagent **1** and the side product **3**. Other side products could not be identified.

LC-QTOF of purified Barstar[75–90]-ArgTag

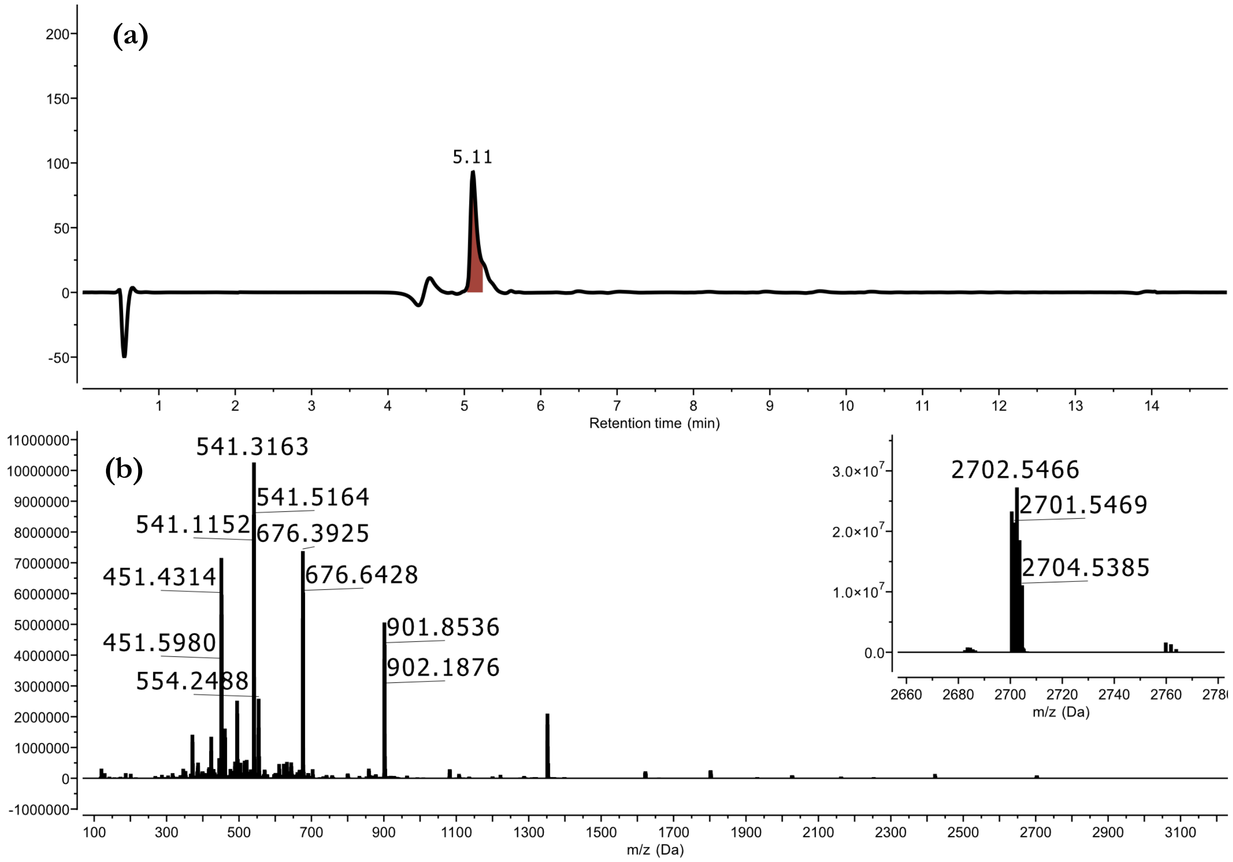

SI Figure 7. LC-HR-ESI-QTOF Profile of purified Barstar[75–90] bearing the ArgTag. (a) UV chromatogram (214 nm) of purified Barstar[75–90]-ArgTag; Rt 5.11 min. (b) ESI-TOF spectrum found within Rt 5.11 min (insert: deconvoluted masses). Monoisotopic mass (ESI+) calcd. for $C_{113}H_{201}N_{45}O_{30}S$ 2700.5307, found 2700.5442. LCMS Gradient A (**Section 2.8**).

LC-QTOF of reaction mixture after 1 h

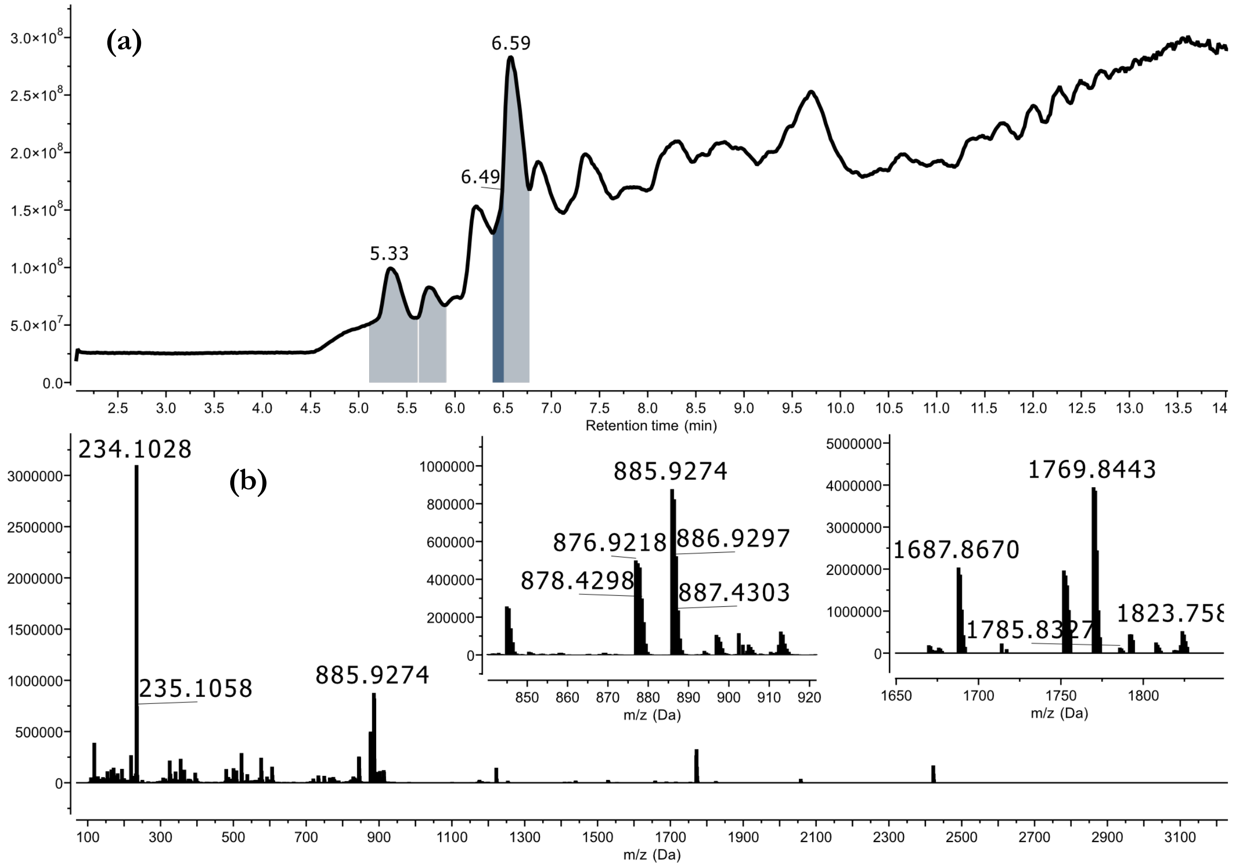

SI Figure 8. LC-HR-ESI-QTOF Profile of reaction mixture after 1 h reaction time. (a) TIC chromatogram showing crude oxidized Barstar[75–90] derivative; Rt 6.49 min (blue). Unexpected side product 3; Rt 5.33 min (grey). Excess reagent 1; Rt 5.73 min (grey). Expected fluorophore side product 2; Rt 6.59 min (grey). (b) ESI-TOF spectrum found within Rt 6.49 min (left insert: zoom into the mass spectrum; right insert: deconvoluted masses). Monoisotopic mass (ESI+) calcd. for $C_{76}H_{123}N_{17}O_{29}S$ 1769.8393, found 1769.8443. LCMS Gradient A (**Section 2.8**).

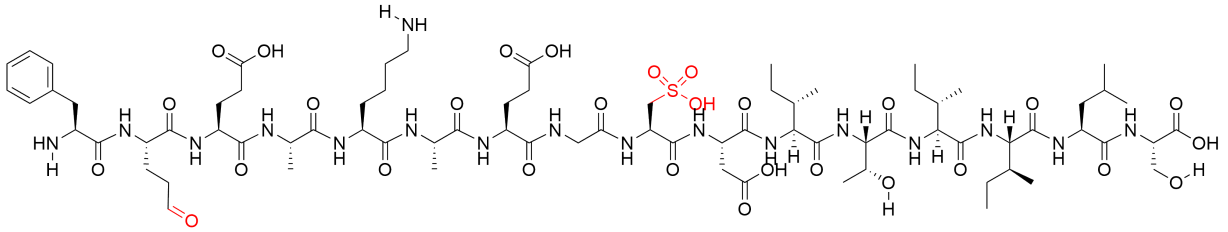

Chemical Formula: $C_{76}H_{123}N_{17}O_{29}S$
Calculated: 1769.8393
Found: 1769.8443

SI Figure 9. Proposed structure of oxidized Barstar[75–90] derivative. Highlighted in red are the amino acid side chains of Arg and Cys which did not withstand the experimental conditions and were converted into an aldehyde and cysteic acid, respectively.

3.2 Enzymatic ArgTag removal

3.2.1 Barstar[75–90]-ArgTag

Enzymatic removal of the ArgTag from crude Barstar[75–90]-ArgTag was performed by using Carboxypeptidase B isolated from porcine pancreas (152 U/mg specific activity) in a 1:20 molar ratio compared to Barstar[75–90]-ArgTag in TrisHCl buffer (pH 8.0 at 23 °C).[3] The final enzyme concentration was 3 µM (16 U/mL), the final peptide concentration 60 µM. The reaction solution

was incubated at 37 °C for 2 h until no remaining Barstar[75–90]-ArgTag could be detected by LC-QTOF (**SI Figure 10 & 11**).

LC-QTOF of reaction mixture before incubation (t = 0 min)

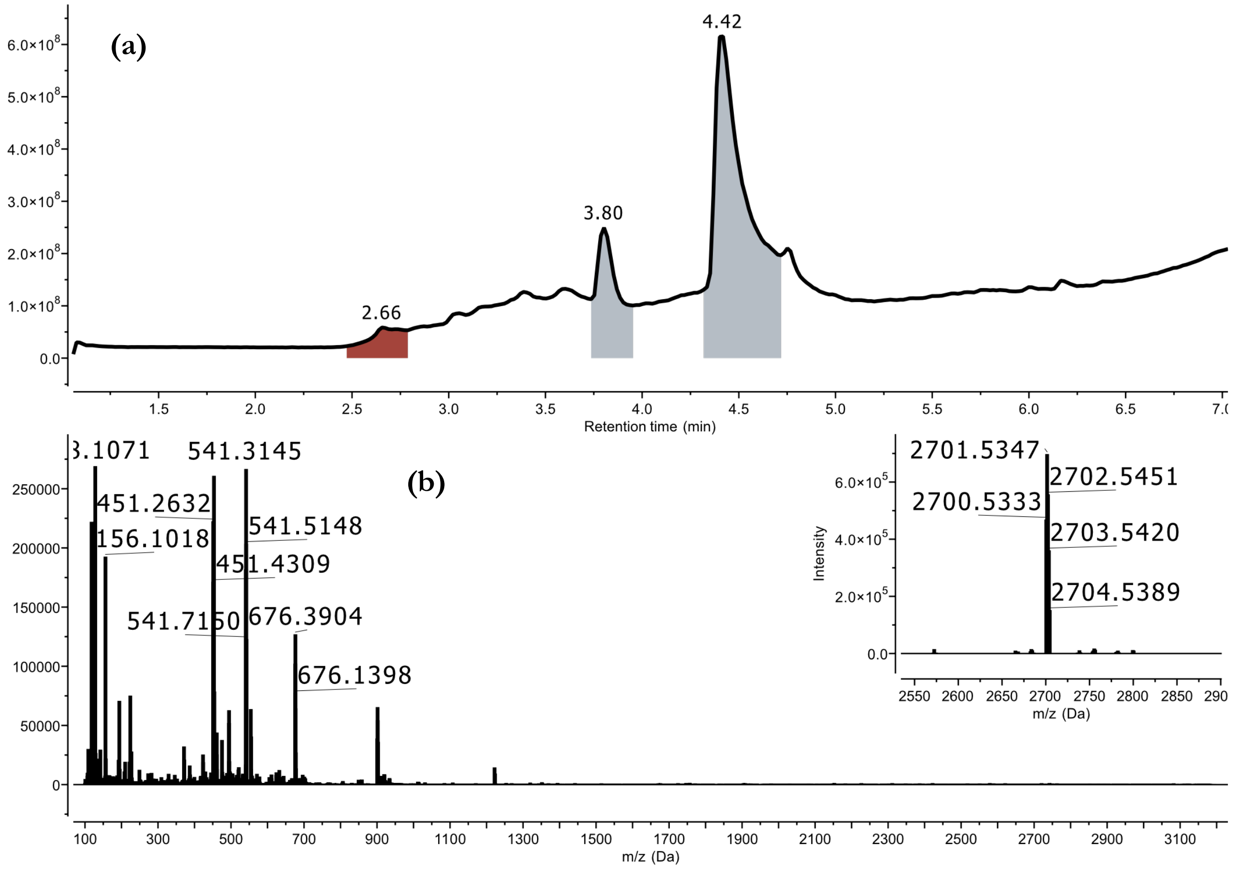

SI Figure 10. LC-HR-ESI-QTOF Profile of reaction mixture before incubation (t = 0 min). (a) TIC of crude Barstar[75–90]-ArgTag derivative (red); Rt 2.66 min. Enzyme peaks at Rt 3.80 min and 4.42 min (grey). (b) ESI-TOF spectrum found within Rt 2.66 min (insert deconvoluted masses). Monoisotopic mass (ESI+) $C_{113}H_{201}N_{45}O_{30}S$ 2700.5307, found 2700.5347. LCMS Gradient B (**Section 2.8**).

LC-QTOF of reaction mixture after incubation (t = 120 min)

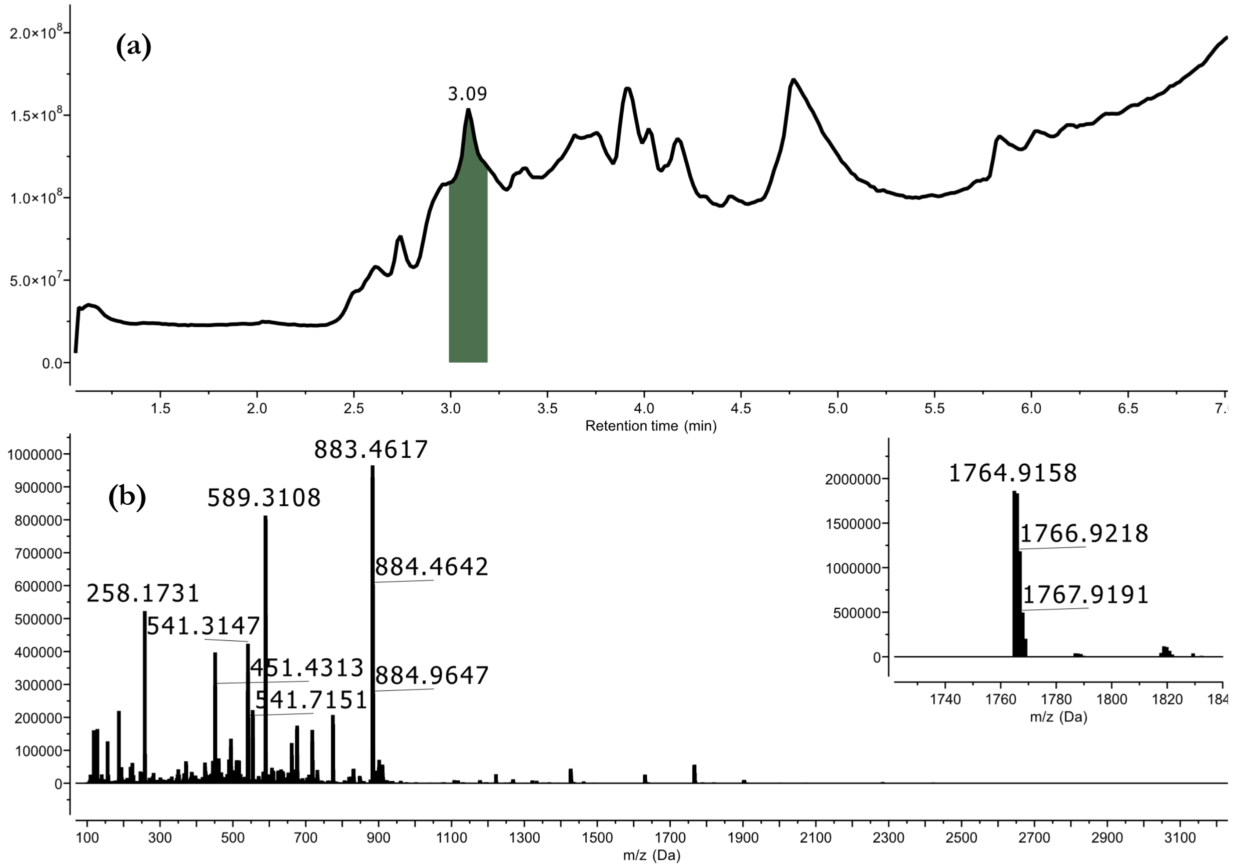

SI Figure 11. LC-HR-ESI-QTOF Profile of reaction mixture after incubation (t = 120 min). (a) TIC showing crude Barstar[75–90] (green); Rt 3.09 min. (b) ESI-TOF spectrum found within Rt 3.09 min (insert: deconvoluted masses). Monoisotopic mass (ESI+) $C_{77}H_{128}N_{20}O_{25}S$ 1764.9080, found 1764.9158. LCMS Gradient B (**Section 2.8**). No more starting peptide could be detected. The enzyme was not detected anymore.

3.2.2 GLP-1[7–37]-ArgTag

```
                10              20                 30
H2N-HAEGTFTSDV  SSYLEGQAAK   EFIAWLVKGR GRRRRRR-CONH2   (37 AA)
```

The peptide GLP-1[7–37]-ArgTag was synthesized on commercially available Rink Amide NovaPEG resin (0.41 mmol/g, 150.5 mg, 62 μmol) using the standard AFPS protocol (**Section 2.2.2**, **SI Figure 12**). Total synthesis time to afford resin-bound GLP-1[7–37]-ArgTag was approximately 1.5 h. Cleavage of the peptidyl-resin (51 mg, approx. 21 μmol) according to the cleavage protocol described in **Section 2.6** afforded the crude peptide as a colorless solid (11.0 mg, mass confirmed by LC-HR-ESI-QTOF [**SI Figure 13**], 71% purity by UHPLC [**SI Figure 14**]). Enzymatic removal of the ArgTag from purified GLP-1[7–37]-ArgTag (according to method described in **Section 2.9**) was performed by using recombinant Carboxypeptidase B (254 U/mg specific activity) in a 1:5 molar ratio compared to GLP-1[7–37]-ArgTag in TrisHCl buffer (pH 8.0 at 23 °C).[3] The final enzyme concentration was 0.9 μM (8 U/mL), the final peptide concentration 4.6 μM. The reaction solution was incubated at 37 °C for 40 h until no more GLP-1[7–37]-ArgTag could be detected by LC-QTOF (**Conditions 2**, **SI Figures 19 – 23**). For non-optimized conditions 1, the molar ratio of enzyme-to-peptide was 1:50 and the enzyme concentration was 0.9 μM (8

U/mL) (**SI Figures 15 – 18)**. For non-optimized conditions 3, the molar ratio of enzyme-to-peptide was 1:5 and the enzyme concentration was 26 μM (229 U/mL) (**SI Figures 24 – 28)**.

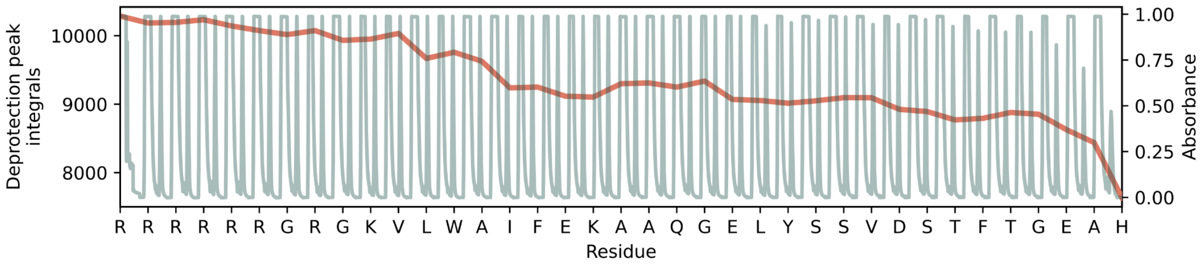

SI Figure 12. UV trace (λ = 310 nm) from AFPS of GLP-1[7–37]-ArgTag (green) and deprotection peak integrals (red). Note: UV chromatograms are plotted against time and are not directly aligned with the sequence labels on the x-axis. Only the integrals of the deprotection peaks are aligned with the corresponding amino acid positions.

LC-QTOF of crude GLP-1[7–37]-ArgTag

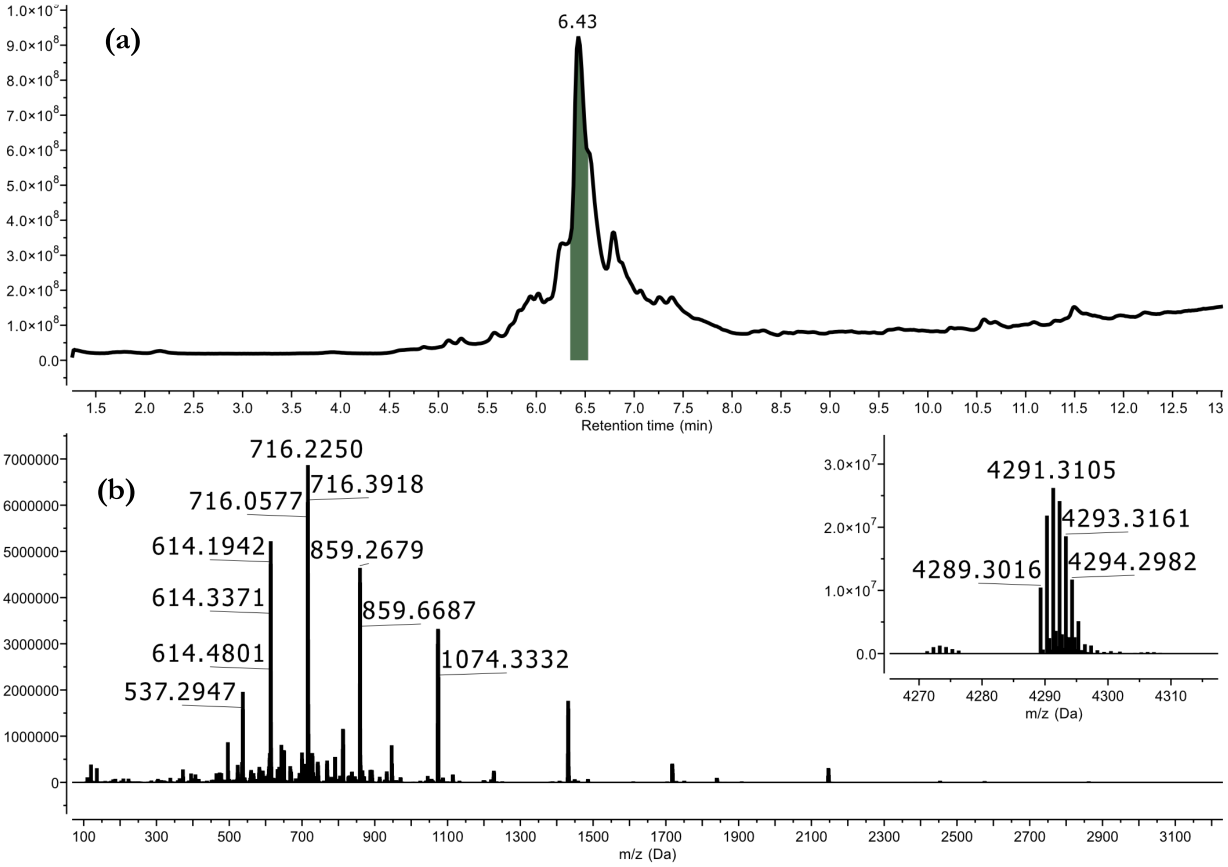

SI Figure 13. LC-HR-ESI-QTOF Profile of crude GLP-1[7–37] bearing the ArgTag. (a) TIC chromatogram of GLP-1[7–37]-ArgTag; Rt 6.43 min. (b) ESI-TOF spectrum found within Rt 6.43 min (insert: deconvoluted masses). Monoisotopic mass (ESI+) calcd. for $C_{187}H_{301}N_{65}O_{52}$ 4289.2907, found 4289.3016. LCMS Gradient A (**Section 2.8**).

UHPLC of crude GLP-1[7–37]-ArgTag

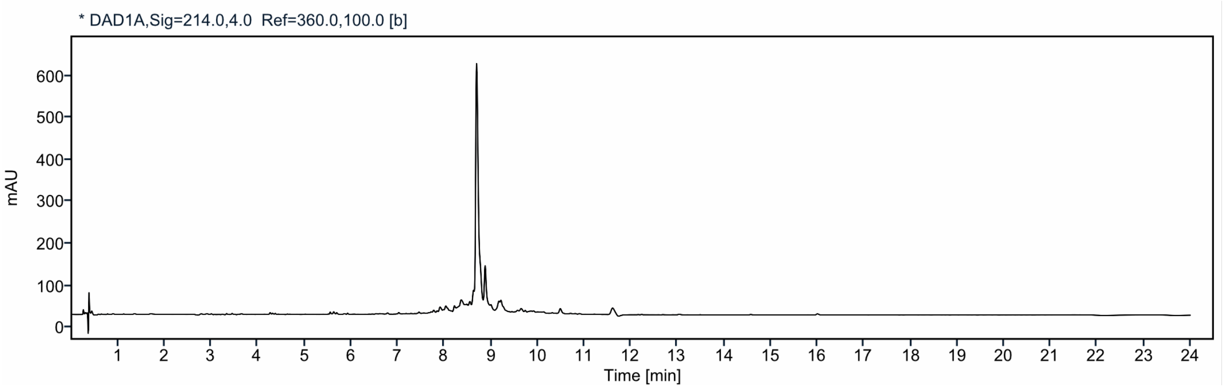

SI Figure 14. UHPLC profile of crude GLP-1[7–37] bearing ArgTag. Rt 8.69 min (Agilent Zorbax 300SB-C18 RRHD column, 1.8 µm, 2.1 × 50 mm, 5–95% MeCN over 20 min, ca. 4.5%B/min), 71% purity based on Area Under Curve (AUC) at λ = 214 nm.

Conditions 1: 1:50 molar ratio of enzyme-to-peptide, 8 U/mL enzyme concentration

LC-QTOF of reaction mixture before incubation (t = 0 min)

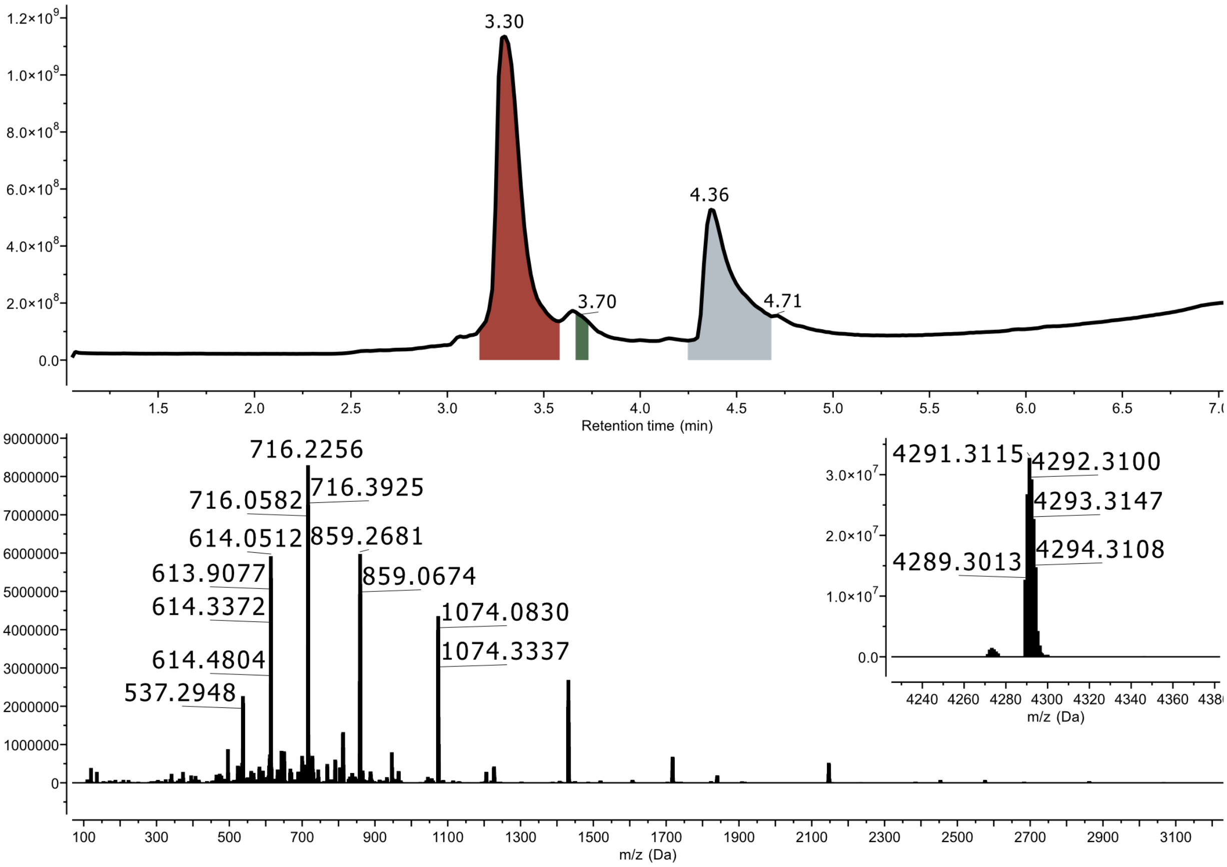

SI Figure 15. LC-HR-ESI-QTOF Profile of reaction mixture before incubation (t = 0 min). (a) TIC showing purified GLP-1[7–37]-ArgTag (red); Rt 3.30 min. Desired peptide GLP-1[7–37] (green); Rt 3.70 min. Enzyme peak at Rt 4.36 min (grey). (b) ESI-TOF spectrum found within Rt 3.30 min (insert deconvoluted masses). Monoisotopic mass (ESI+) $C_{187}H_{301}N_{65}O_{52}$ 4289.2907, found 4289.3013. LCMS Gradient B (**Section 2.8**).

LC-QTOF of reaction mixture after incubation (t = 3 h)

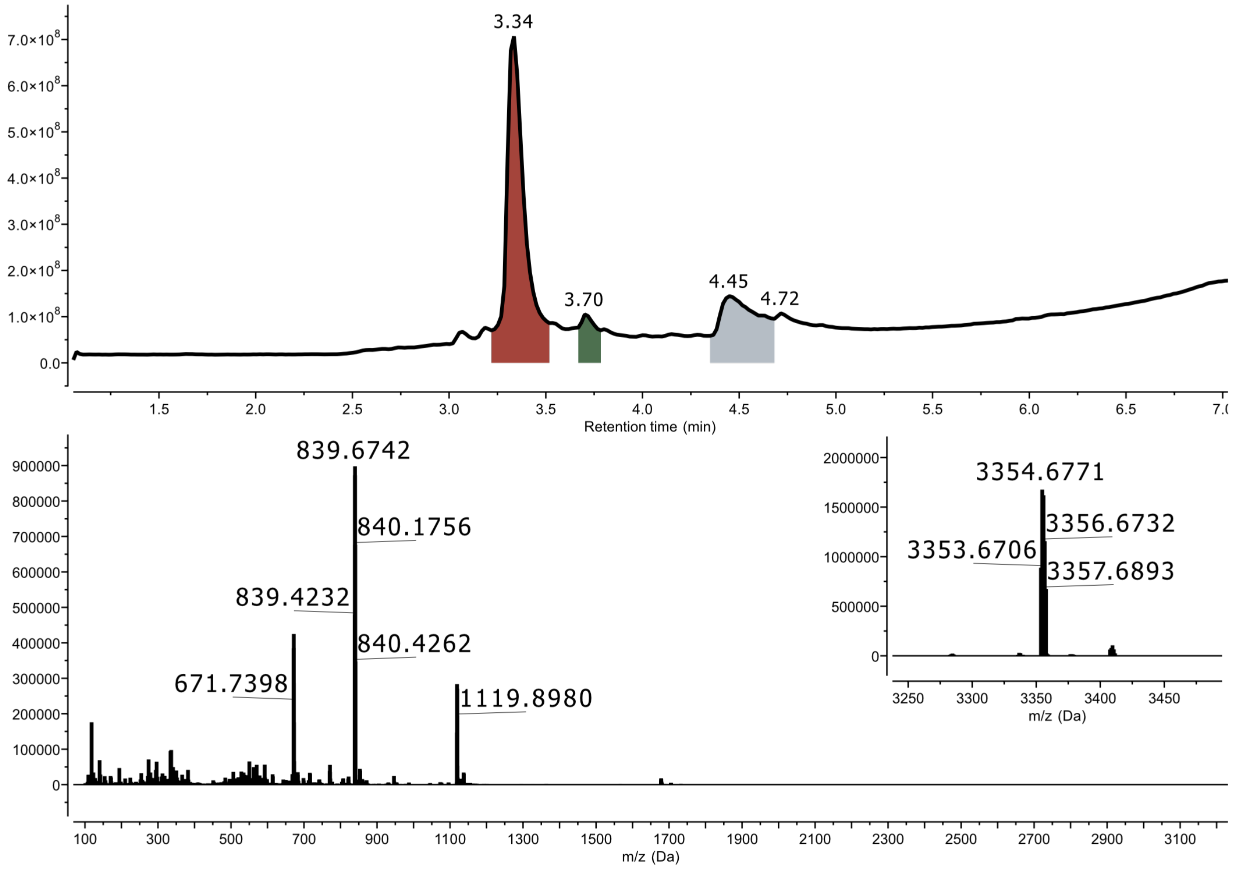

SI Figure 16. LC-HR-ESI-QTOF Profile of reaction mixture after incubation (t = 3 h). (a) TIC showing purified GLP-1[7–37]-ArgTag (red); Rt 3.34 min. Desired peptide GLP-1[7–37] (green); Rt 3.70 min. Enzyme peak at Rt 4.45 min (grey). (b) ESI-TOF spectrum found within Rt 3.70 min (insert deconvoluted masses). Monoisotopic mass (ESI+) $C_{151}H_{228}N_{40}O_{47}$ 3353.6681, found 3353.6706. LCMS Gradient B (**Section 2.8**).

LC-QTOF of reaction mixture after incubation (t = 24 h)

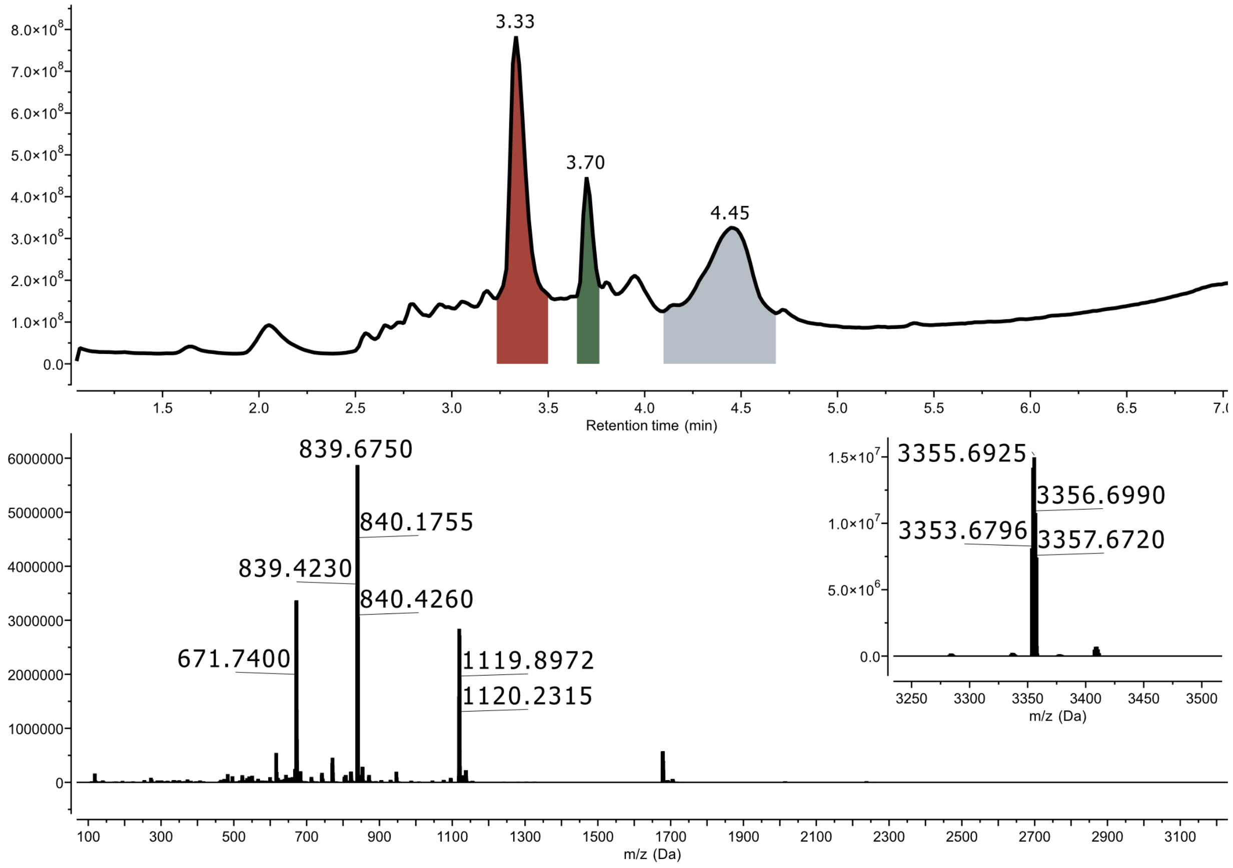

SI Figure 17. LC-HR-ESI-QTOF Profile of reaction mixture after incubation (t = 24 h). (a) TIC showing purified GLP-1[7–37]-ArgTag (red); Rt 3.33 min. Desired peptide GLP-1[7–37] (green); Rt 3.70 min. Enzyme peak at Rt 4.45 min (grey). (b) ESI-TOF spectrum found within Rt 3.70 min (insert deconvoluted masses). Monoisotopic mass (ESI+) $C_{151}H_{228}N_{40}O_{47}$ 3353.6681, found 3353.6796. LCMS Gradient B (**Section 2.8**).

LC-QTOF of reaction mixture after incubation (t = 40 h)

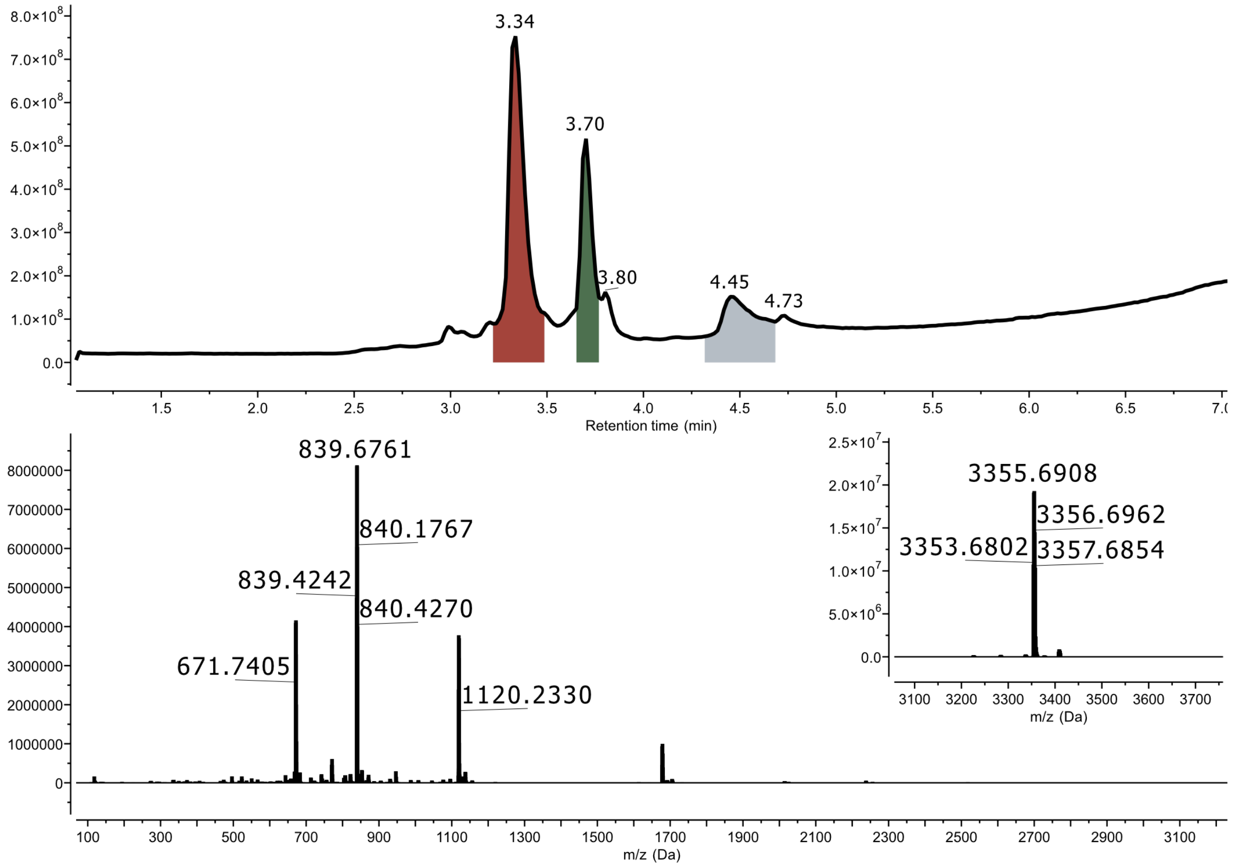

SI Figure 18. LC-HR-ESI-QTOF Profile of reaction mixture after incubation (t = 40 h). (a) TIC showing purified GLP-1[7–37]-ArgTag (red); Rt 3.34 min. Desired peptide GLP-1[7–37] (green); Rt 3.70 min. Enzyme peak at Rt 4.45 min (grey). (b) ESI-TOF spectrum found within Rt 3.70 min (insert deconvoluted masses). Monoisotopic mass (ESI+) $C_{151}H_{228}N_{40}O_{47}$ 3353.6681, found 3353.6802. LCMS Gradient B (**Section 2.8**).

Conditions 2: 1:5 molar ratio of enzyme-to-peptide, 8 U/mL enzyme concentration

LC-QTOF of reaction mixture before incubation (t = 0 min)

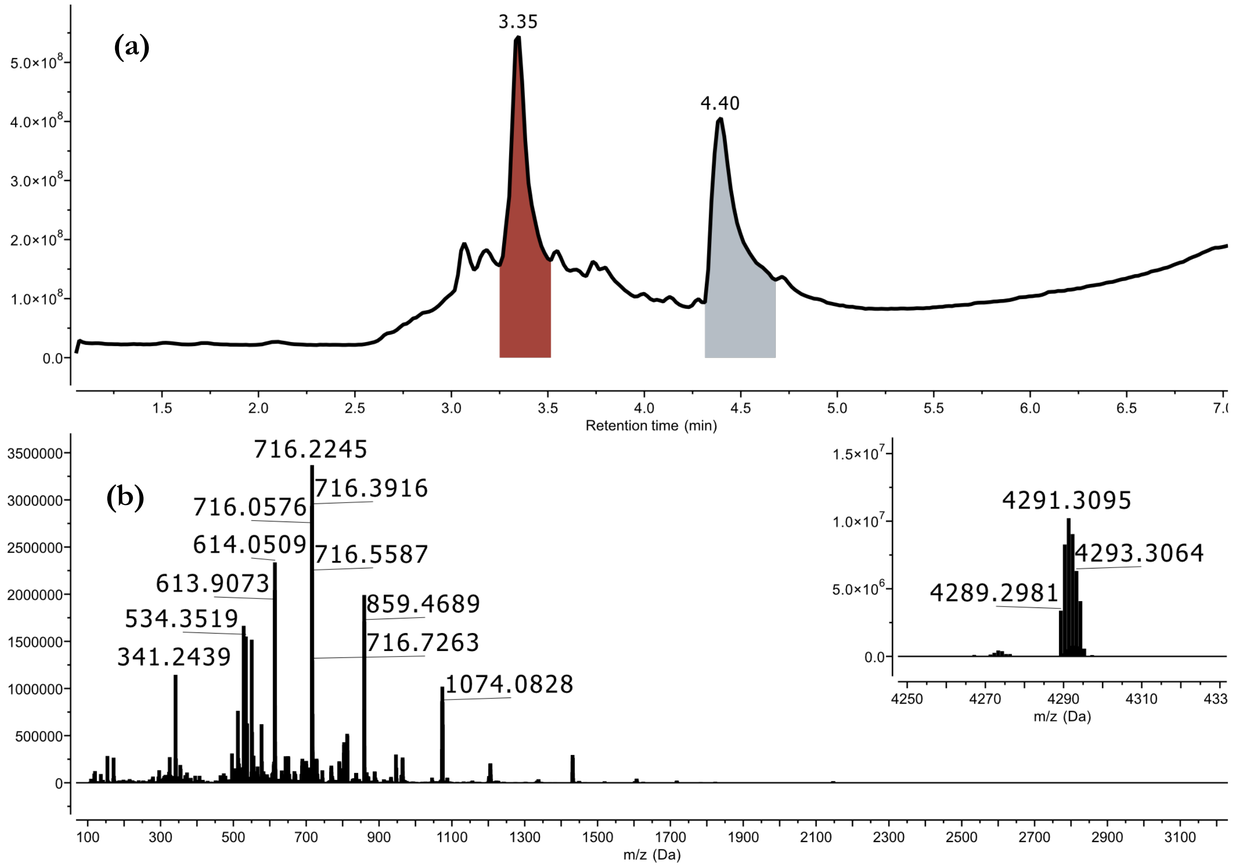

SI Figure 19. LC-HR-ESI-QTOF Profile of reaction mixture before incubation (t = 0 min). (a) TIC showing purified GLP-1[7–37]-ArgTag (red); Rt 3.35 min. Enzyme peak at Rt 4.40 min (grey). (b) ESI-TOF spectrum found within Rt 3.35 min (insert deconvoluted masses). Monoisotopic mass (ESI+) $C_{187}H_{301}N_{65}O_{52}$ 4289.2907, found 4289.2981. LCMS Gradient B (**Section 2.8**).

LC-QTOF of reaction mixture after incubation (t = 60 min)

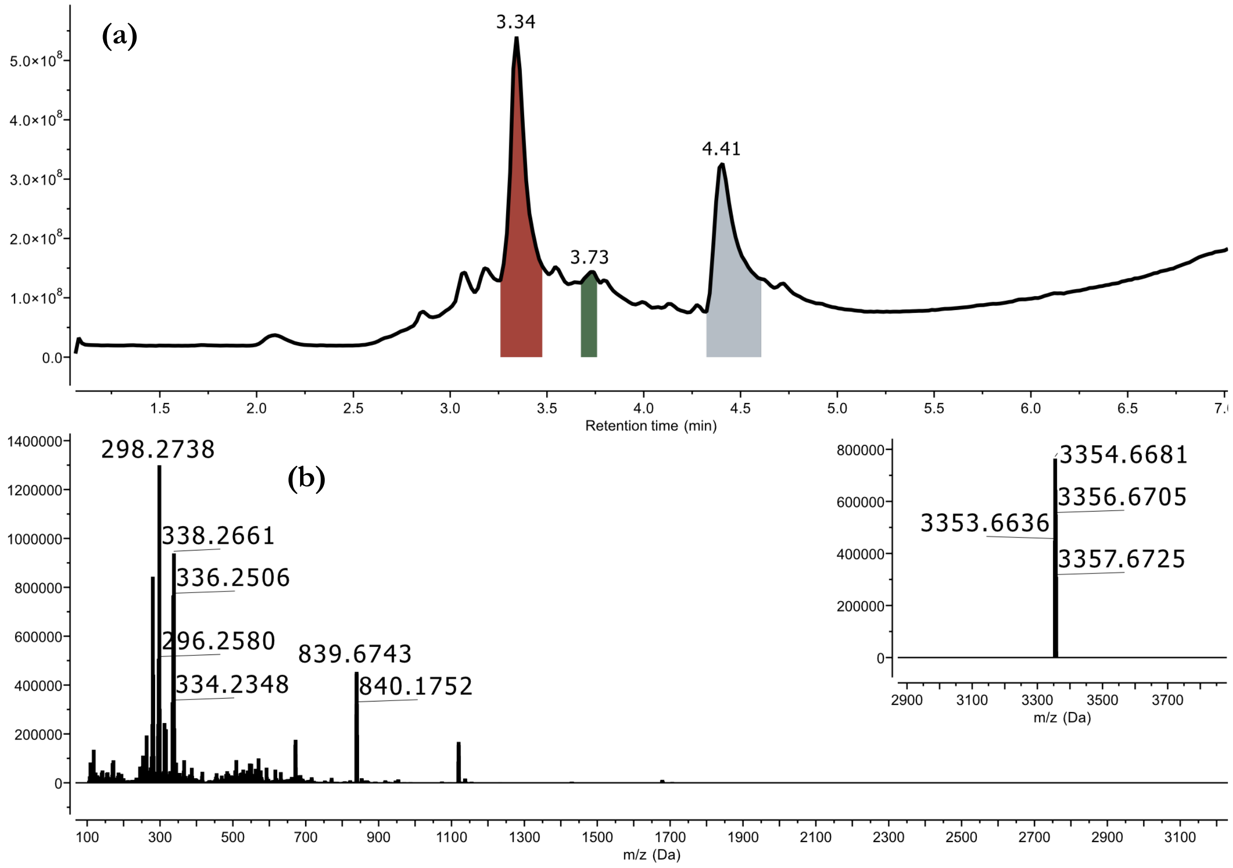

SI Figure 20. LC-HR-ESI-QTOF Profile of reaction mixture before incubation (t = 60 min). (a) TIC showing purified GLP-1[7–37]-ArgTag (red); Rt 3.35 min. Desired peptide GLP-1[7–37] (green); Rt 3.73 min. Enzyme peak at Rt 4.41 min (grey). (b) ESI-TOF spectrum found within Rt 3.73 min (insert deconvoluted masses). Monoisotopic mass (ESI+) $C_{151}H_{228}N_{40}O_{47}$ 3353.6681, found 3353.6636. LCMS Gradient B (**Section 2.8**).

LC-QTOF of reaction mixture after incubation (t = 24 h)

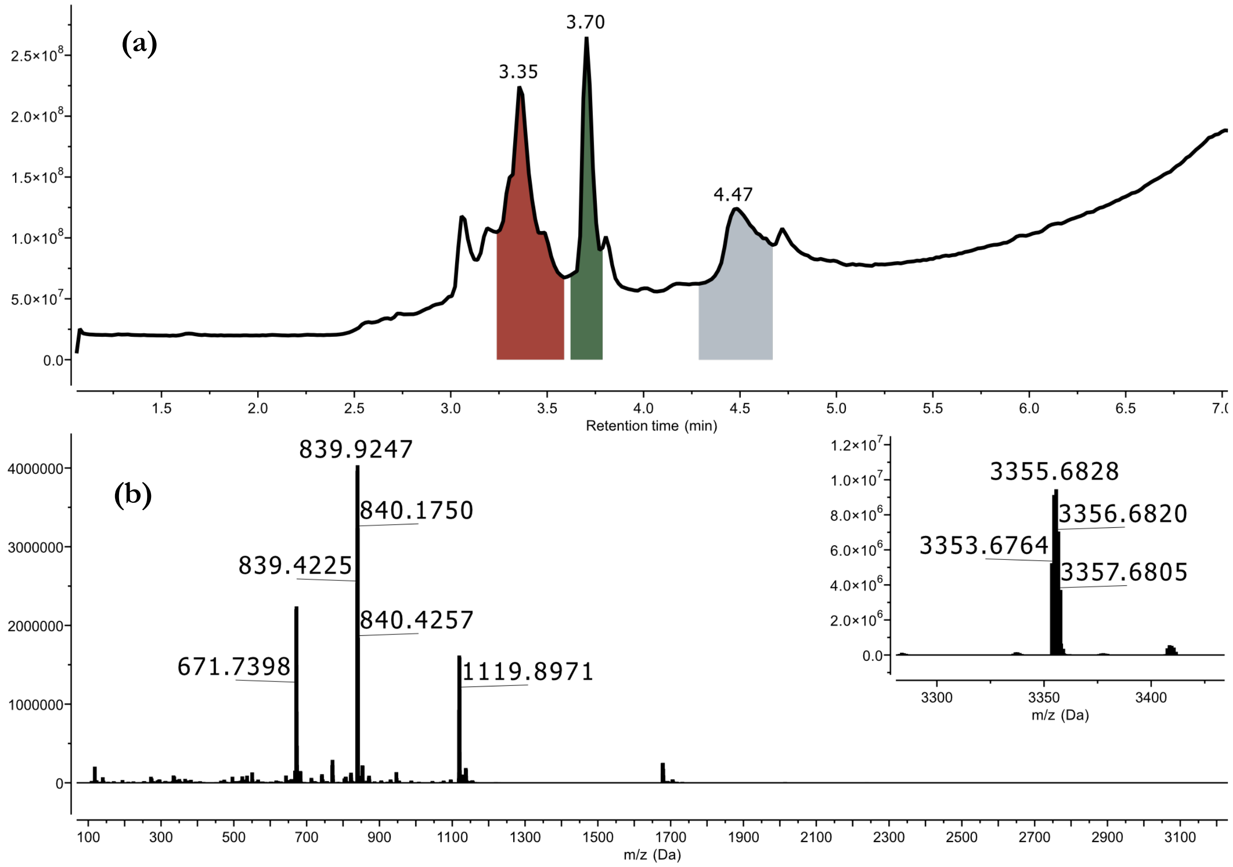

SI Figure 21. LC-HR-ESI-QTOF Profile of reaction mixture before incubation (t = 24 h). (a) TIC showing purified GLP-1[7–37]-ArgTag (red); Rt 3.35 min. Desired peptide GLP-1[7–37] (green); Rt 3.70 min. Enzyme peak at Rt 4.47 min (grey). (b) ESI-TOF spectrum found within Rt 3.70 min (insert: deconvoluted masses). Monoisotopic mass (ESI+) $C_{151}H_{228}N_{40}O_{47}$ 3353.6681, found 3353.6764. LCMS Gradient B (**Section 2.8**).

LC-QTOF of reaction mixture after incubation (t = 40 h)

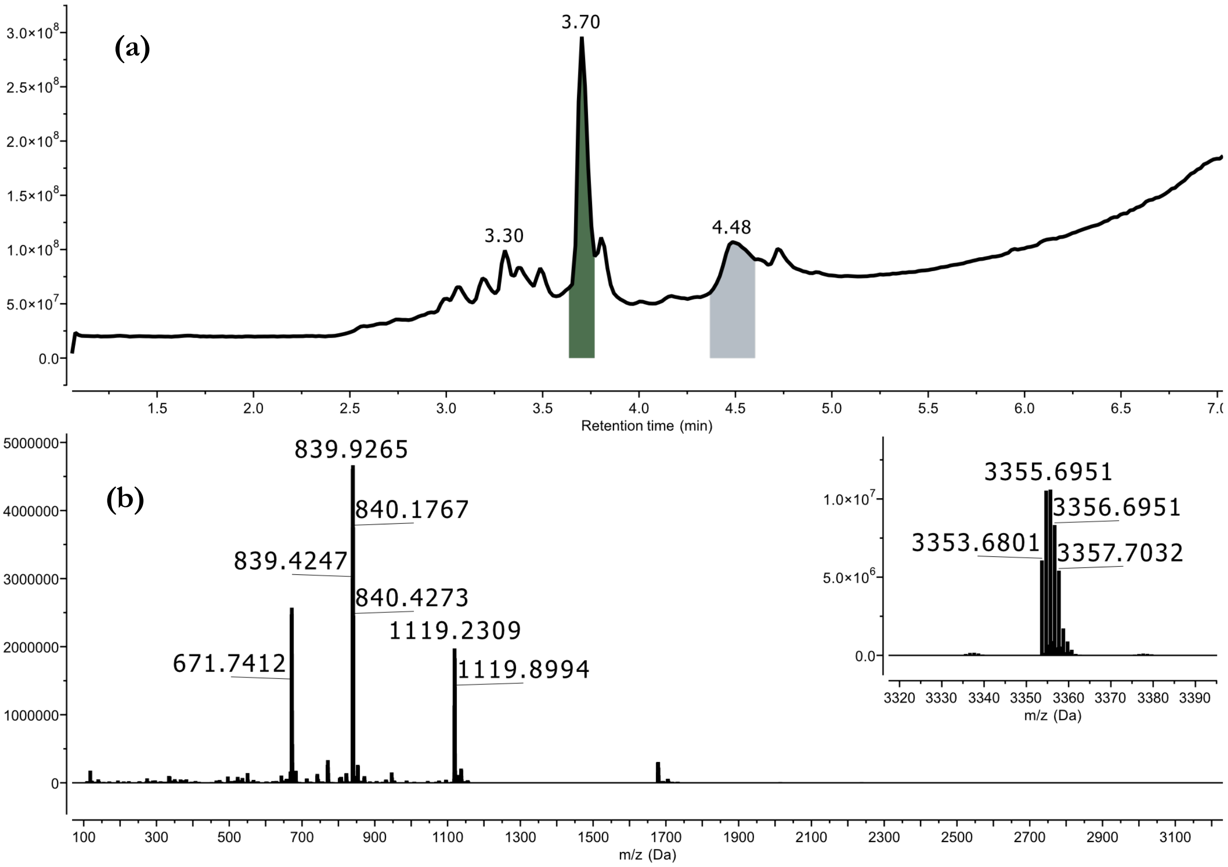

SI Figure 22. LC-HR-ESI-QTOF Profile of reaction mixture before incubation (t = 40 h). (a) TIC showing desired peptide GLP-1[7–37] (green); Rt 3.70 min. Enzyme peak at Rt 4.48 min (grey). (b) ESI-TOF spectrum found within Rt 3.70 min (insert: deconvoluted masses). Monoisotopic mass (ESI+) $C_{151}H_{228}N_{40}O_{47}$ 3353.6681, found 3353.6764. LCMS Gradient B (Section 2.8). At Rt 3.30, no starting peptide GLP-1[7–37]-ArgTag could be detected anymore.

LC-QTOF of purified GLP-1[7–37]

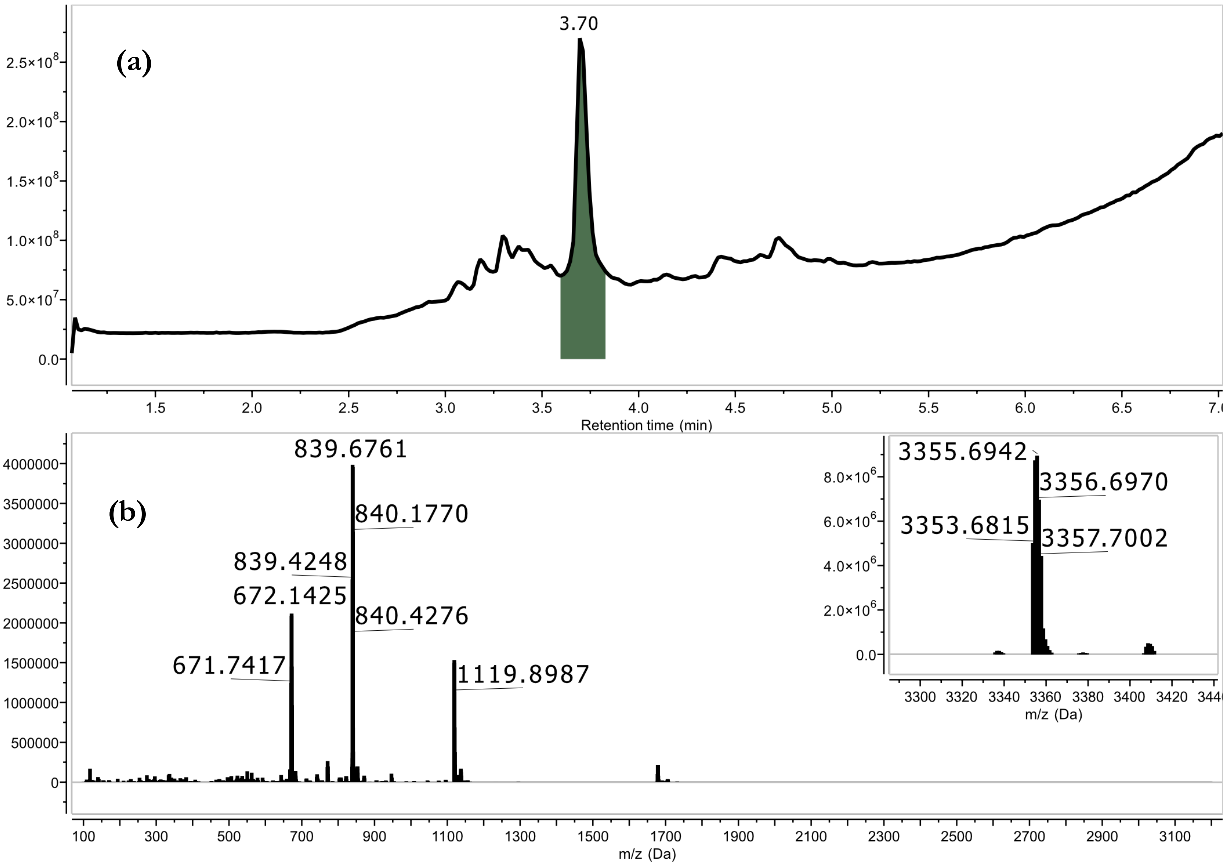

SI Figure 23. LC-HR-ESI-QTOF Profile of purified GLP-1[7–37]. (a) TIC showing desired peptide GLP-1[7–37] (green); Rt 3.70 min. (b) ESI-TOF spectrum found within Rt 3.70 min (insert: deconvoluted masses). Monoisotopic mass (ESI+) $C_{151}H_{228}N_{40}O_{47}$ 3353.6681, found 3353.6815.

Conditions 3: 1:5 molar ratio of enzyme-to-peptide, 229 U/mL enzyme concentration

LC-QTOF of reaction mixture before incubation (t = 0 min)

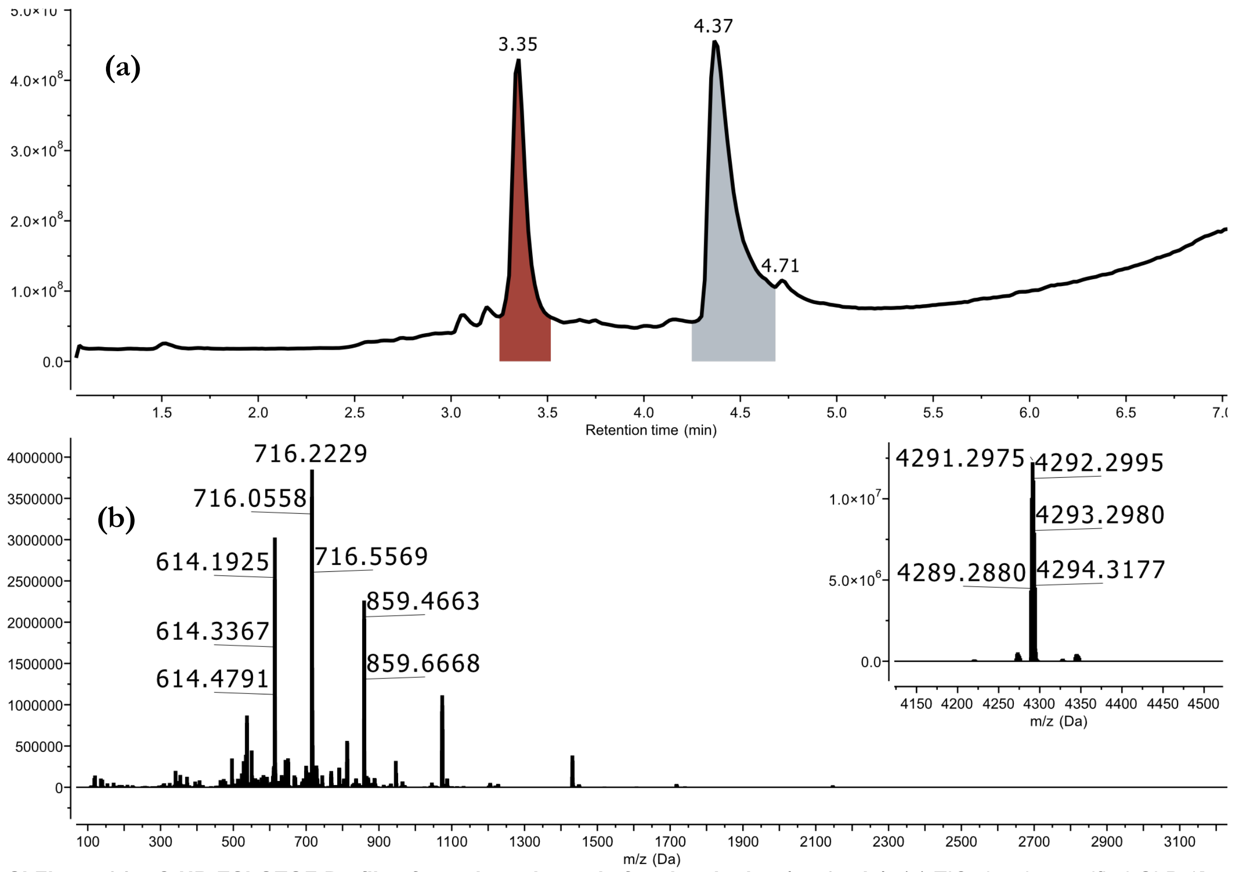

SI Figure 24. LC-HR-ESI-QTOF Profile of reaction mixture before incubation (t = 0 min). (a) TIC showing purified GLP-1[7–37]-ArgTag (red); Rt 3.35 min. Enzyme peak at Rt 4.37 min (grey). (b) ESI-TOF spectrum found within Rt 3.35 min (insert: deconvoluted masses). Monoisotopic mass (ESI+) $C_{187}H_{301}N_{65}O_{52}$ 4289.2907, found 4289.2880. LCMS Gradient B (**Section 2.8**).

LC-QTOF of reaction mixture after incubation (t = 60 min)

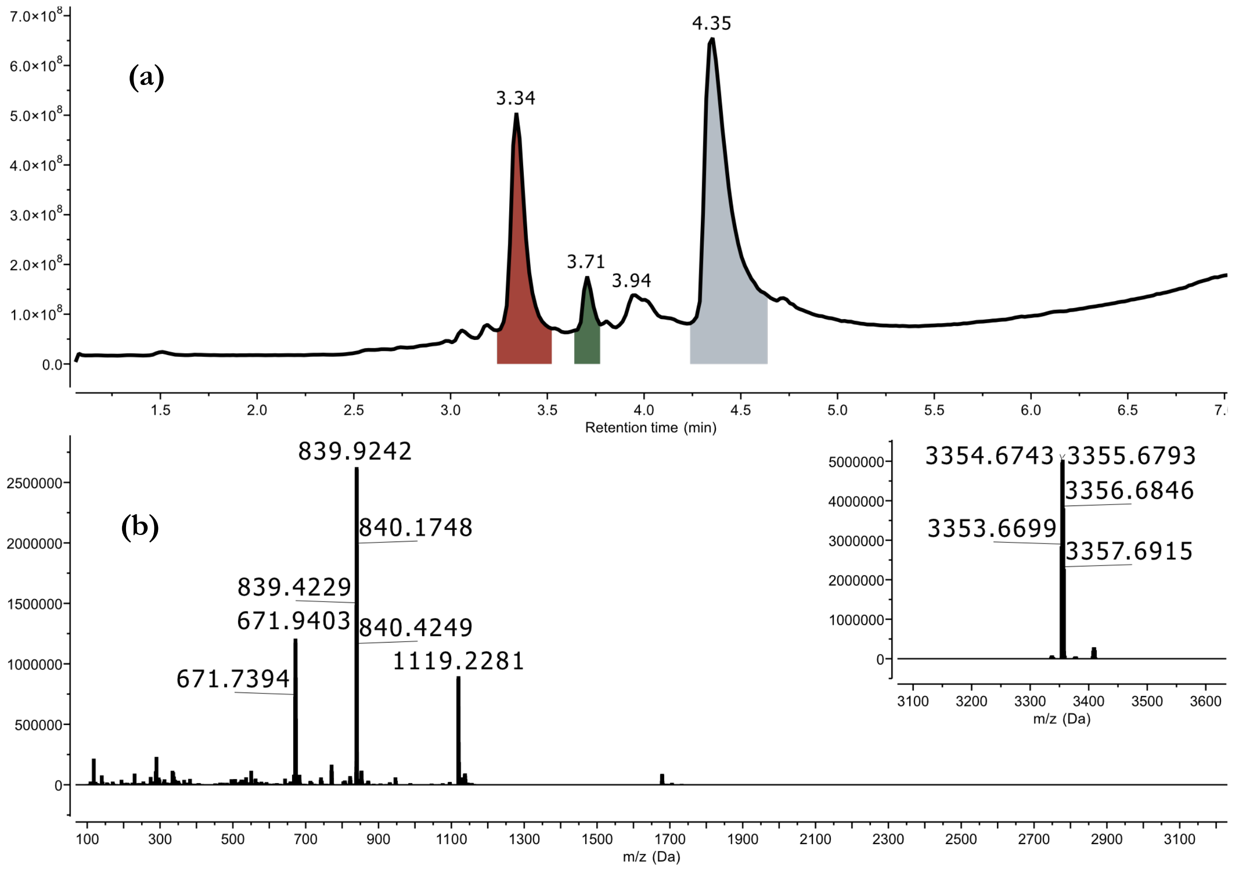

SI Figure 25. LC-HR-ESI-QTOF Profile of reaction mixture after incubation (t = 60 min). (a) TIC showing purified GLP-1[7–37]-ArgTag (red); Rt 3.34 min. Desired peptide GLP-1[7–37] (green); Rt 3.71 min. Enzyme peak at Rt 4.35 min (grey). (b) ESI-TOF spectrum found within Rt 3.71 min (insert deconvoluted masses). Monoisotopic mass (ESI+) $C_{151}H_{228}N_{40}O_{47}$ 3353.6681, found 3353.6699. LCMS Gradient B (**Section 2.8**).

LC-QTOF of reaction mixture after incubation (t = 3 h)

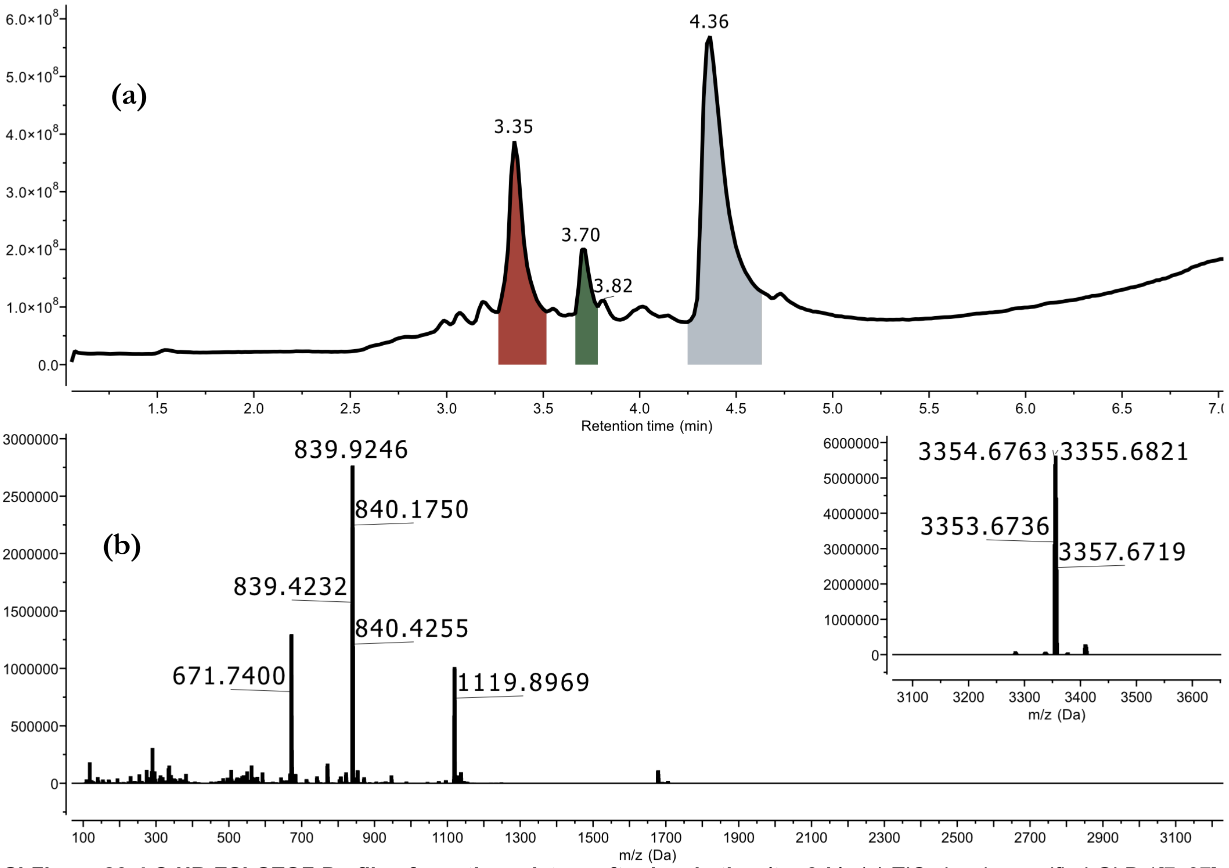

SI Figure 26. LC-HR-ESI-QTOF Profile of reaction mixture after incubation (t = 3 h). (a) TIC showing purified GLP-1[7–37]-ArgTag (red); Rt 3.35 min. Desired peptide GLP-1[7–37] (green); Rt 3.70 min. Enzyme peak at Rt 4.36 min (grey). (b) ESI-TOF spectrum found within Rt 3.70 min (insert deconvoluted masses). Monoisotopic mass (ESI+) $C_{151}H_{228}N_{40}O_{47}$ 3353.6681, found 3353.6736. LCMS Gradient B (**Section 2.8**).

LC-QTOF of reaction mixture after incubation (t = 24 h)

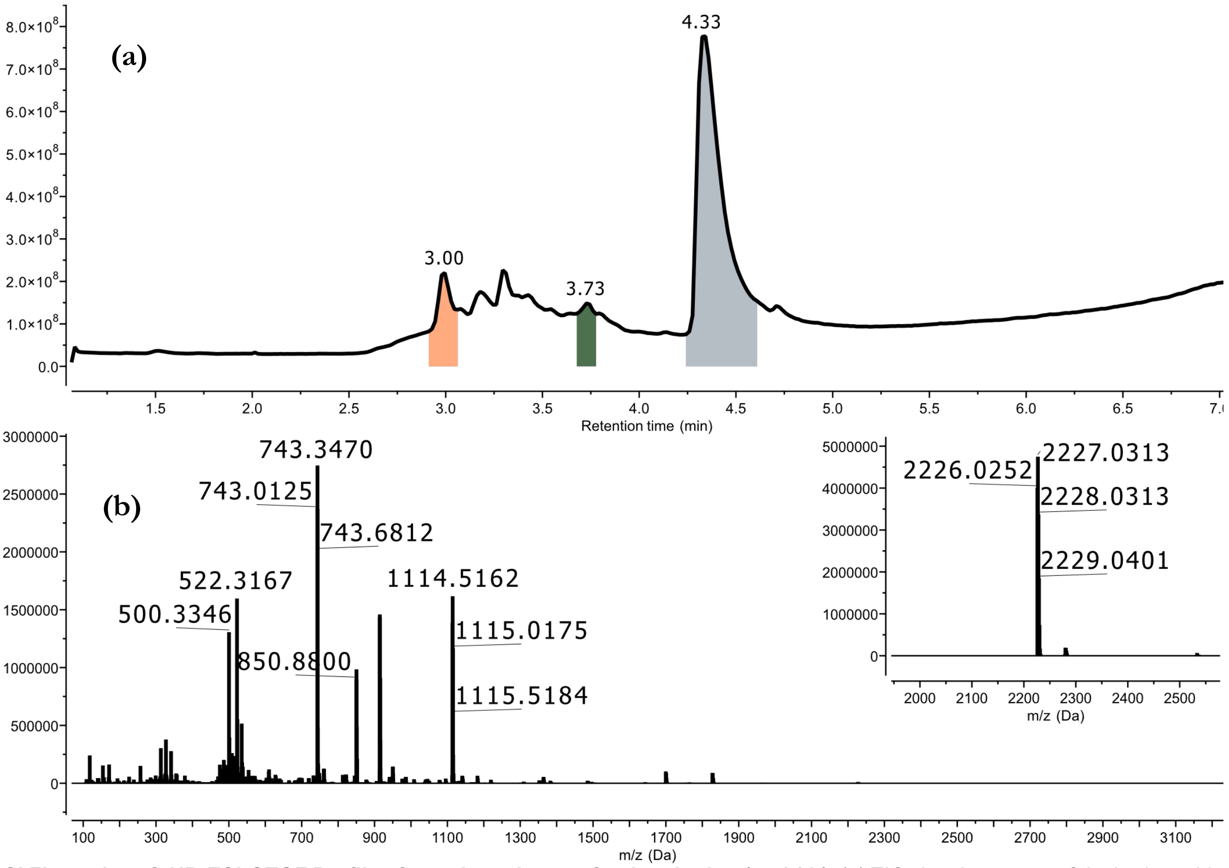

SI Figure 27. LC-HR-ESI-QTOF Profile of reaction mixture after incubation (t = 24 h). (a) TIC showing traces of desired peptide GLP-1[7–37] (green); Rt 3.73 min. Enzyme peak at Rt 4.33 min (grey). Degraded product (orange, overdigestion, structure SI Figure 28); Rt 3.00 min (b) ESI-TOF spectrum found within Rt 3.00 min (insert deconvoluted masses). Monoisotopic mass (ESI+) $C_{95}H_{143}N_{25}O_{37}$ 2226.0077, found 2226.0252. LCMS Gradient B (**Section 2.8**).

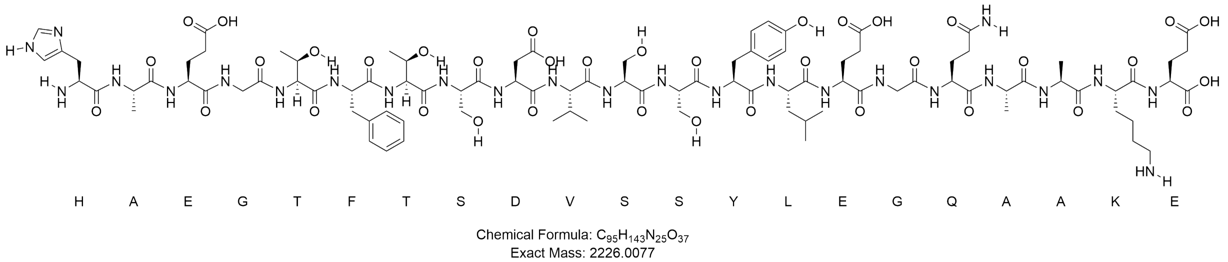

SI Figure 28. Proposed structure of overdigested product. The detected mass corresponds to the sequence GLP-1[7–27].

3.2.3 MYC[123–143]-ArgTag

```
           10         20          30
H2N-FIKNIIIQDC MWSGFSAAAK RRRRRR      -CONH2        (26 AA)
```

The peptide MYC[123–143]-ArgTag was synthesized on commercially available Rink amide NovaPEG resin (0.20 mmol/g, 149.6 mg, 30 µmol) using the standard AFPS protocol (**Section 2.2.2**) (**SI Figure 29**). Total synthesis time to afford resin-bound MYC[123–143]-ArgTag was approximately 1.5 h. Cleavage of the peptidyl-resin (50 mg, approx. 10 µmol) according to the cleavage protocol described in **Section 2.6** afforded the crude peptide as a colorless solid (5.9 mg, mass confirmed by LC-HR-ESI-QTOF [**SI Figure 30**], 67% purity by UHPLC [**SI Figure 31**]).

Enzymatic removal of the ArgTag from purified MYC[123–143]-ArgTag (according to method described in **Section 2.9**) was performed by using recombinant Carboxypeptidase B (254 U/mg specific activity) in a 1:5 molar ratio compared to MYC[123–143]-ArgTag in TrisHCl buffer (pH 8.0 at 23 °C).[3] The final enzyme concentration was 0.9 µM (8 U/mL), the final peptide concentration 4.6 µM. The reaction solution was incubated at 37 °C for 90 h and then stopped even though ArgTag was still removed, however, very slowly (**SI Figures 32 – 34**).

UV-Vis synthesis trace

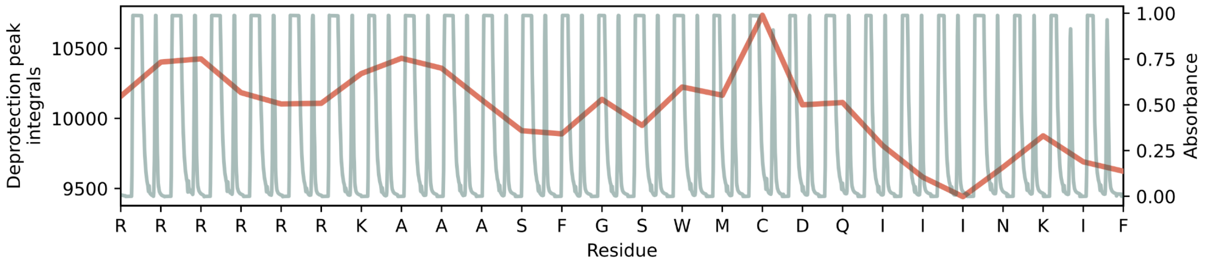

SI Figure 29. UV trace (λ = 310 nm) from AFPS of MYC[123–143]-ArgTag (green) and deprotection peak integrals (red). Note: UV chromatograms are plotted against time and are not directly aligned with the sequence labels on the x-axis. Only the integrals of the deprotection peaks are aligned with the corresponding amino acid positions.

LC-QTOF of crude MYC[123–143]-ArgTag

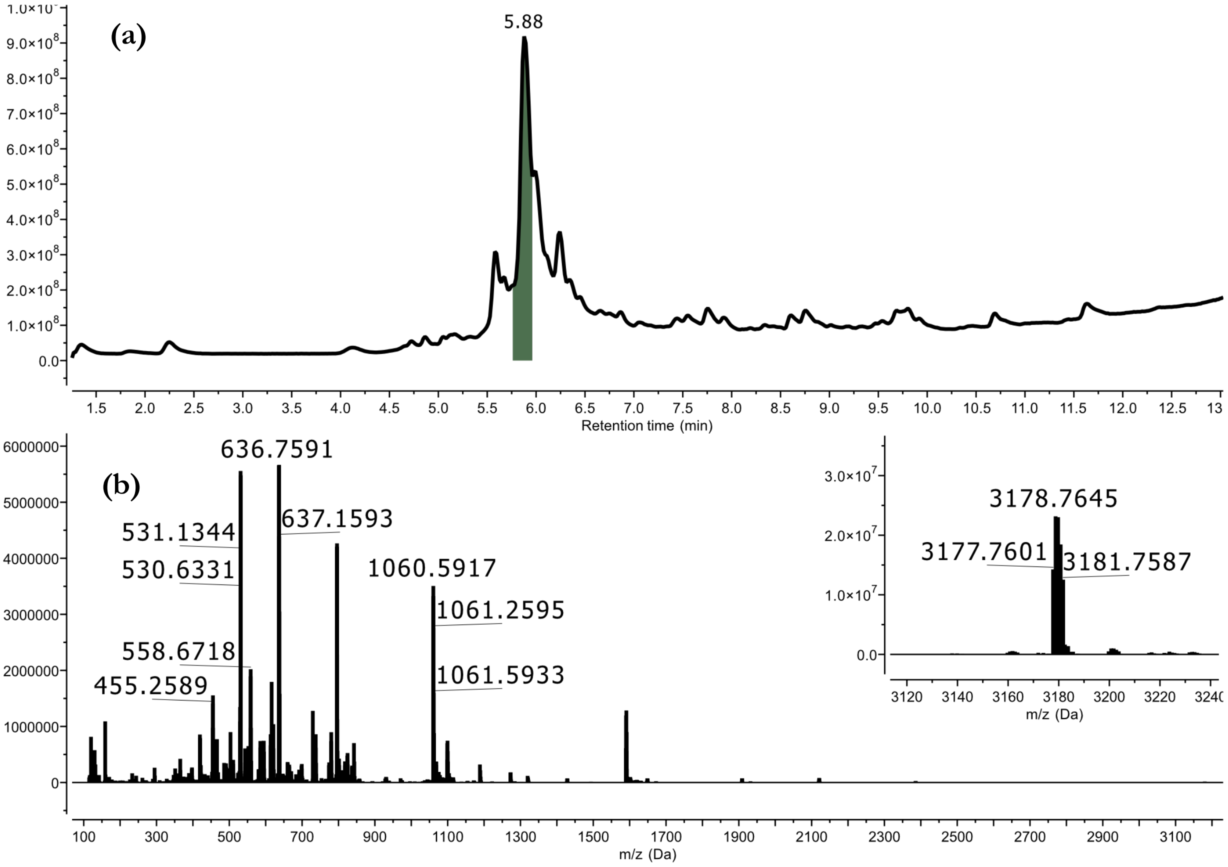

SI Figure 30. LC-HR-ESI-QTOF Profile of crude MYC[123–143] bearing the ArgTag. (a) TIC chromatogram of MYC[123–43]-ArgTag; Rt 5.88 min. (b) ESI-TOF spectrum found within Rt 5.88 min (insert: deconvoluted masses). Monoisotopic mass (ESI+) calcd. for $C_{139}H_{232}N_{50}O_{32}S_2$ 3177.7505, found 3177.7601. LCMS Gradient A (**Section 2.8**).

UHPLC of crude MYC[123–143]-ArgTag

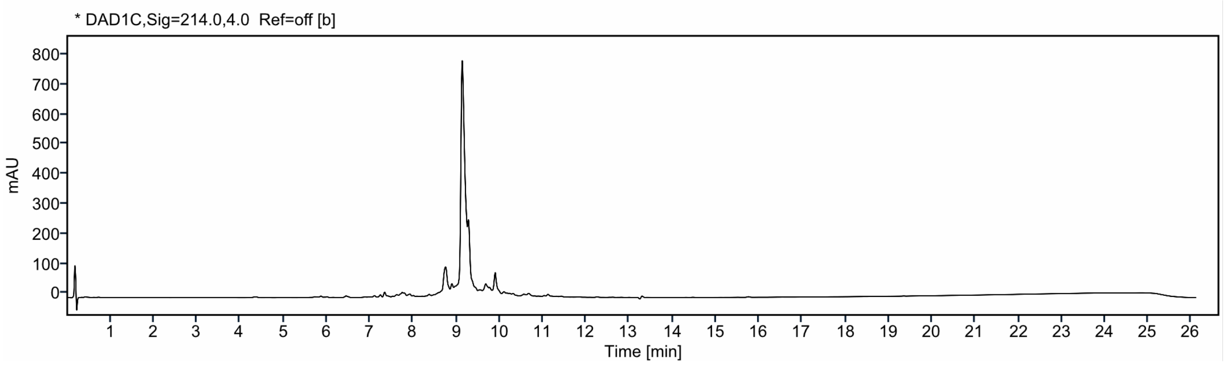

SI Figure 31. UHPLC profile of crude MYC[123–143] bearing ArgTag. Rt 9.14 min (Agilent Zorbax 300SB-C18 RRHD column, 1.8 µm, 2.1 × 50 mm, 5–95% MeCN over 20 min, ca. 4.5%B/min), 67% purity based on Area Under Curve (AUC) at λ = 214 nm.

LC-QTOF of reaction mixture after incubation (t = 1 h)

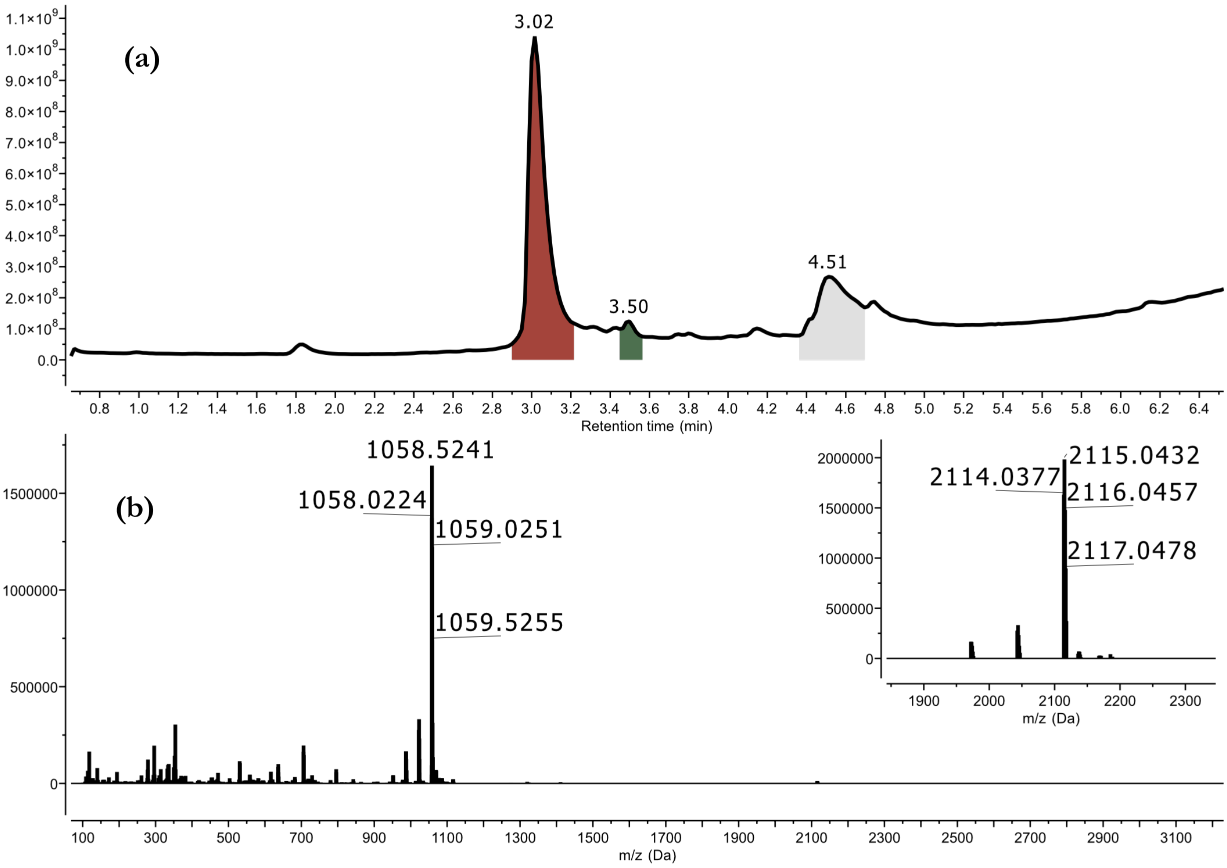

SI Figure 32. LC-HR-ESI-QTOF Profile of reaction mixture after incubation (t = 1 h). (a) TIC showing starting peptide MYC[123–143]-ArgTag (red), desired product (green) and enzyme (grey). (b) ESI-TOF spectrum found within Rt 3.50 min (insert: deconvoluted masses). Monoisotopic mass (ESI+) calcd. for $C_{97}H_{141}N_{23}O_{26}S_2$ 2114.0329, found 2114.0377 (corresponds to linear MYC[123–142]). LCMS Gradient B (**Section 2.8**).

LC-QTOF of reaction mixture after incubation (t = 24 h)

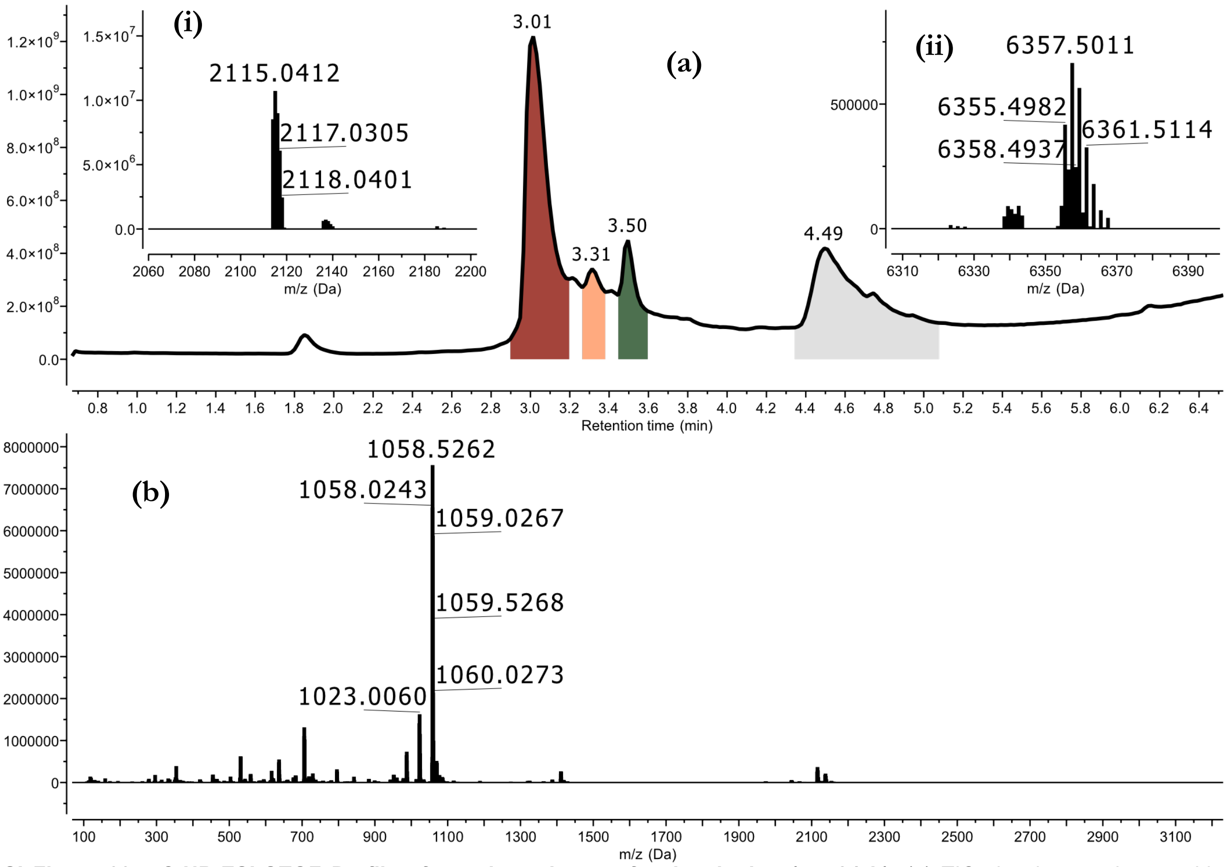

SI Figure 33. LC-HR-ESI-QTOF Profile of reaction mixture after incubation (t = 24 h). (a) TIC showing starting peptide MYC[123–143]-ArgTag (red), starting material dimer formed through disulfide bond (orange), desired product (green) and enzyme (grey). (b) ESI-TOF spectrum found within Rt 3.50 min. Insert (i): deconvoluted masses; Monoisotopic mass (ESI+) calcd. for $C_{97}H_{141}N_{23}O_{26}S_2$ 2114.0329, found 2114.0377 (corresponds to linear MYC[123–142]). Insert (ii): deconvoluted masses of ESI-TOF spectrum found within Rt 3.31 min. Monoisotopic mass (ESI+) calcd. for $C_{278}H_{462}N_{100}O_{64}S_4$ 6353.4854, found 6353.4235 (corresponds to dimer of starting peptide MYC[123–143]). LCMS Gradient B (**Section 2.8**).

LC-QTOF of reaction mixture after incubation (t = 90 h)

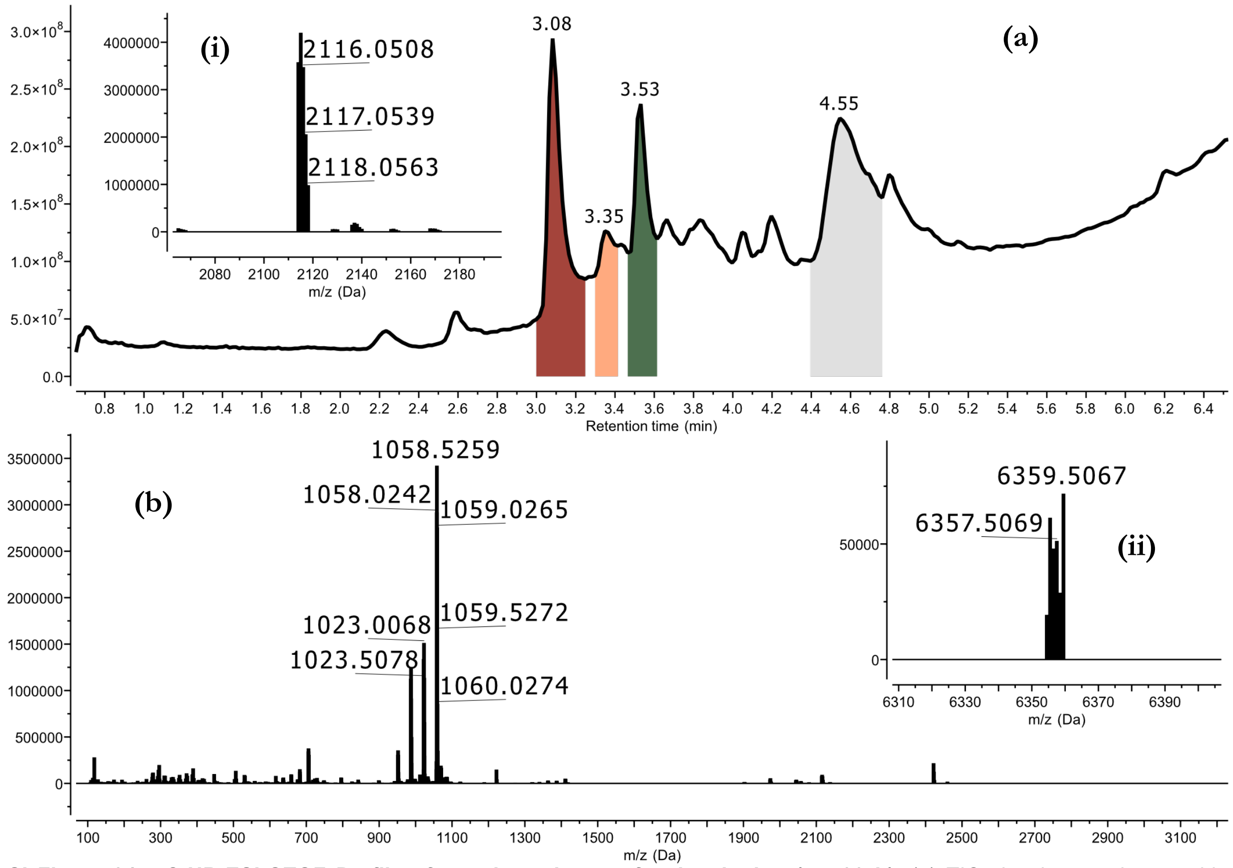

SI Figure 34. LC-HR-ESI-QTOF Profile of reaction mixture after incubation (t = 90 h). (a) TIC showing starting peptide MYC[123–143]-ArgTag (red), starting material dimer formed through disulfide bond (orange), desired product (green) and enzyme (grey). (b) ESI-TOF spectrum found within Rt 3.53 min. Insert (i): deconvoluted masses; Monoisotopic mass (ESI+) calcd. for $C_{97}H_{141}N_{23}O_{26}S_2$ 2114.0329, found 2114.0377 (corresponds to linear MYC[123–142]). Insert (ii): deconvoluted masses of ESI-TOF spectrum found within Rt 3.35 min. Monoisotopic mass (ESI+) calcd. for $C_{278}H_{462}N_{100}O_{64}S_4$ 6353.4854, found 6353.4235 (corresponds to dimer of starting peptide MYC[123–143]). LCMS Gradient B (**Section 2.8**).

3.2.4 Barstar[75–90]-ArgTag

The synthesis of the peptide Barstar[75–90]-ArgTag used for this enzymatic ArgTag removal experiment is described in **Section 4.14**. Enzymatic removal of the ArgTag from purified Barstar[75–90]-ArgTag (according to method described in **Section 2.9**) was performed by using recombinant Carboxypeptidase B (254 U/mg specific activity) in a 1:5 molar ratio compared to Barstar[75–90]-ArgTag in TrisHCl buffer (pH 8.0 at 23 °C).[3] The final enzyme concentration was 0.9 μM (8 U/mL), the final peptide concentration 4.6 μM. The reaction solution was incubated at 37 °C for 47 h until no more starting peptide Barstar[75–90]-ArgTag was detected (**SI Figures 35 – 37**).

LC-QTOF of reaction mixture before incubation (t = 0 h)

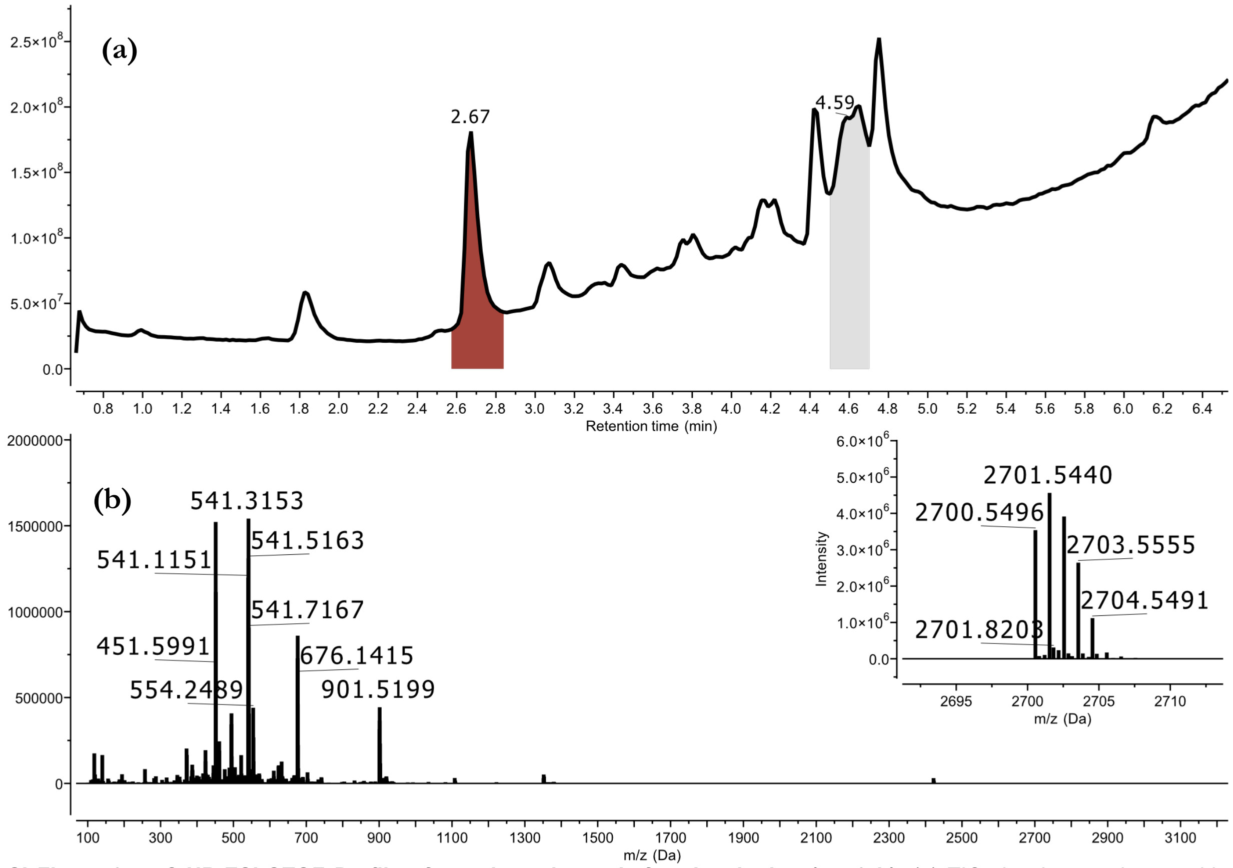

SI Figure 35. LC-HR-ESI-QTOF Profile of reaction mixture before incubation (t = 0 h). (a) TIC showing starting peptide Barstar[75–90]-ArgTag (red).(b) ESI-TOF spectrum found within Rt 2.67 min (insert: deconvoluted masses). Monoisotopic mass (ESI+) calcd. for $C_{113}H_{201}N_{45}O_{30}S$ 2700.5307, found 2700.5496. LCMS Gradient B (**Section 2.8**).

LC-QTOF of reaction mixture after incubation (t = 22 h)

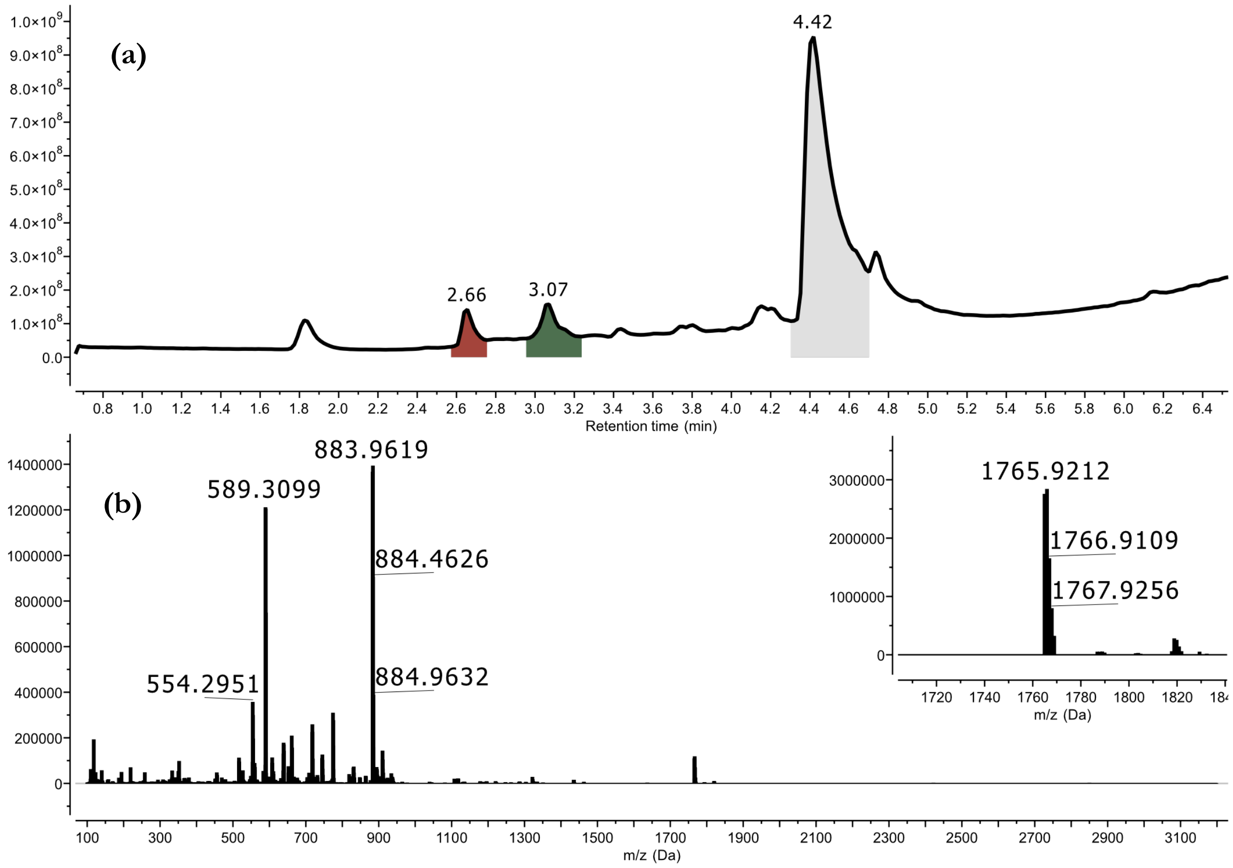

SI Figure 36. LC-HR-ESI-QTOF Profile of reaction mixture after incubation (t = 22 h). (a) TIC showing starting peptide Barstar[75–90]-ArgTag (red), desired product (green) and enzyme (grey). (b) ESI-TOF spectrum found within Rt 3.07 min. Insert (i): deconvoluted masses; Monoisotopic mass (ESI+) calcd. for $C_{77}H_{128}N_{20}O_{25}S$ 1764.9080, found 1764.9113. LCMS Gradient B (**Section 2.8**).

LC-QTOF of reaction mixture after incubation (t = 47 h)

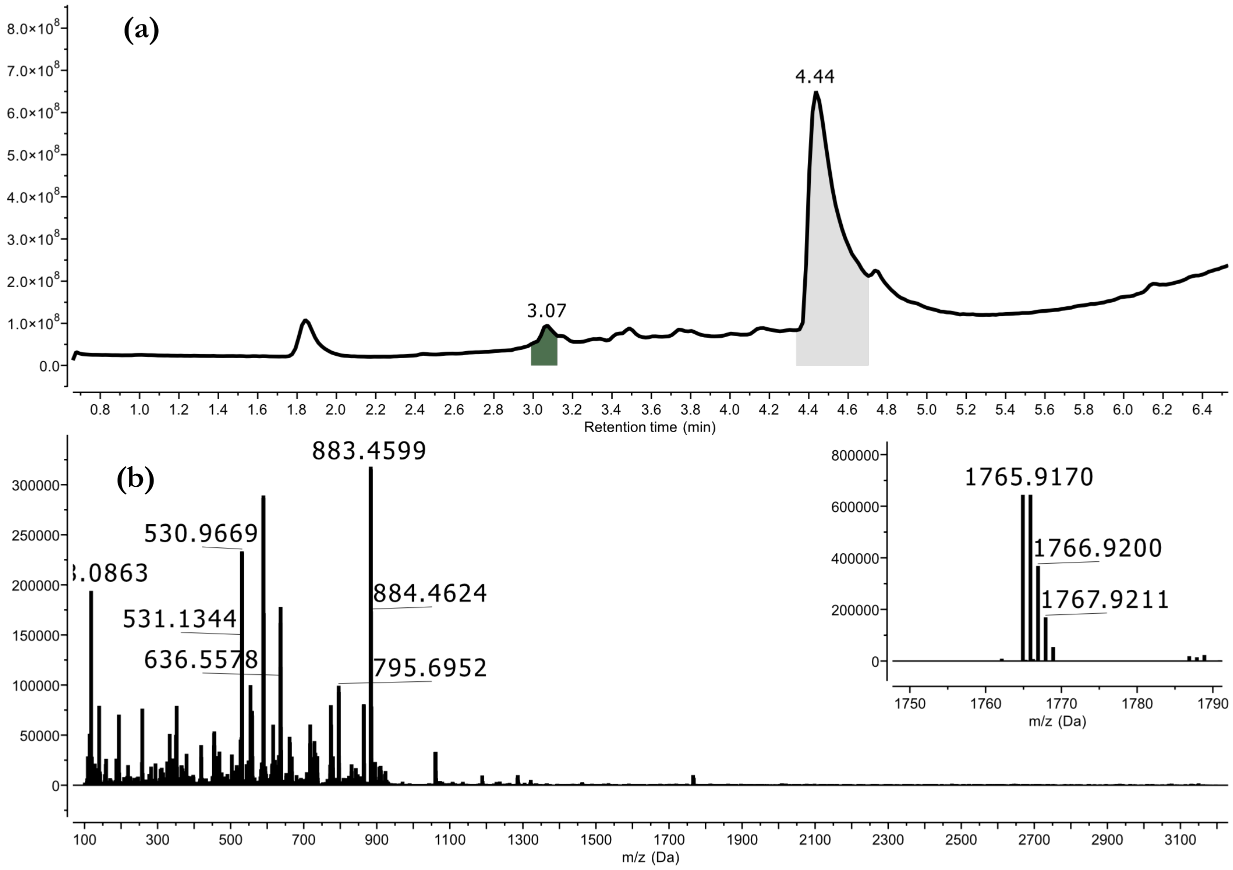

SI Figure 37. LC-HR-ESI-QTOF Profile of reaction mixture after incubation (t = 47 h). (a) TIC showing desired product (green) and enzyme (grey). (b) ESI-TOF spectrum found within Rt 3.07 min. Insert (i): deconvoluted masses; Monoisotopic mass (ESI+) calcd. for $C_{77}H_{128}N_{20}O_{25}S$ 1764.9080, found 1764.9118. LCMS Gradient B (**Section 2.8**).

3.2.5 GLP-1[7–37]-SynTag

The peptide GLP-1[7–37]-SynTag was synthesized on MeDbz-[Arg(Pbf)]$_6$ Rink Amide NovaPEG resin (163.0 mg, 41 μmol), prepared according to a reported protocol[4] and obtained using the standard AFPS protocol (**Section 2.2.2**, **SI Figure 44**). Total synthesis time to afford resin-bound GLP-1[7–37]-SynTag was approximately 3.0 h including manual batch coupling of the final Boc-His(Trt)-OH (according to **Section 2.2.4**). The resin was then washed with DMF (3 x 3 mL) and DCM (3 x 3 mL), and dried. An aliquot (5 mg, approx. 0.7 μmol) of the peptidyl-resin was cleaved according to the cleavage protocol described in **Section 2.6** and analyzed with LC-HR-ESI-QTOF (**SI Figure 39**, mass confirmed) and UHPLC (**SI Figure 40**, 60% purity). The peptidyl-resin (100 mg, approx. 14 μmol) was then acylated and cyclized according to the reported procedure.[4] The DIPEA cyclization step was repeated 15 times until the yellow color disappeared. Cleavage of the peptidyl-resin (13 mg, approx. 1.8 μmol) according to the cleavage protocol described in **Section 2.6** afforded the crude peptide as a colorless solid (0.8 mg, mass confirmed by LC-HR-ESI-QTOF [**SI Figure 41**], 43% purity by UHPLC [**SI Figure 42**]).

UV-Vis synthesis trace

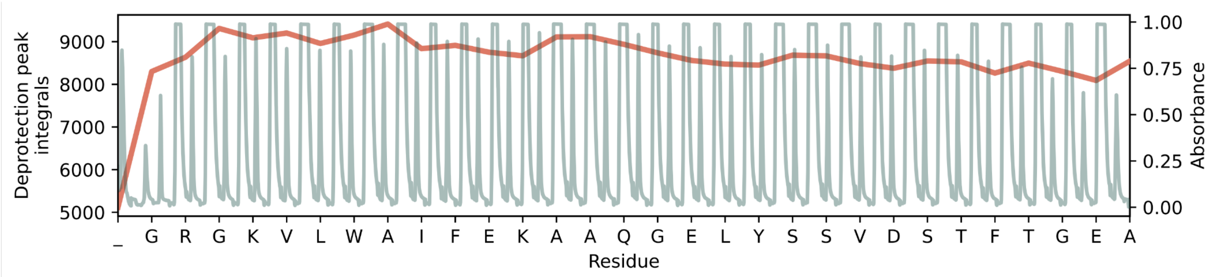

SI Figure 38. UV trace (λ = 310 nm) from AFPS of GLP-1[7–37]-SynTag (green) and deprotection peak integrals (red). Note: UV chromatograms are plotted against time and are not directly aligned with the sequence labels on the x-axis. Only the integrals of the deprotection peaks are aligned with the corresponding amino acid positions.

LC-QTOF of crude unactivated GLP-1[7–37]-SynTag

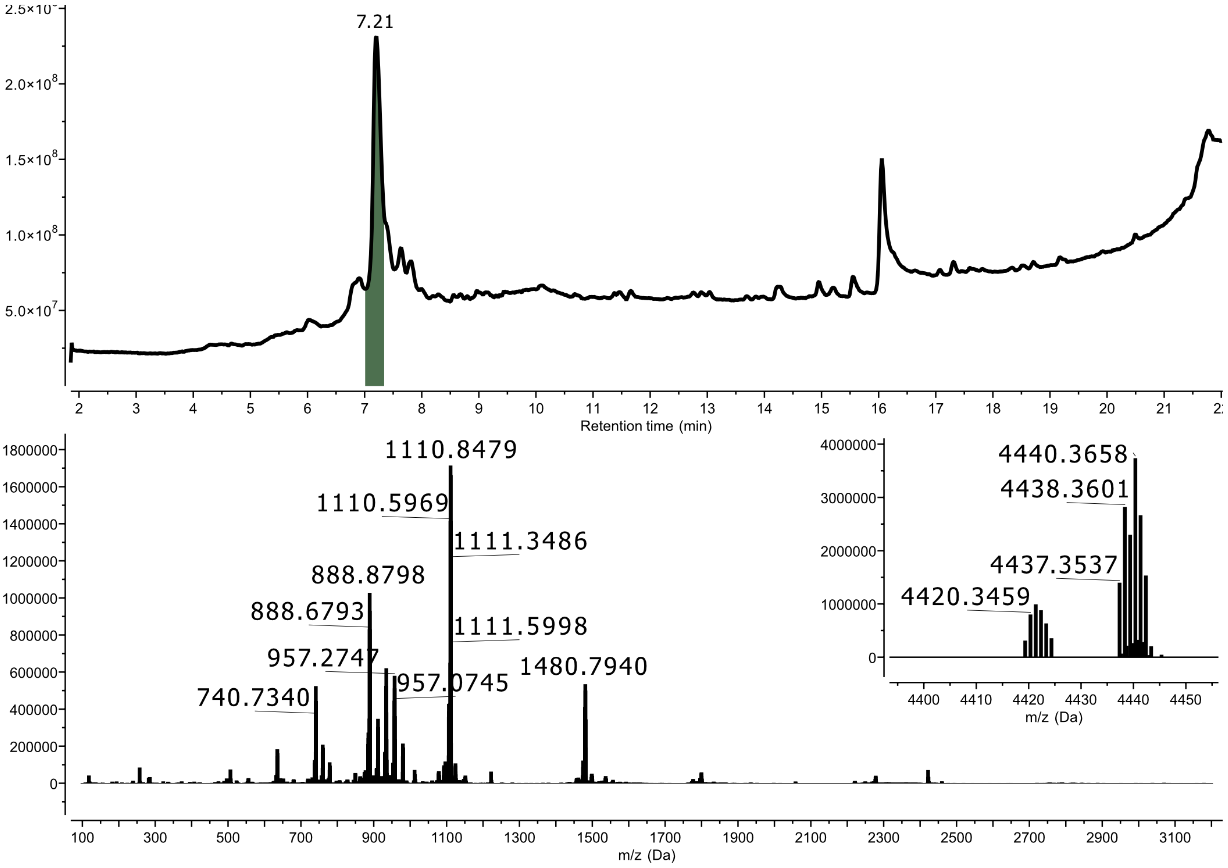

SI Figure 39. LC-HR-ESI-QTOF Profile of crude unactivated GLP-1[7–37]-SynTag. (a) TIC chromatogram of unactivated GLP-1[7–37]-SynTag; Rt 7.21 min. (b) ESI-TOF spectrum found within Rt 7.21 min (insert: deconvoluted masses). Monoisotopic mass (ESI+) calcd. for $C_{195}H_{309}N_{67}O_{53}$ 4437.3544, found 4437.3537. LCMS Gradient A (**Section 2.8**).

UHPLC of crude unactivated GLP-1[7–37]-SynTag

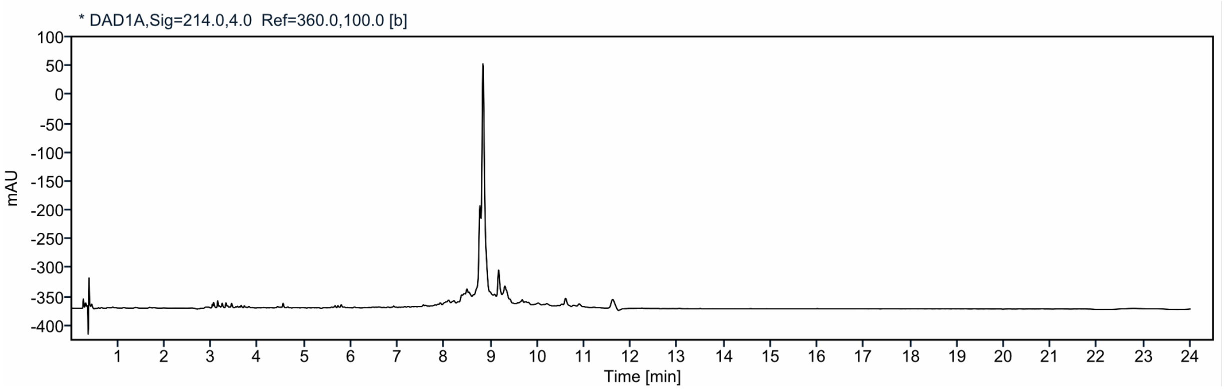

SI Figure 40. UHPLC profile of crude unactivated GLP-1[7–37]-SynTag. Rt 8.83 min (Agilent Zorbax 300SB-C18 RRHD column, 1.8 µm, 2.1 × 50 mm, 5–95% MeCN over 20 min, ca. 4.5%B/min), 60% purity based on Area Under Curve (AUC) at λ = 214 nm (**Section 2.7**).

LC-MS of crude activated GLP-1[7–37]-SynTag

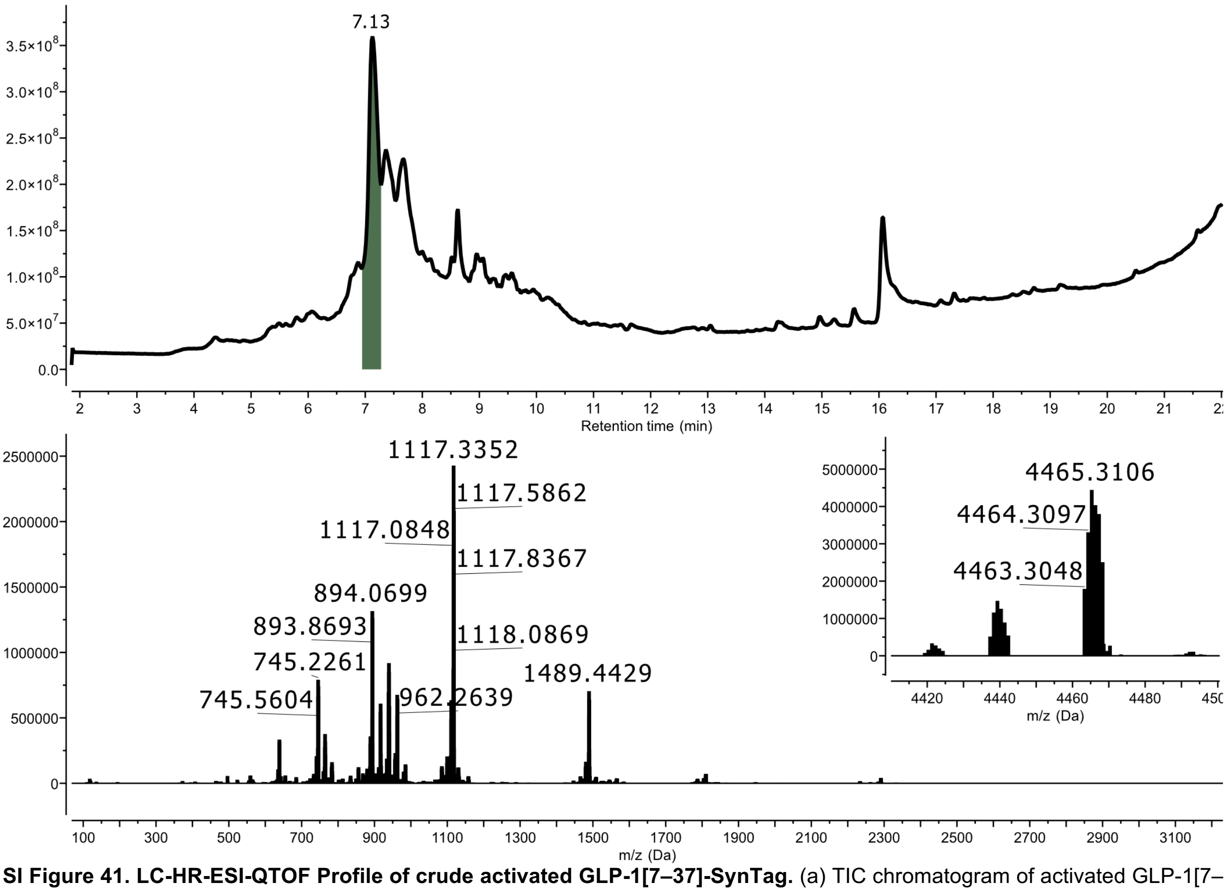

SI Figure 41. LC-HR-ESI-QTOF Profile of crude activated GLP-1[7–37]-SynTag. (a) TIC chromatogram of activated GLP-1[7–37]-SynTag; Rt 7.13 min. (b) ESI-TOF spectrum found within Rt 7.13 min (insert: deconvoluted masses). Monoisotopic mass (ESI+) calcd. for $C_{196}H_{307}N_{67}O_{54}$ 4463.3336, found 4463.3048. LCMS Gradient A (**Section 2.8**).

UHPLC of crude activated GLP-1[7–37]-SynTag

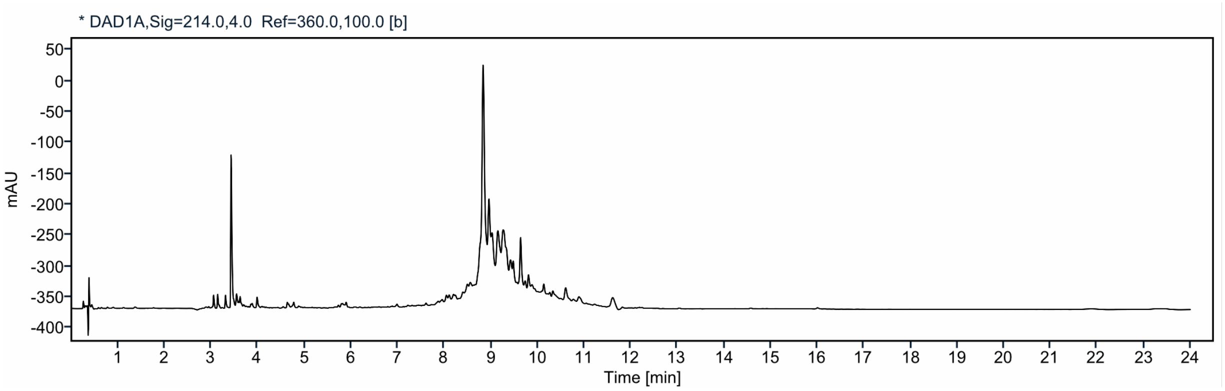

SI Figure 42. UHPLC profile of crude unactivated GLP-1[7–37]-SynTag. Rt 8.83 min (Agilent Zorbax 300SB-C18 RRHD column, 1.8 µm, 2.1 × 50 mm, 5–95% MeCN over 20 min, ca. 4.5%B/min), 43% purity based on Area Under Curve (AUC) at λ = 214 nm (**Section 2.7**). Peak at 3.43 min is not integrated and not considered for the purity calculation as it corresponds to the hydrolyzed SynTag. Peak at 9.26 min (area% of 2%) was added to the main peak at 8.83 as it corresponds to the desired native GLP-1[7–37].

Unidentifiable side-products of GLP-1[7–37]-SynTag activation

SI Figures 43 – 46 show the mass spectra of the highlighted side-product peaks with the unidentifiable masses.

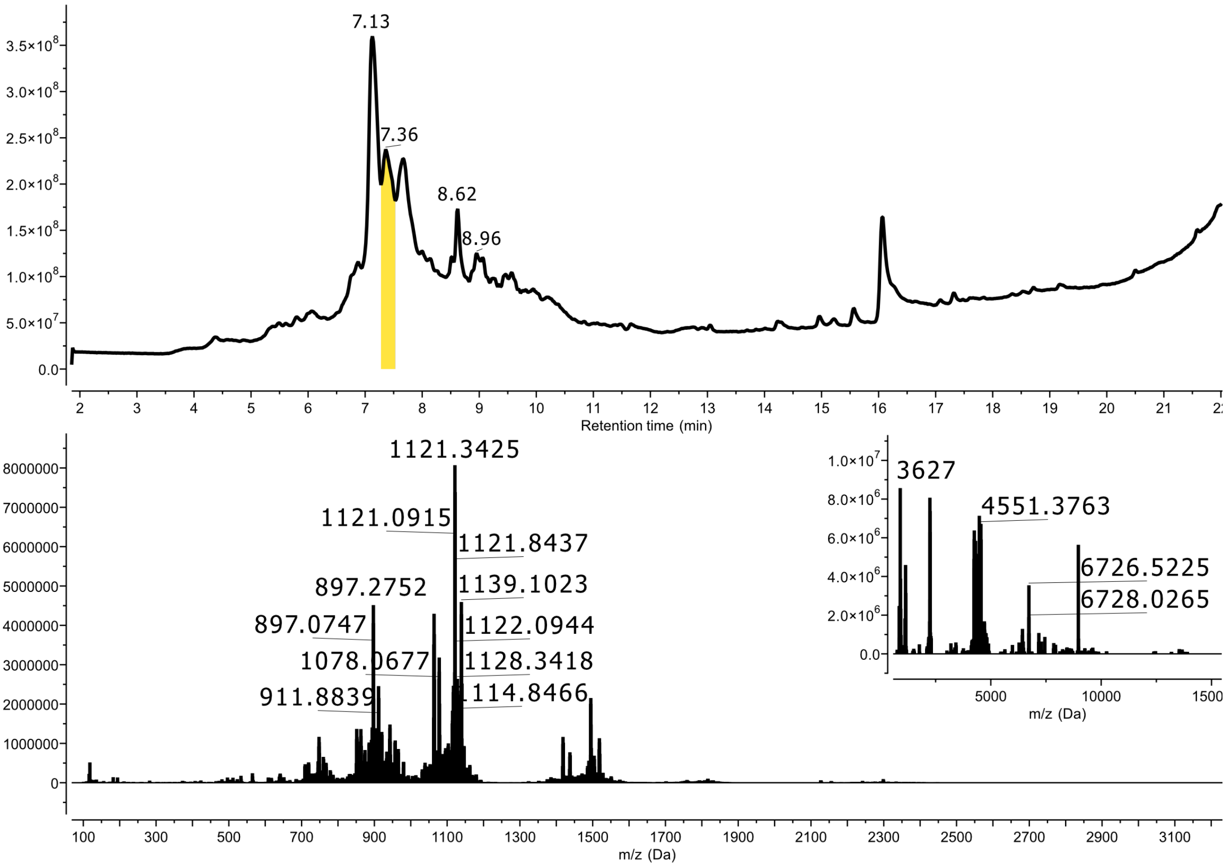

SI Figure 43. LC-HR-ESI-QTOF Profile of crude activated GLP-1[7–37]-SynTag. (a) TIC chromatogram of activated GLP-1[7–37]-SynTag; Rt 7.36 min. (b) ESI-TOF spectrum found within Rt 7.36 min (insert: deconvoluted masses). LCMS Gradient A (**Section 2.8**).

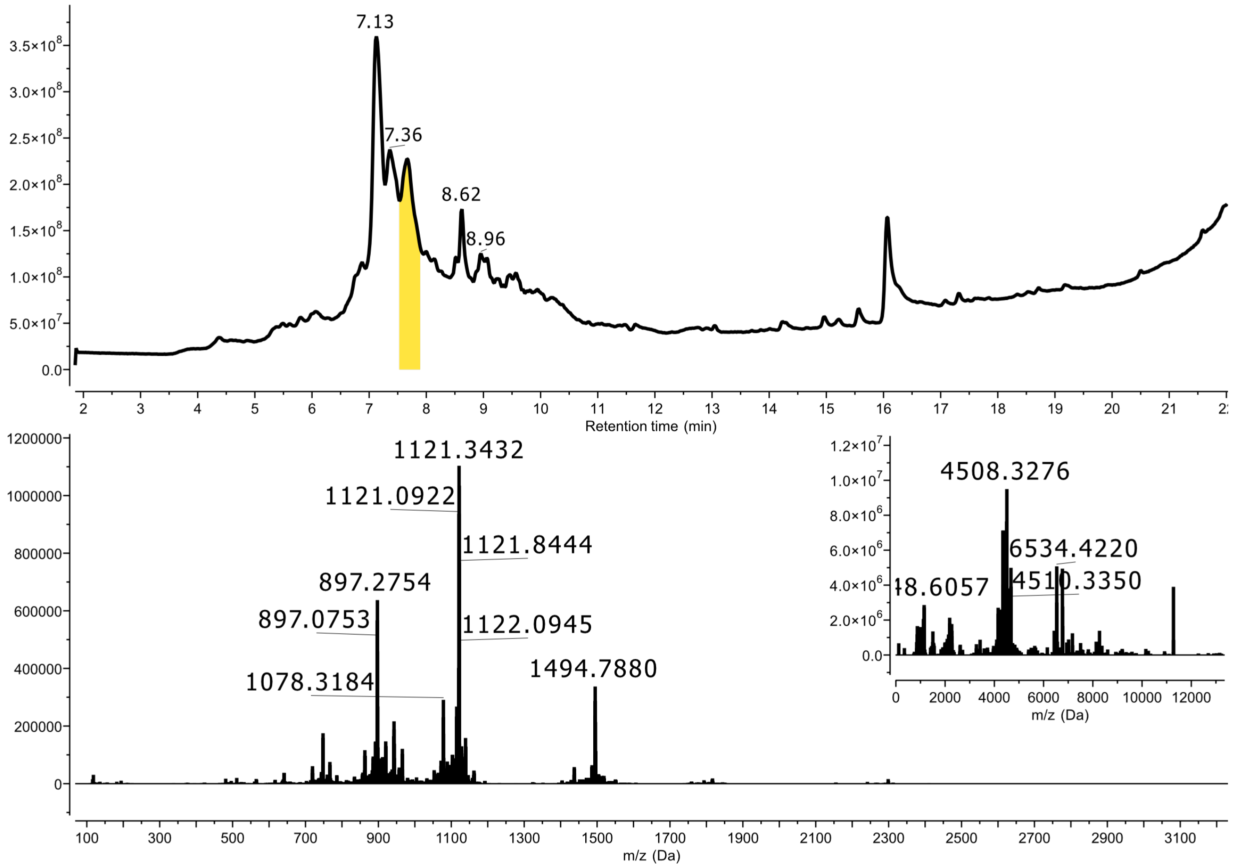

SI Figure 44. LC-HR-ESI-QTOF Profile of crude activated GLP-1[7–37]-SynTag. (a) TIC chromatogram of activated GLP-1[7–37]-SynTag; Rt 7.68 min. (b) ESI-TOF spectrum found within Rt 7.68 min (insert: deconvoluted masses). LCMS Gradient A (**Section 2.8**).

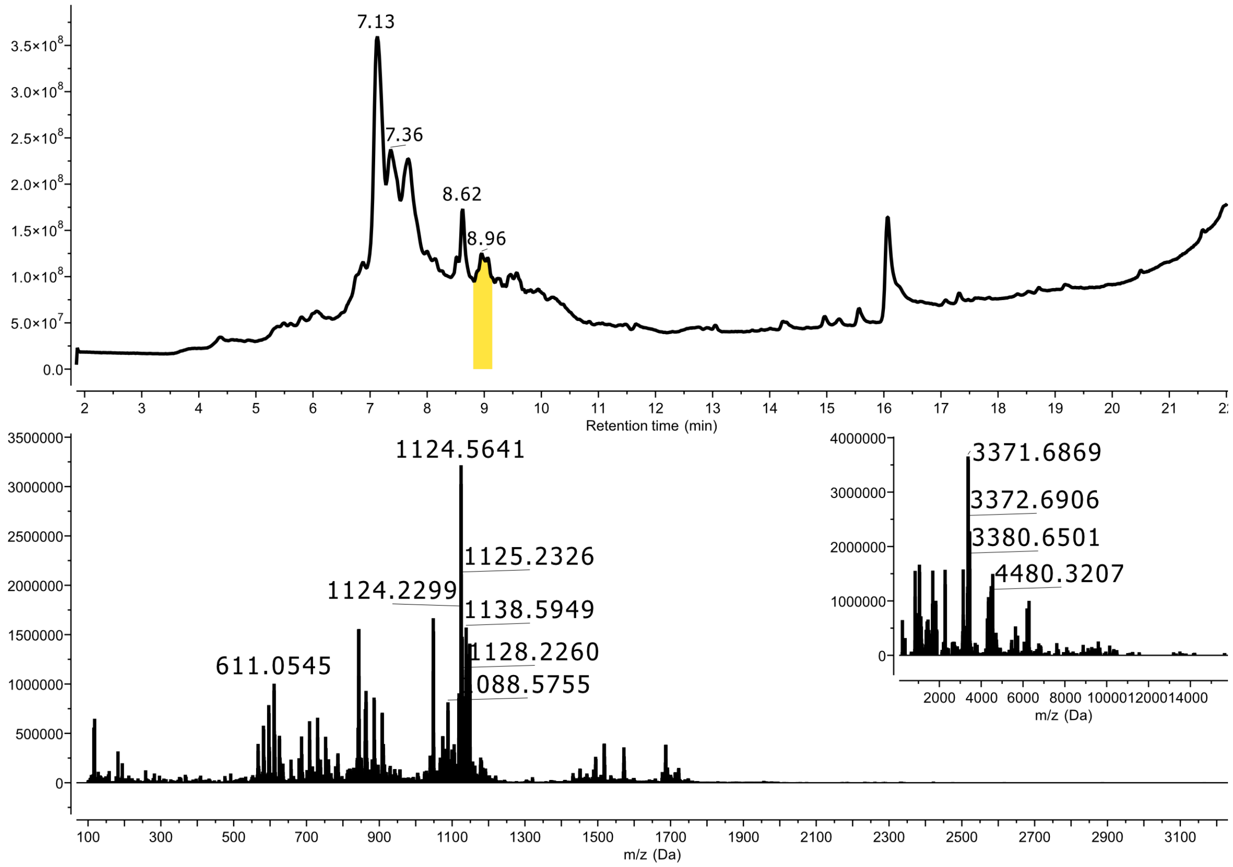

SI Figure 45. LC-HR-ESI-QTOF Profile of crude activated GLP-1[7–37]-SynTag. (a) TIC chromatogram of activated GLP-1[7–37]-SynTag; Rt 8.96 min. (b) ESI-TOF spectrum found within Rt 8.96 min (insert: deconvoluted masses). LCMS Gradient A (**Section 2.8**).

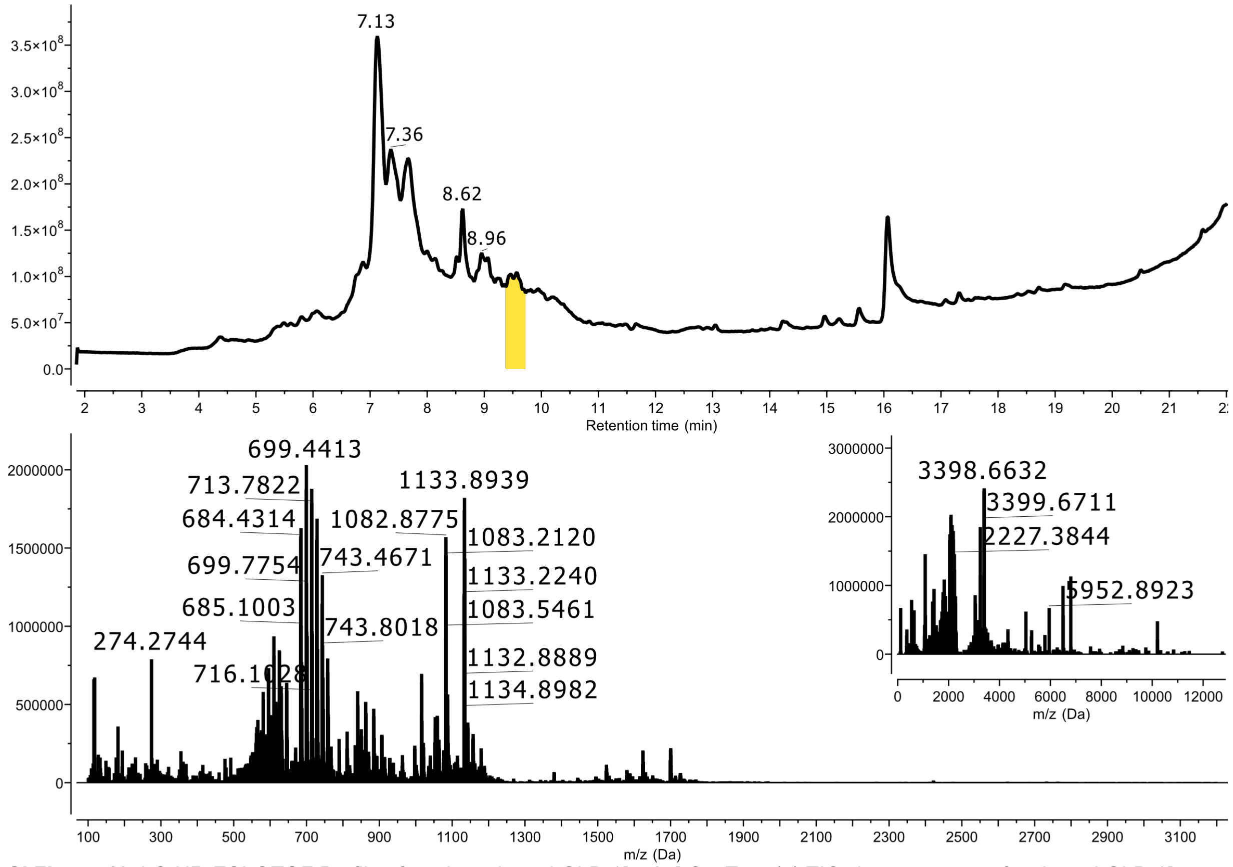

SI Figure 46. LC-HR-ESI-QTOF Profile of crude activated GLP-1[7–37]-SynTag. (a) TIC chromatogram of activated GLP-1[7–37]-SynTag; Rt 9.57 min. (b) ESI-TOF spectrum found within Rt 9.57 min (insert: deconvoluted masses). LCMS Gradient A (**Section 2.8**).

4 Resin screening

4.1 Polystyrene aminomethyl Rink Amide (PSAM-RAM, 0.28 mmol/g loading): Barstar[75–90]

```
                    10        16
     H2N-FREAKAEGCD ITIILS-CONH2      (16 AA)
```

The peptide Barstar[75–90] was synthesized on commercially available polystyrene aminomethyl Rink Amide resin (0.28 mmol/g, 97 mg, 27 µmol), which was manually downloaded according to the procedure described in **Section 2.4,** using the standard AFPS protocol (**Section 2.2.2**) (**SI Figure 47**). Total synthesis time to afford resin-bound Barstar[75–90] was approximately 0.8 h. Cleavage of the peptidyl-resin (38 mg, approx. 11 µmol) according to the cleavage protocol described in **Section 2.6** afforded the crude peptide as a colorless solid (8.1 mg, mass confirmed by LC-HR-ESI-QTOF [**SI Figure 48**], 20% purity by UHPLC [**SI Figure 49**]).

UV-Vis synthesis trace

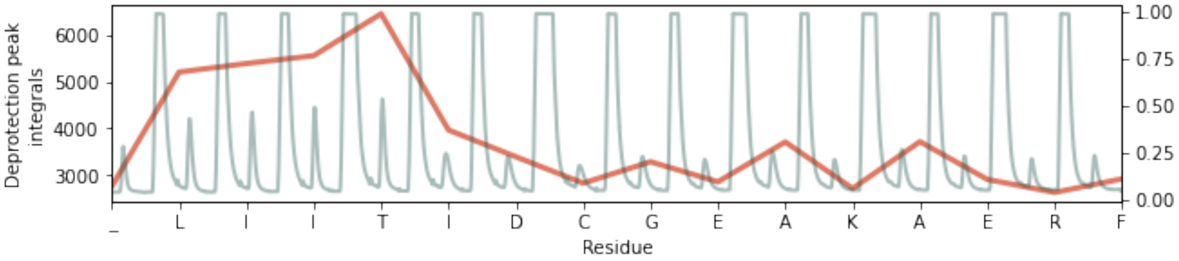

SI Figure 47. UV trace (λ = 310 nm) from AFPS of Barstar[75–90] (green) and deprotection peak integrals (red). Note: UV chromatograms are plotted against time and are not directly aligned with the sequence labels on the x-axis. Only the integrals of the deprotection peaks are aligned with the corresponding amino acid positions.

LC-MS of crude Barstar[75–90]

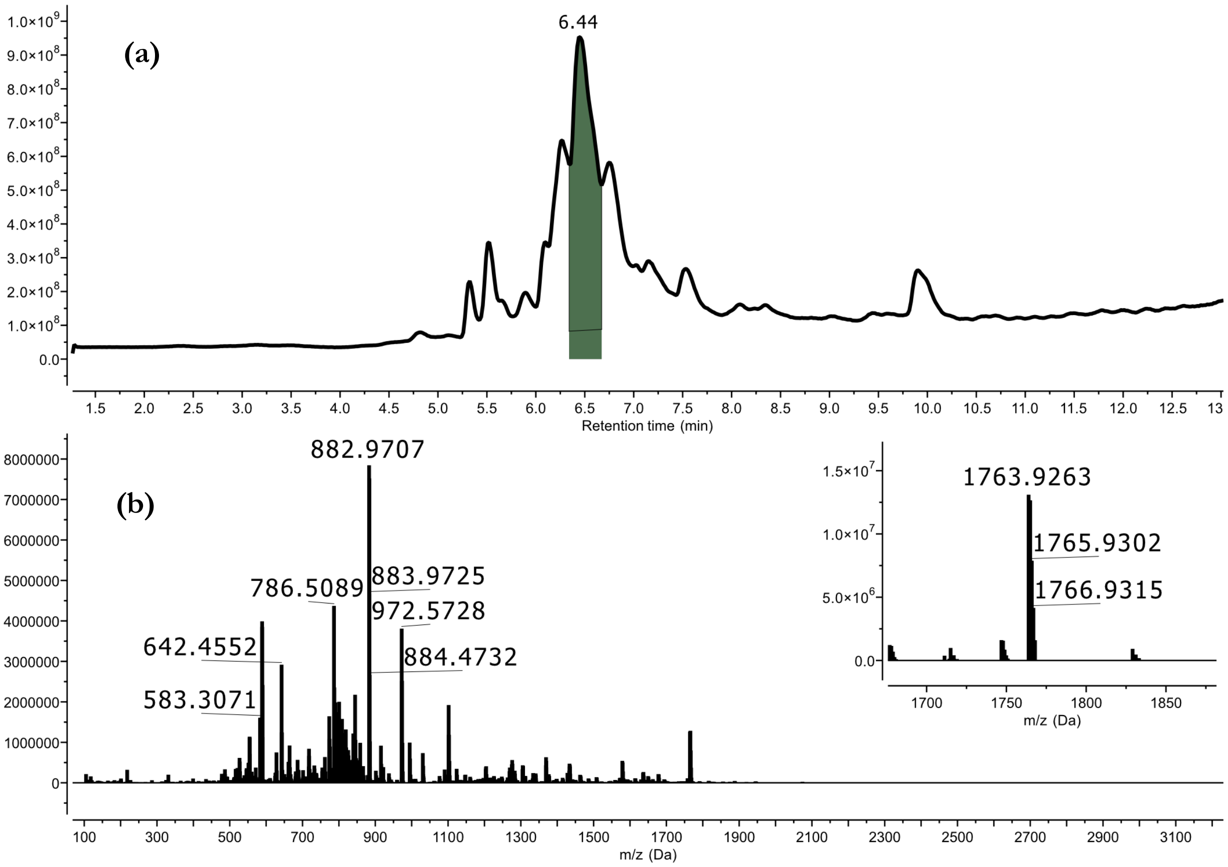

SI Figure 48. LC-HR-ESI-QTOF Profile of crude Barstar[75–90]. (a) TIC chromatogram of Barstar[75–90]; Rt 6.44 min. (b) ESI-TOF spectrum found within Rt 6.44 min (insert: deconvoluted masses). Monoisotopic mass (ESI+) calcd. for $C_{77}H_{129}N_{21}O_{24}S$ 1763.9240, found 1763.9263. LCMS Gradient A (**Section 2.8**).

UHPLC of crude Barstar[75–90]

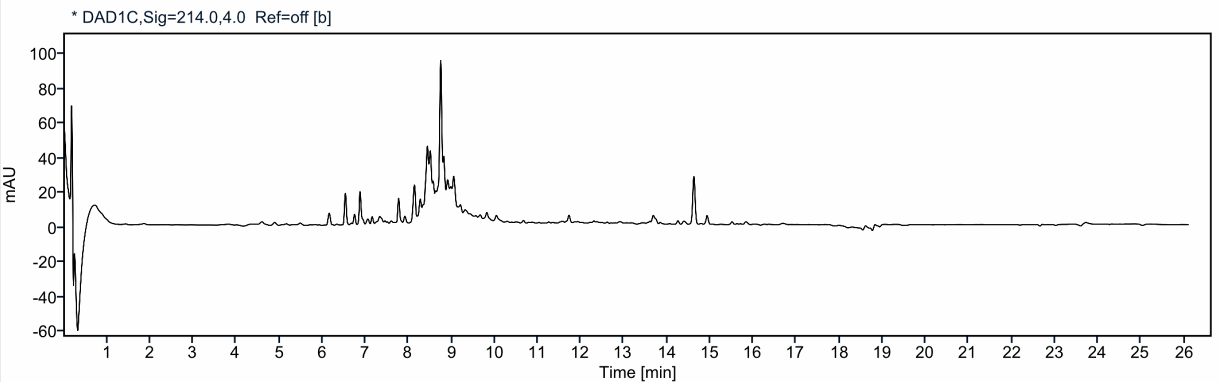

SI Figure 49. UHPLC profile of crude Barstar[75–90]. Rt 8.74 min (Agilent Zorbax 300SB-C18 RRHD column, 1.8 µm, 2.1 × 50 mm, 5–95% MeCN over 20 min, ca. 4.5%B/min), 20% purity based on Area Under Curve (AUC) at λ = 214 nm (**Section 2.7**).

4.2 Polystyrene aminomethyl Rink Amide (PSAM-RAM, 0.28 mmol/g loading): Barstar[75–90]-ArgTag

```
          10                 20
H2N-FREAKAEGCD  ITIILSRRRR RR-CONH2        (22 AA)
```

The peptide Barstar[75–90]-ArgTag was synthesized on commercially available polystyrene aminomethyl Rink Amide resin (0.28 mmol/g, 99 mg, 28 µmol), which was manually downloaded according to the procedure described in **Section 2.4,** using the standard AFPS protocol (**Section 2.2.2**) (**SI Figure 50**). Total synthesis time to afford resin-bound Barstar[75–90]-ArgTag was approximately 1 h. Cleavage of the peptidyl-resin (40 mg, approx. 11 µmol) according to the cleavage protocol described in **Section 2.6** afforded the crude peptide as a colorless solid (10.3 mg, mass confirmed by LC-HR-ESI-QTOF [**SI Figure 51**], 41% purity by UHPLC [**SI Figure 52**]).

UV-Vis synthesis trace

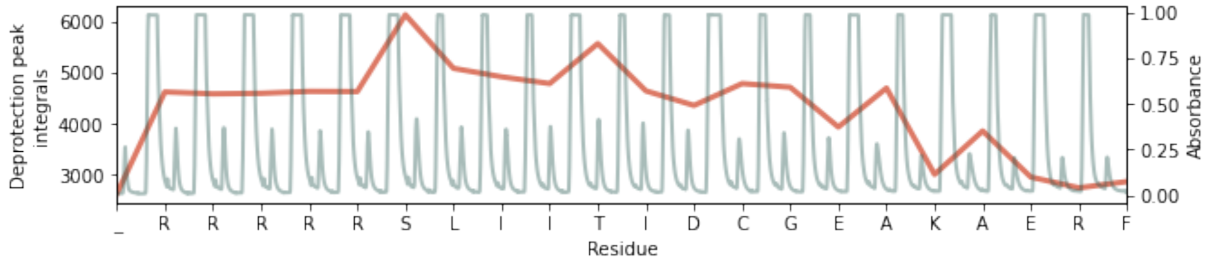

SI Figure 50. UV trace (λ = 310 nm) from AFPS of Barstar[75–90]-ArgTag (green) and deprotection peak integrals (red). Note: UV chromatograms are plotted against time and are not directly aligned with the sequence labels on the x-axis. Only the integrals of the deprotection peaks are aligned with the corresponding amino acid positions.

LC-MS of crude Barstar[75–90]-ArgTag

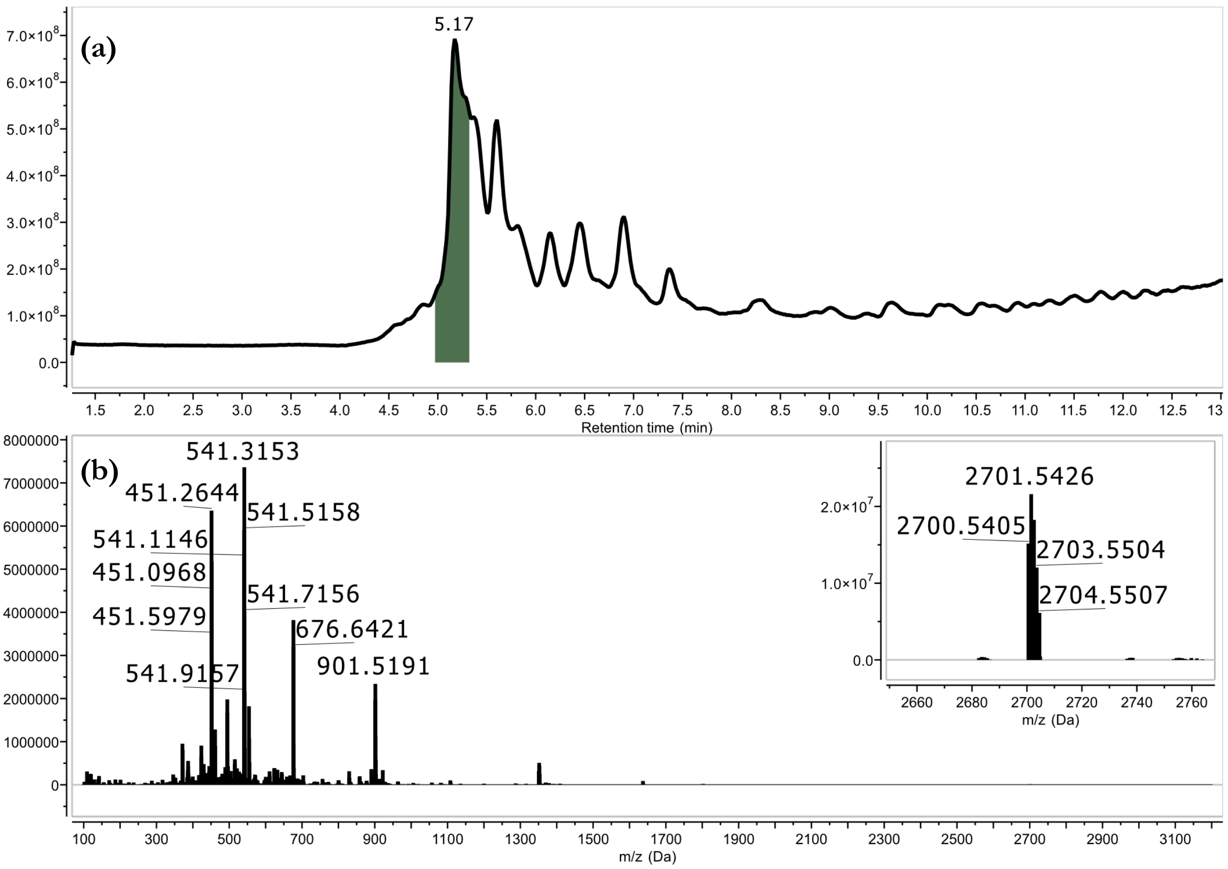

SI Figure 51. LC-HR-ESI-QTOF Profile of crude Barstar[75–90] bearing the ArgTag. (a) TIC chromatogram of Barstar[75–90]-ArgTag; Rt 5.17 min. (b) ESI-TOF spectrum found within Rt 5.17 min (insert: deconvoluted masses). Monoisotopic mass (ESI+) calcd. for $C_{113}H_{201}N_{45}O_{30}S$ 2700.5307, found 2700.5405. LCMS Gradient A (**Section 2.8**).

UHPLC of crude Barstar[75–90]-ArgTag

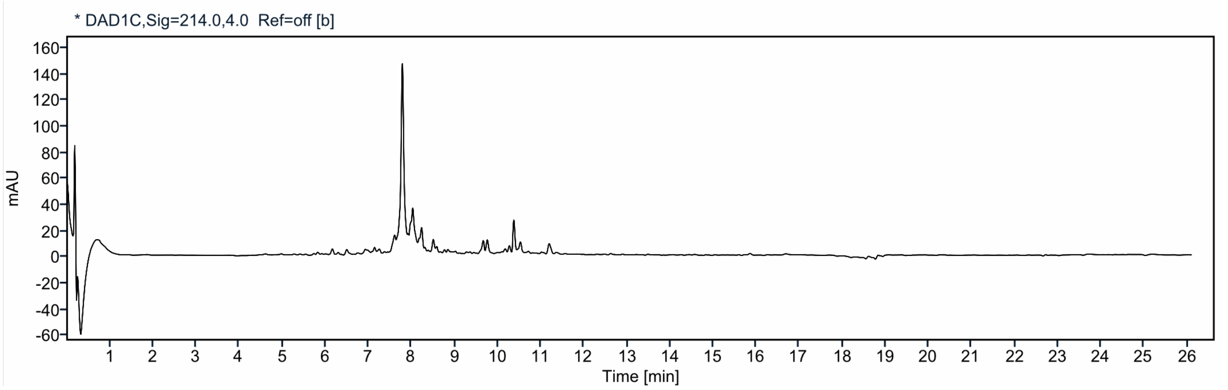

SI Figure 52. UHPLC profile of crude Barstar[75–90] bearing the ArgTag. Rt 7.79 min (Agilent Zorbax 300SB-C18 RRHD column, 1.8 µm, 2.1 × 50 mm, 5–95% MeCN over 20 min, ca. 4.5%B/min), 41% purity based on Area Under Curve (AUC) at λ = 214 nm (**Section 2.7**).

4.3 Polystyrene aminomethyl Rink Amide (PSAM-RAM, 0.60 mmol/g loading): Barstar[75–90]

```
                   10        16
H2N-FREAKAEGCD ITIILS-CONH2       (16 AA)
```

The peptide Barstar[75–90] was synthesized on commercially available polystyrene aminomethyl Rink Amide resin (0.60 mmol/g, 112.3 mg, 67 µmol) using the standard AFPS protocol (**Section 2.2.2**) (**SISI Figure Figure 53**). Total synthesis time to afford resin-bound Barstar[75–90] was approximately 0.8 h. Cleavage of the peptidyl-resin (38 mg, approx. 23 µmol) according to the cleavage protocol described in **Section 2.6** afforded the crude peptide as a colorless solid (14.9 mg, traces of the desired mass confirmed by LC-HR-ESI-QTOF [**SI Figure 54**], 2% purity by UHPLC [**SI Figure 55**]).

UV-Vis synthesis trace

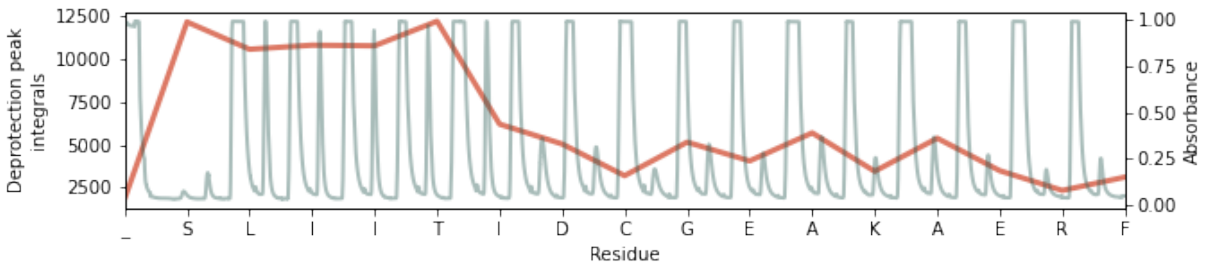

SI Figure 53. UV trace (λ = 310 nm) from AFPS of Barstar[75–90] (green) and deprotection peak integrals (red). Note: UV chromatograms are plotted against time and are not directly aligned with the sequence labels on the x-axis. Only the integrals of the deprotection peaks are aligned with the corresponding amino acid positions.

LC-QTOF of crude Barstar[75–90]

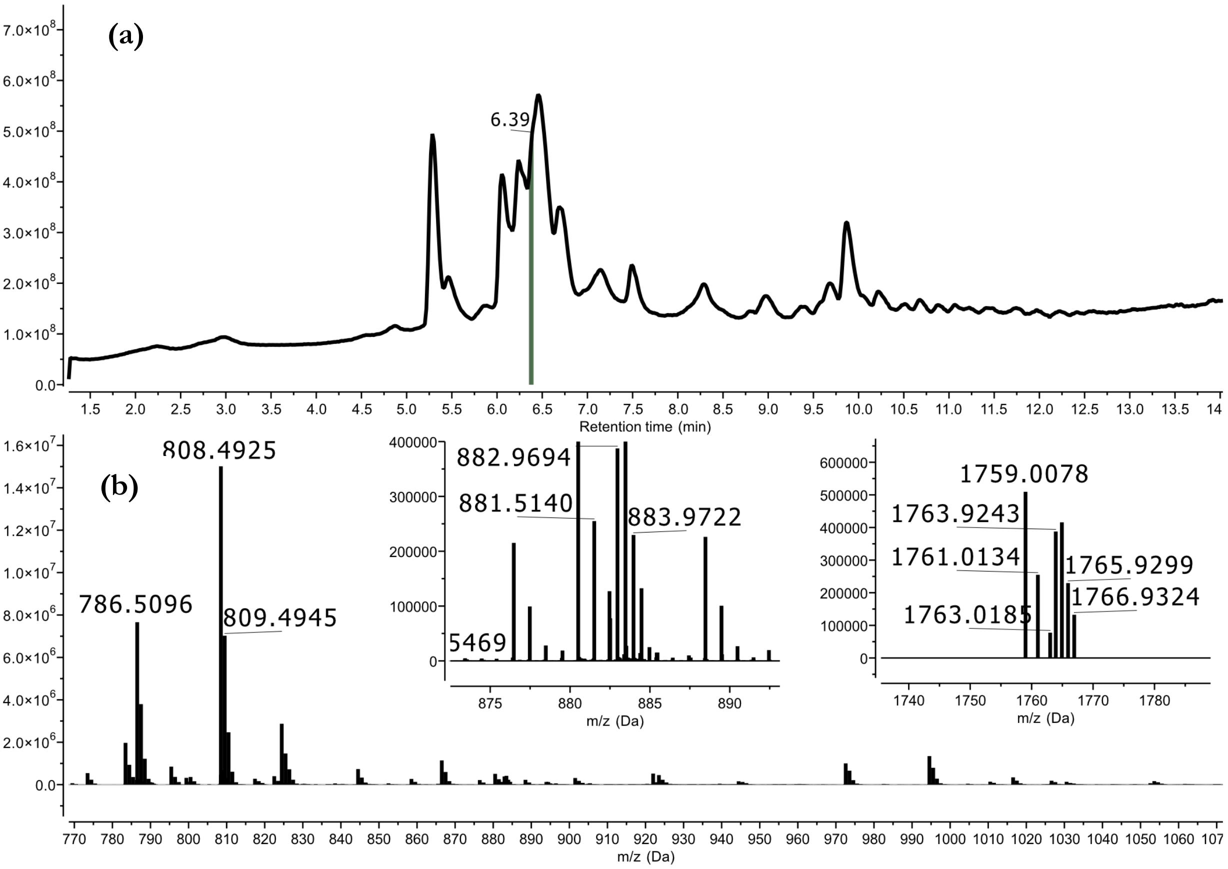

SI Figure 54. LC-HR-ESI-QTOF Profile of crude Barstar[75–90]. (a) TIC chromatogram of Barstar[75–90]; Rt 6.39 min. (b) ESI-TOF spectrum found within Rt 6.39 min (insert left: zoom-in of mass spectrum; insert right: deconvoluted masses). Monoisotopic mass (ESI+) calcd. for $C_{77}H_{129}N_{21}O_{24}S$ 1763.9240, found 1763.9243. LCMS Gradient A (**Section 2.8**).

UHPLC of crude Barstar[75–90]

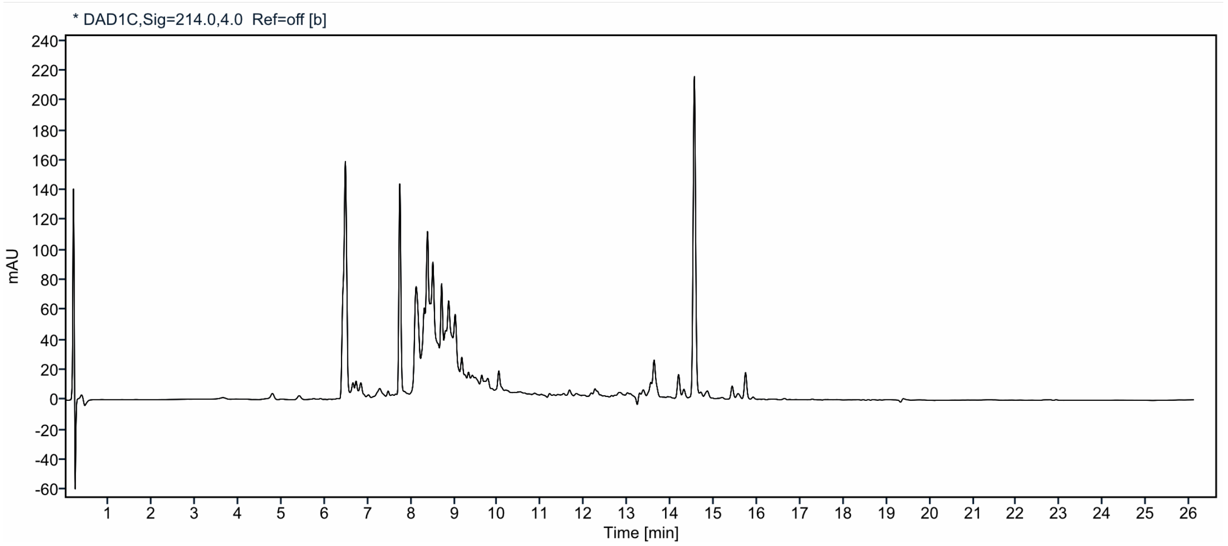

SI Figure 55. UHPLC profile of crude Barstar[75–90]. Rt 8.70 min (Agilent Zorbax 300SB-C18 RRHD column, 1.8 µm, 2.1 × 50 mm, 5–95% MeCN over 20 min, ca. 4.5%B/min), 2% purity based on Area Under Curve (AUC) at λ = 214 nm (**Section 2.7**).

4.4 Polystyrene aminomethyl Rink Amide (PSAM-RAM, 0.60 mmol/g loading): Barstar[75–90]-ArgTag

10 20

H_2N-FREAKAEGCD ITIILS**RRRR** **RR**-$CONH_2$ (22 AA)

The peptide Barstar[75–90]-ArgTag was synthesized on commercially available Rink Amide PSAM resin (0.60 mmol/g, 109.7 mg, 66 μmol) using the standard AFPS protocol (**Section 2.2.2**) (**SI Figure 56**). Total synthesis time to afford resin-bound Barstar[75–90]-ArgTag was approximately 1 h. Cleavage of the peptidyl-resin (37 mg, approx. 22 μmol) according to the cleavage protocol described in **Section 2.6** afforded the crude peptide as a colorless solid (14.7 mg, mass confirmed by LC-HR-ESI-QTOF [**SI Figure 57**], 26% purity by UHPLC [**SI Figure 58**]).

UV-Vis synthesis trace

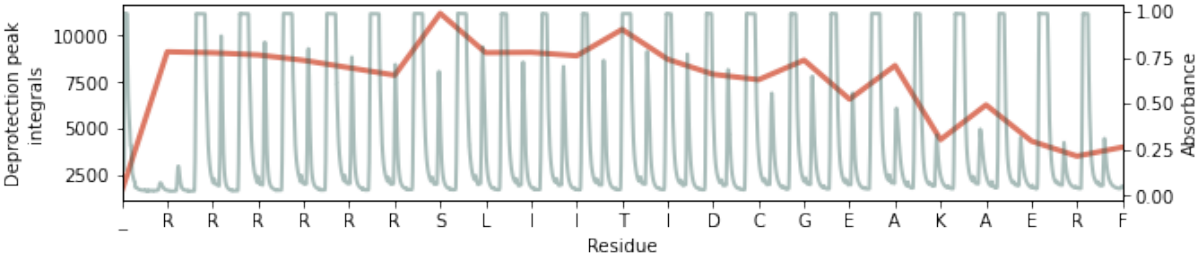

SI Figure 56. UV trace (λ = 310 nm) from AFPS of Barstar[75–90]-ArgTag (green) and deprotection peak integrals (red). Note: UV chromatograms are plotted against time and are not directly aligned with the sequence labels on the x-axis. Only the integrals of the deprotection peaks are aligned with the corresponding amino acid positions.

LC-QTOF of crude Barstar[75–90]-ArgTag

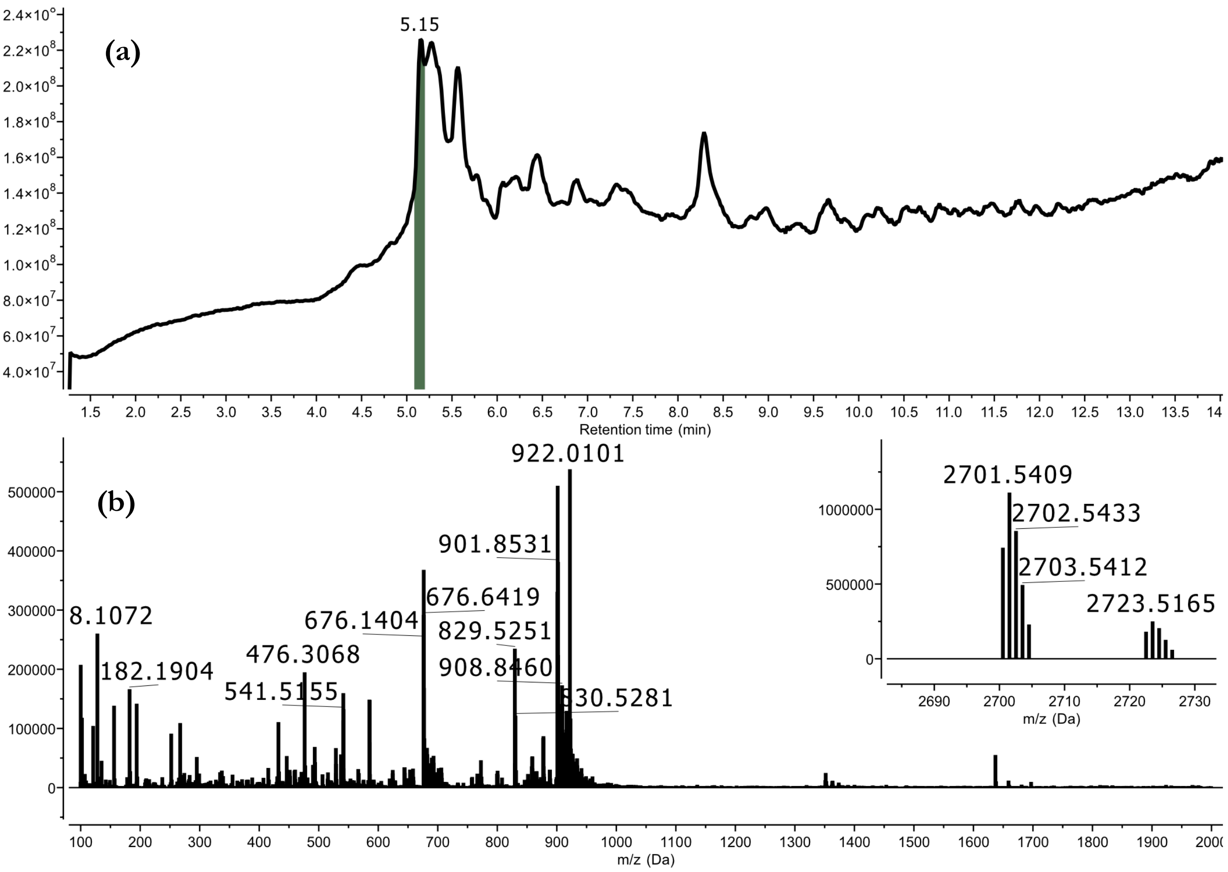

SI Figure 57. LC-HR-ESI-QTOF Profile of crude Barstar[75–90] bearing the ArgTag. (a) TIC chromatogram of Barstar[75–90]-ArgTag; Rt 5.15 min. (b) ESI-TOF spectrum found within Rt 5.15 min (insert: deconvoluted masses). Monoisotopic mass (ESI+) calcd. for $C_{113}H_{201}N_{45}O_{30}S$ 2700.5307, found 2700.5342. LCMS Gradient A (**Section 2.8**).

UHPLC of crude Barstar[75–90]-ArgTag

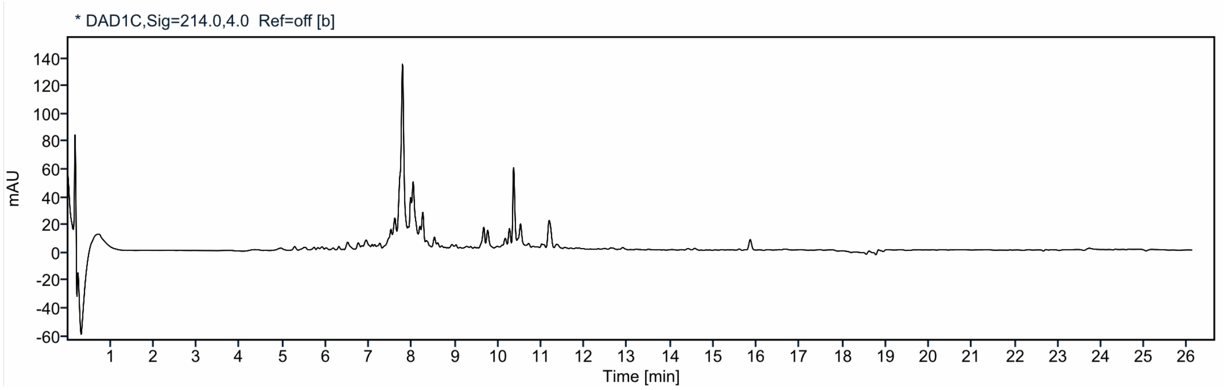

SI Figure 58. UHPLC profile of crude Barstar[75–90] bearing the ArgTag. Rt 7.78 min (Agilent Zorbax 300SB-C18 RRHD column, 1.8 µm, 2.1 × 50 mm, 5–95% MeCN over 20 min, ca. 4.5%B/min), 26% purity based on Area Under Curve (AUC) at λ = 214 nm (**Section 2.7**).

4.5 Methylbenzhydryl amine Rink Amide (MBHA-RAM, 0.35 mmol/g loading): Barstar[75–90]

```
                10        16
H2N-FREAKAEGCD ITIILS-CONH2      (16 AA)
```

The peptide Barstar[75–90] was synthesized on commercially available Rink Amide MBHA resin (0.35 mmol/g, 66 mg, 23 µmol) using the standard AFPS protocol (**Section 2.2.2**) (**SI Figure 59**). Total synthesis time to afford resin-bound Barstar[75–90] was approximately 0.8 h. Cleavage of the peptidyl-resin (27 mg, approx. 9.5 µmol) according to the cleavage protocol described in **Section 2.6** afforded the crude peptide as a colorless solid (5.4 mg, traces of the desired mass confirmed by LC-HR-ESI-QTOF [**SI Figure 60**], 2% purity by UHPLC [**SI Figure 61**]).

UV-Vis synthesis trace

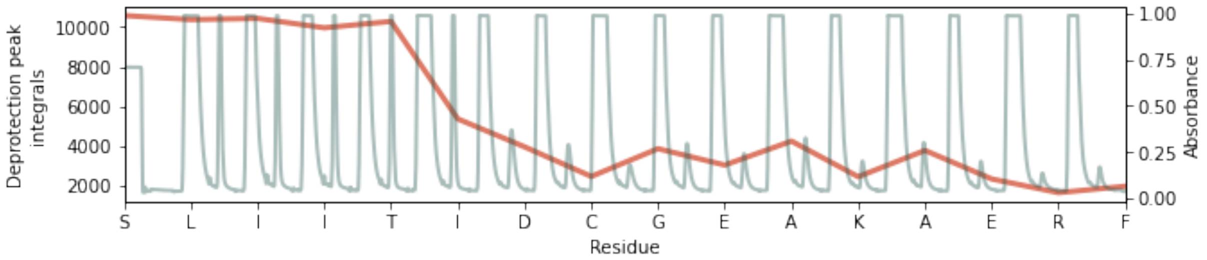

SI Figure 59. UV trace (λ = 310 nm) from AFPS of Barstar[75–90] (green) and deprotection peak integrals (red). Note: UV chromatograms are plotted against time and are not directly aligned with the sequence labels on the x-axis. Only the integrals of the deprotection peaks are aligned with the corresponding amino acid positions.

LC-MS of crude Barstar[75–90]

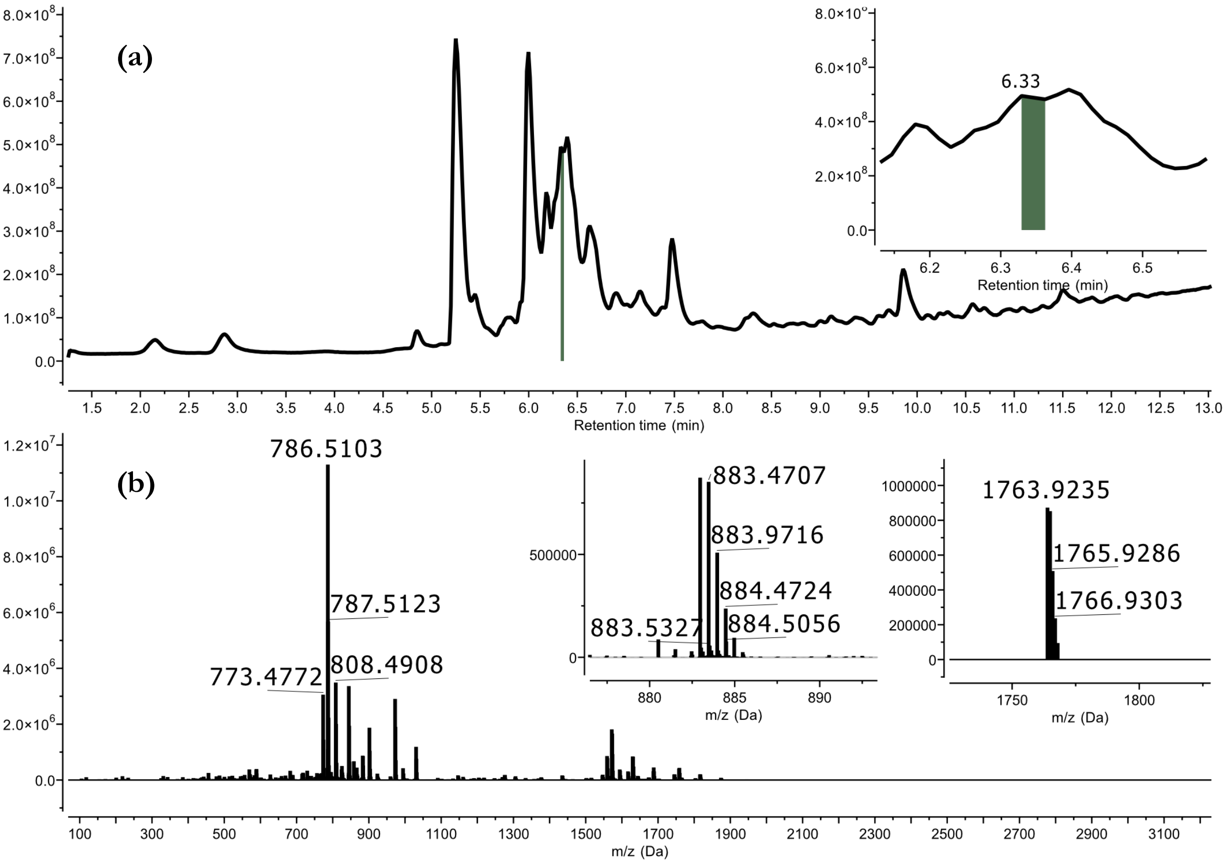

SI Figure 60. LC-HR-ESI-QTOF Profile of crude Barstar[75–90]. (a) TIC chromatogram of Barstar[75–90]; Rt 6.33 min; insert: zoom-in of TIC. (b) ESI-TOF spectrum found within Rt 6.33 min (insert left: zoom-in of mass spectrum; insert right: deconvoluted masses). Monoisotopic mass (ESI+) calcd. for $C_{77}H_{129}N_{21}O_{24}S$ 1763.9240, found 1763.9235. LCMS Gradient A (**Section 2.8**).

UHPLC of crude Barstar[75–90]

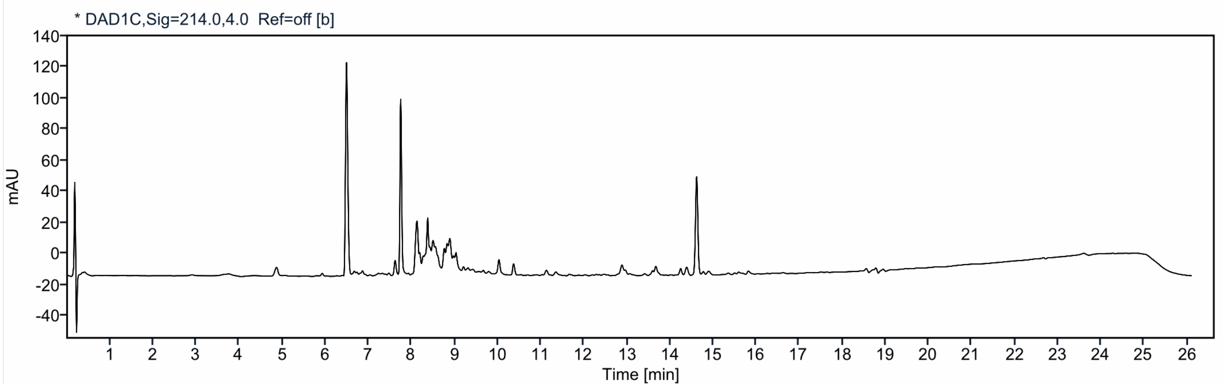

SI Figure 61. UHPLC profile of crude Barstar[75–90]. Rt 8.75 min (Agilent Zorbax 300SB-C18 RRHD column, 1.8 µm, 2.1 × 50 mm, 5–95% MeCN over 20 min, ca. 4.5%B/min), 2% purity based on Area Under Curve (AUC) at λ = 214 nm (**Section 2.7**).

4.6 Methylbenzhydryl amine Rink Amide (MBHA-RAM, 0.35 mmol/g loading): Barstar[75–90]-ArgTag

```
                    10          20
H2N-FREAKAEGCD  ITIILSRRRR RR-CONH2        (22 AA)
```

The peptide Barstar[75–90]-ArgTag was synthesized on commercially available Rink Amide MBHA resin (0.35 mmol/g, 68 mg, 24 µmol) using the standard AFPS protocol (**Section 2.2.2**) (**SI Figure**

62). Total synthesis time to afford resin-bound Barstar[75–90]-ArgTag was approximately 1 h. Cleavage of the peptidyl-resin (37 mg, approx. 13 μmol) according to the cleavage protocol described in **Section 2.6** afforded the crude peptide as a colorless solid (8.1 mg, mass confirmed by LC-HR-ESI-QTOF [**SI Figure 63**], 24% purity by UHPLC [**SI Figure 64**]).

UV-Vis synthesis trace

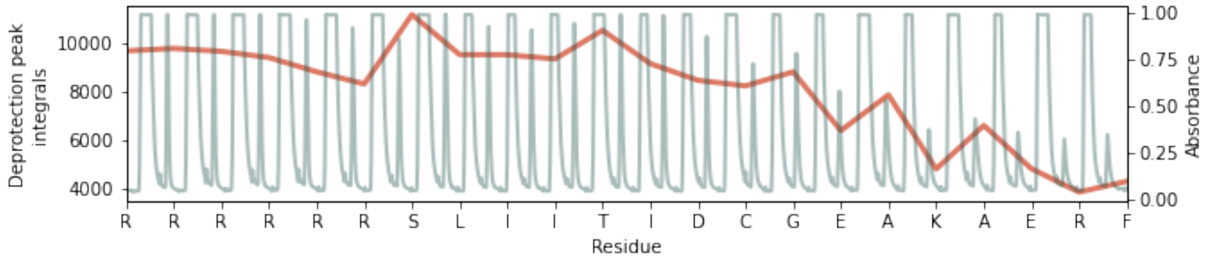

SI Figure 62. UV trace (λ = 310 nm) from AFPS of Barstar[75–90]-ArgTag (green) and deprotection peak integrals (red). Note: UV chromatograms are plotted against time and are not directly aligned with the sequence labels on the x-axis. Only the integrals of the deprotection peaks are aligned with the corresponding amino acid positions.

LC-MS of crude Barstar[75–90]-ArgTag

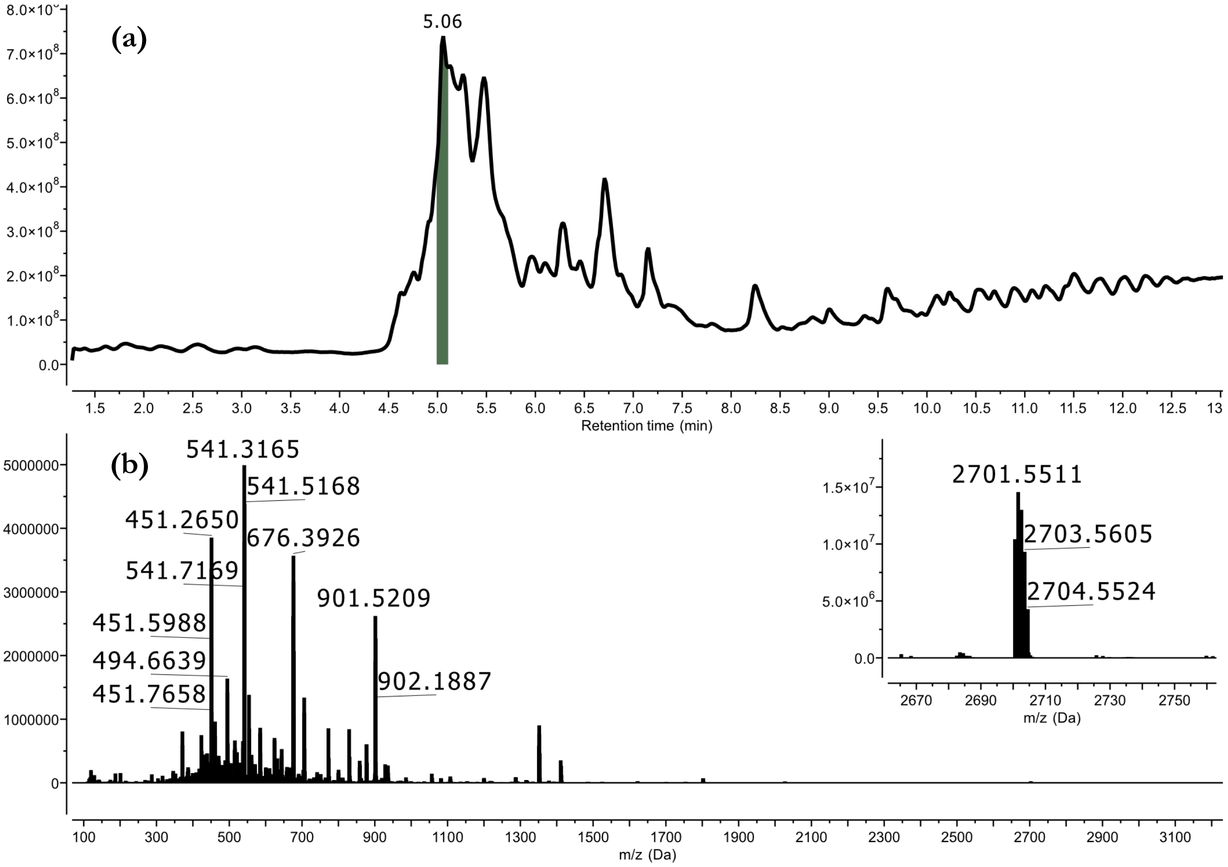

SI Figure 63. LC-HR-ESI-QTOF Profile of crude Barstar[75–90] bearing the ArgTag. (a) TIC chromatogram of Barstar[75–90]-ArgTag; Rt 5.06 min. (b) ESI-TOF spectrum found within Rt 5.06 min (insert: deconvoluted masses). Monoisotopic mass (ESI+) calcd. for $C_{113}H_{201}N_{45}O_{30}S$ 2700.5307, found 2700.5478. LCMS Gradient A (**Section 2.8**).

UHPLC of crude Barstar[75–90]-ArgTag

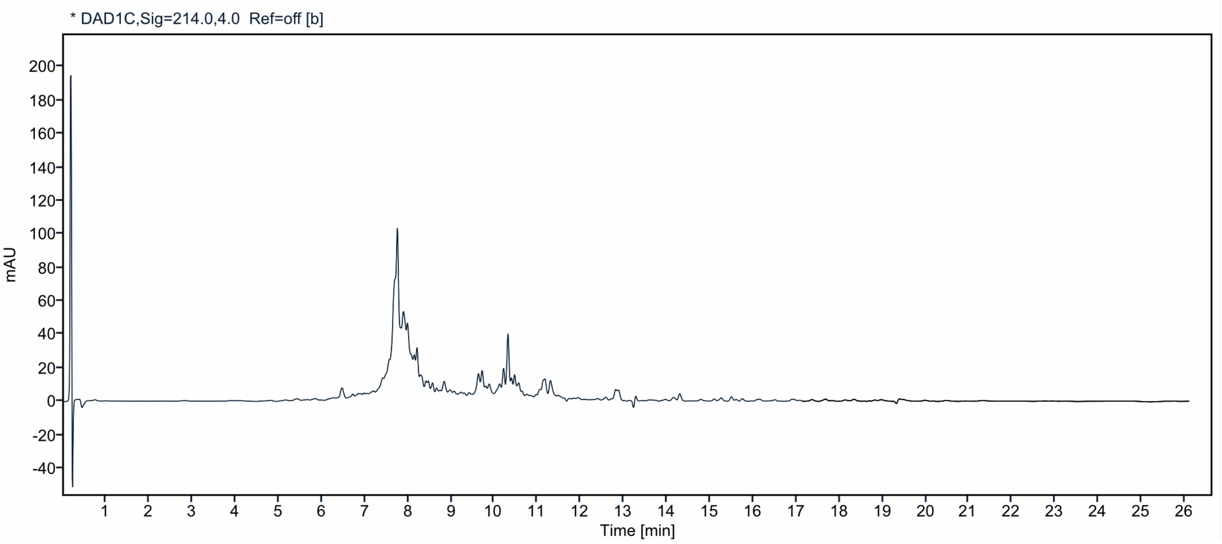

SI Figure 64. UHPLC profile of crude Barstar[75–90] bearing ArgTag. Rt 7.75 min (Agilent Zorbax 300SB-C18 RRHD column, 1.8 µm, 2.1 × 50 mm, 5–95% MeCN over 20 min, ca. 4.5%B/min), 24% purity based on Area Under Curve (AUC) at λ = 214 nm (**Section 2.7**).

4.7 Rink Amide TentagelXV (TGXV-RAM, 0.23 mmol/g loading): Barstar[75–90]

```
                  10        16
H2N-FREAKAEGCD ITIILS-CONH2       (16 AA)
```

The peptide Barstar[75–90] was synthesized on commercially available Rink Amide TentagelXV resin (0.23 mmol/g, 105.1 mg, 24 µmol) using the standard AFPS protocol (**Section 2.2.2**) (**SI Figure 65**). Total synthesis time to afford resin-bound Barstar[75–90] was approximately 0.8 h. Cleavage of the peptidyl-resin (48 mg, approx. 11.0 µmol) according to the cleavage protocol described in **Section 2.6** afforded the crude peptide as a colorless solid (11.1 mg, mass confirmed by LC-HR-ESI-QTOF [**SI Figure 66**], 46% purity by UHPLC [**SI Figure 67**]).

UV-Vis synthesis trace

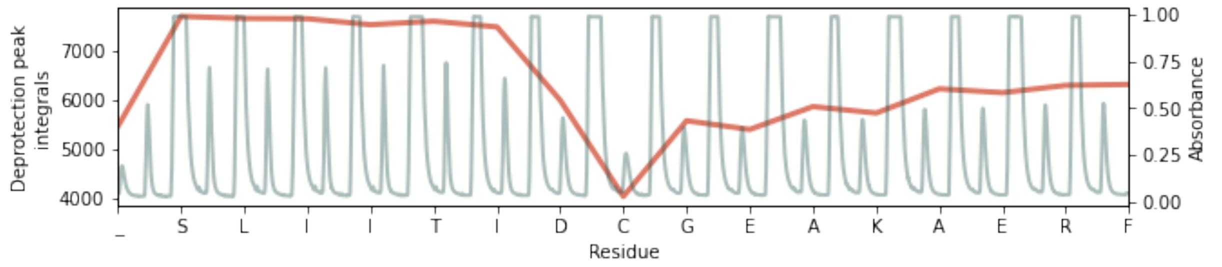

SI Figure 65. UV trace (λ = 310 nm) from AFPS of Barstar[75–90] (green) and deprotection peak integrals (red). Note: UV chromatograms are plotted against time and are not directly aligned with the sequence labels on the x-axis. Only the integrals of the deprotection peaks are aligned with the corresponding amino acid positions.

LC-MS of crude Barstar[75–90]

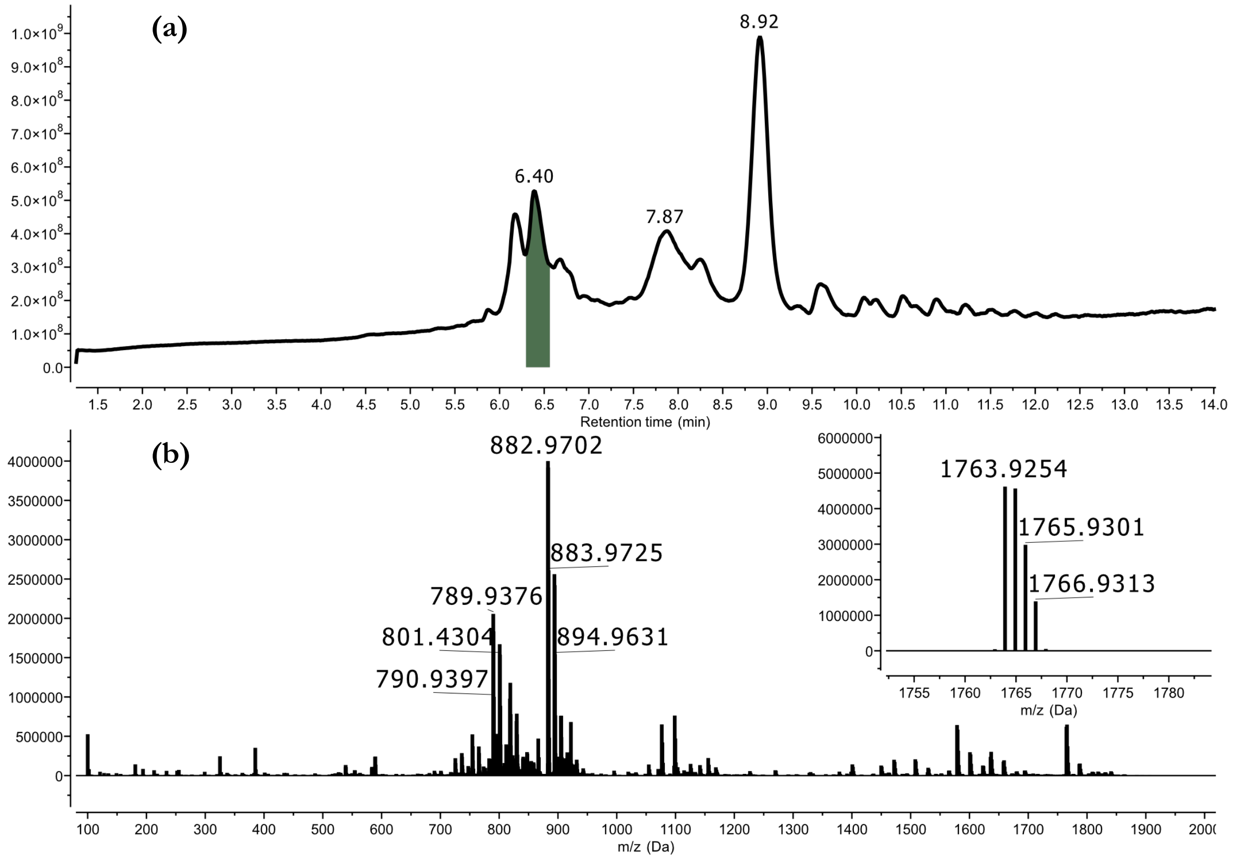

SI Figure 66. LC-HR-ESI-QTOF Profile of crude Barstar[75–90]. (a) TIC chromatogram of Barstar[75–90]; Rt 6.40 min. Peaks at 7.87 min and 8.92 min correspond to known PEG leaching from TGXV-RAM resin. (b) ESI-TOF spectrum found within Rt 6.40 min (insert deconvoluted masses). Monoisotopic mass (ESI+) calcd. for $C_{77}H_{129}N_{21}O_{24}S$ 1763.9240, found 1763.9254. LCMS Gradient A (**Section 2.8**).

UHPLC of crude Barstar[75–90]

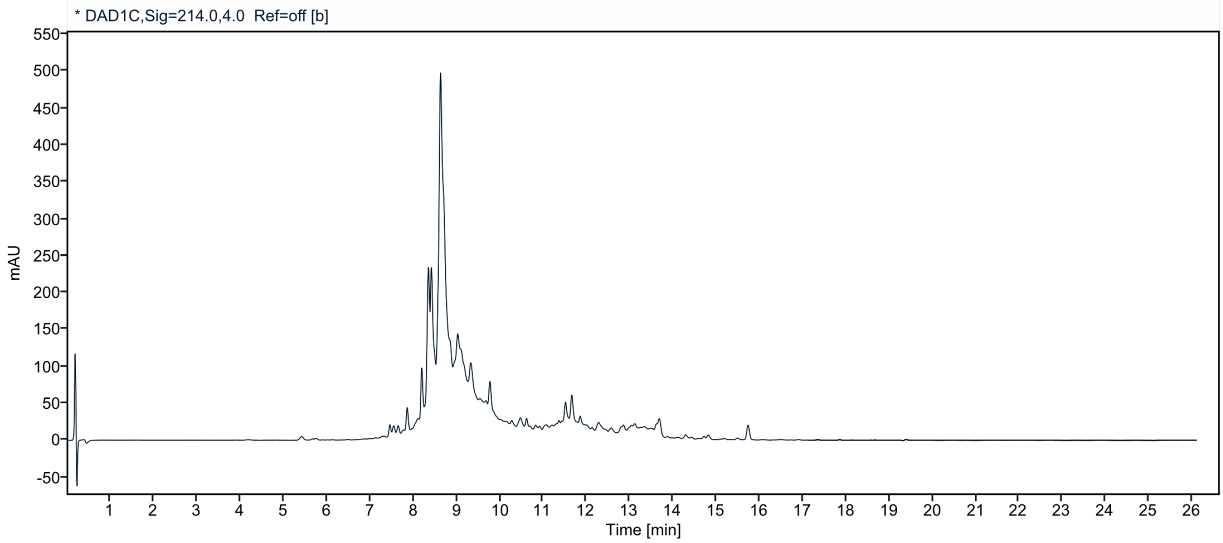

SI Figure 67. UHPLC profile of crude Barstar[75–90]. Rt 8.63 min (Agilent Zorbax 300SB-C18 RRHD column, 1.8 µm, 2.1 × 50 mm, 5–95% MeCN over 20 min, ca. 4.5%B/min), 46% purity based on Area Under Curve (AUC) at λ = 214 nm.

4.8 **Rink Amide TentagelXV (TGXV-RAM, 0.23 mmol/g loading):** Barstar[75–90]-ArgTag

```
           10          20
H2N-FREAKAEGCD  ITIILSRRRR RR-CONH2        (22 AA)
```

The peptide Barstar[75–90]-ArgTag was synthesized on commercially available Rink Amide TGXV resin (0.23 mmol/g, 103.9 mg, 24 μmol) using the standard AFPS protocol (**Section 2.2.2**) (**SI Figure 68**). Total synthesis time to afford resin-bound Barstar[75–90]-ArgTag was approximately 1 h. Cleavage of the peptidyl-resin (48 mg, approx. 11 μmol) according to the cleavage protocol described in **Section 2.6** afforded the crude peptide as a colorless solid (13.1 mg, mass confirmed by LC-HR-ESI-QTOF [**SI Figure 69**], 72% purity by UHPLC [**SI Figure 70**]).

UV-Vis synthesis trace

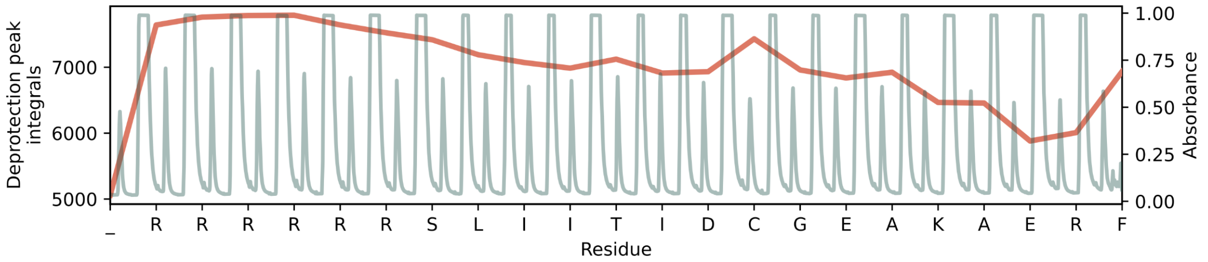

SI Figure 68. UV trace (λ = 310 nm) from AFPS of Barstar[75–90]-ArgTag (green) and deprotection peak integrals (red). Note: UV chromatograms are plotted against time and are not directly aligned with the sequence labels on the x-axis. Only the integrals of the deprotection peaks are aligned with the corresponding amino acid positions.

UHPLC of crude Barstar[75–90]-ArgTag

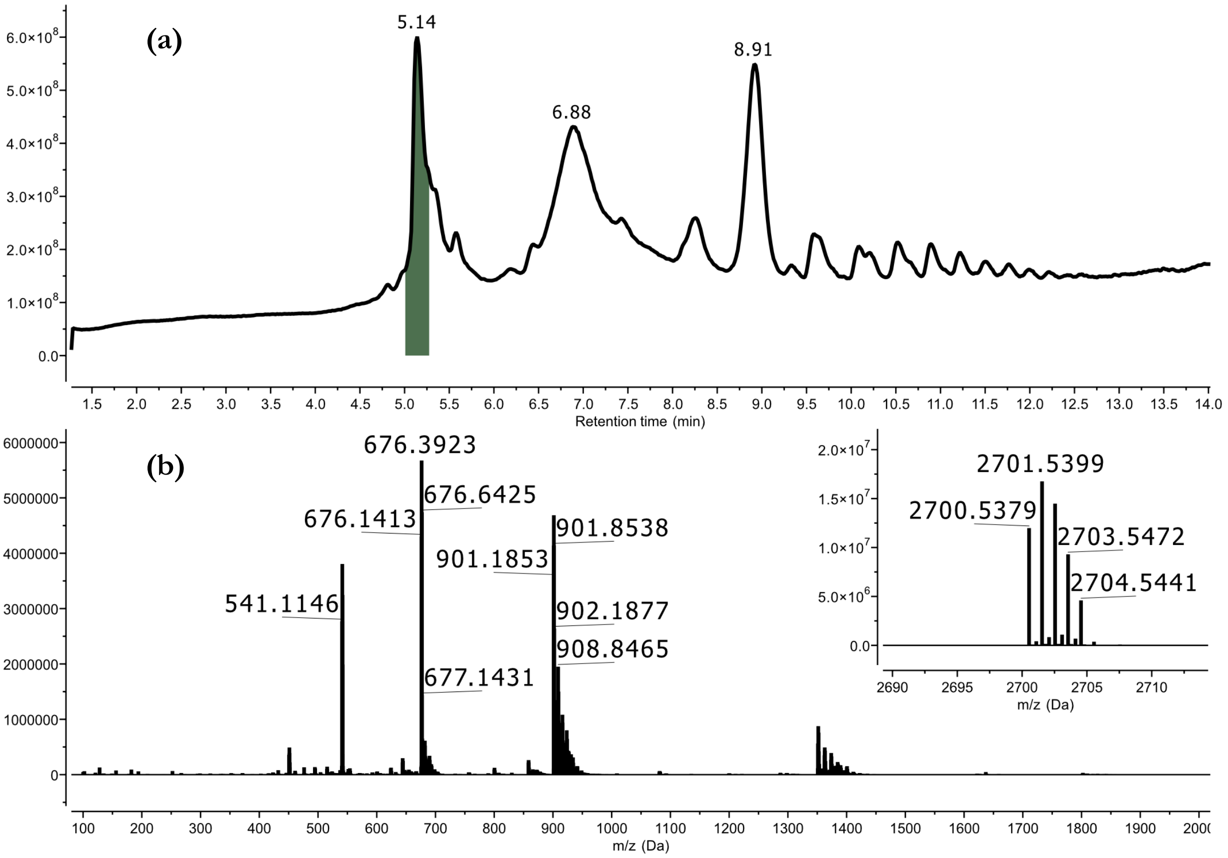

SI Figure 69. LC-HR-ESI-QTOF Profile of crude Barstar[75–90] bearing the ArgTag. (a) TIC chromatogram of Barstar[75–90]-ArgTag; Rt 5.14 min. (b) ESI-TOF spectrum found within Rt 5.14 min (insert: deconvoluted masses). Monoisotopic mass (ESI+) calcd. for $C_{113}H_{201}N_{45}O_{30}S$ 2700.5307, found 2700.5379. LCMS Gradient A (**Section 2.8**).

UHPLC of crude Barstar[75–90]-ArgTag

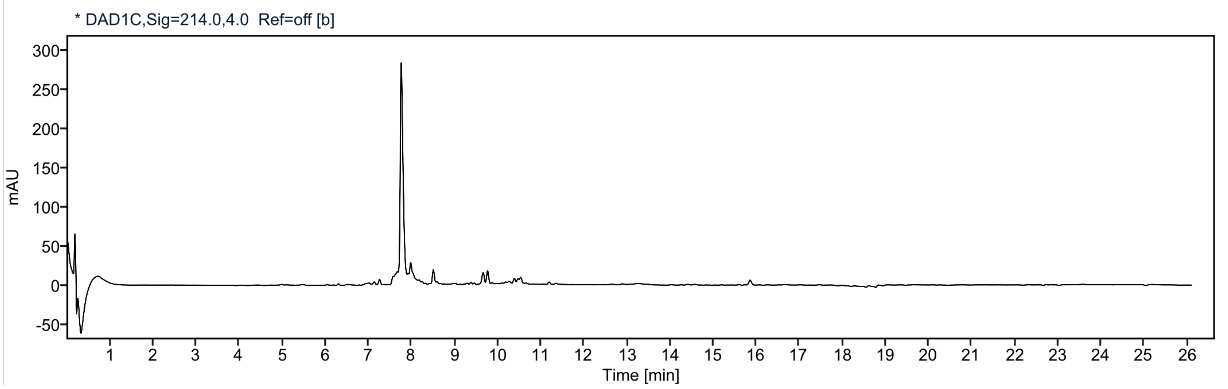

SI Figure 70. UHPLC profile of crude Barstar[75–90] bearing ArgTag. Rt 7.75 min (Agilent Zorbax 300SB-C18 RRHD column, 1.8 µm, 2.1 × 50 mm, 5–95% MeCN over 20 min, ca. 4.5%B/min), 72% purity based on Area Under Curve (AUC) at λ = 214 nm.

4.9 Rink Amide NovaGel (NovaGel-RAM, 0.25 mmol/g loading): Barstar[75–90]

```
                 10         16
H2N-FREAKAEGCD ITIILS-CONH2       (16 AA)
```

The peptide Barstar[75–90] was synthesized on commercially available Rink Amide NovaGel resin (0.25 mmol/g, 107.2 mg, 27 μmol), which was manually downloaded according to the procedure described in **Section 2.4,** using the standard AFPS protocol (**Section 2.2.2**) (**SI Figure 71**). Total synthesis time to afford resin-bound Barstar[75–90] was approximately 0.8 h. Cleavage of the peptidyl-resin (32 mg, approx. 8.0 μmol) according to the cleavage protocol described in **Section 2.6** afforded the crude peptide as a colorless solid (6.8 mg, mass confirmed by LC-HR-ESI-QTOF [**SI Figure 72**], 52% purity by UHPLC [**SI Figure 73**]).

UV-Vis synthesis trace

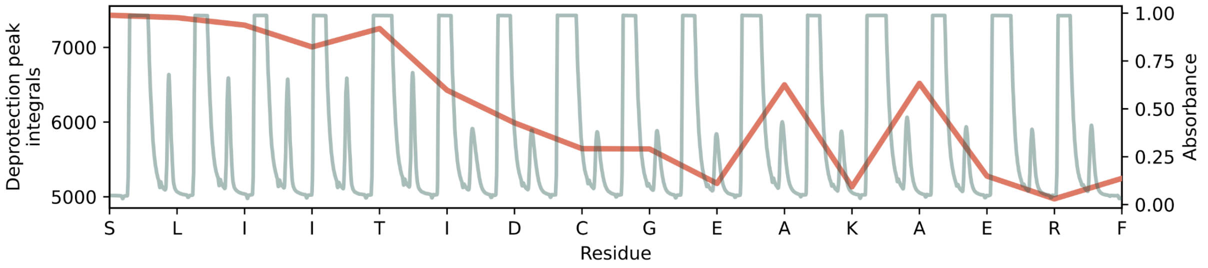

SI Figure 71. UV trace (λ = 310 nm) from AFPS of Barstar[75–90] (green) and deprotection peak integrals (red). Note: UV chromatograms are plotted against time and are not directly aligned with the sequence labels on the x-axis. Only the integrals of the deprotection peaks are aligned with the corresponding amino acid positions.

LC-MS of crude Barstar[75–90]

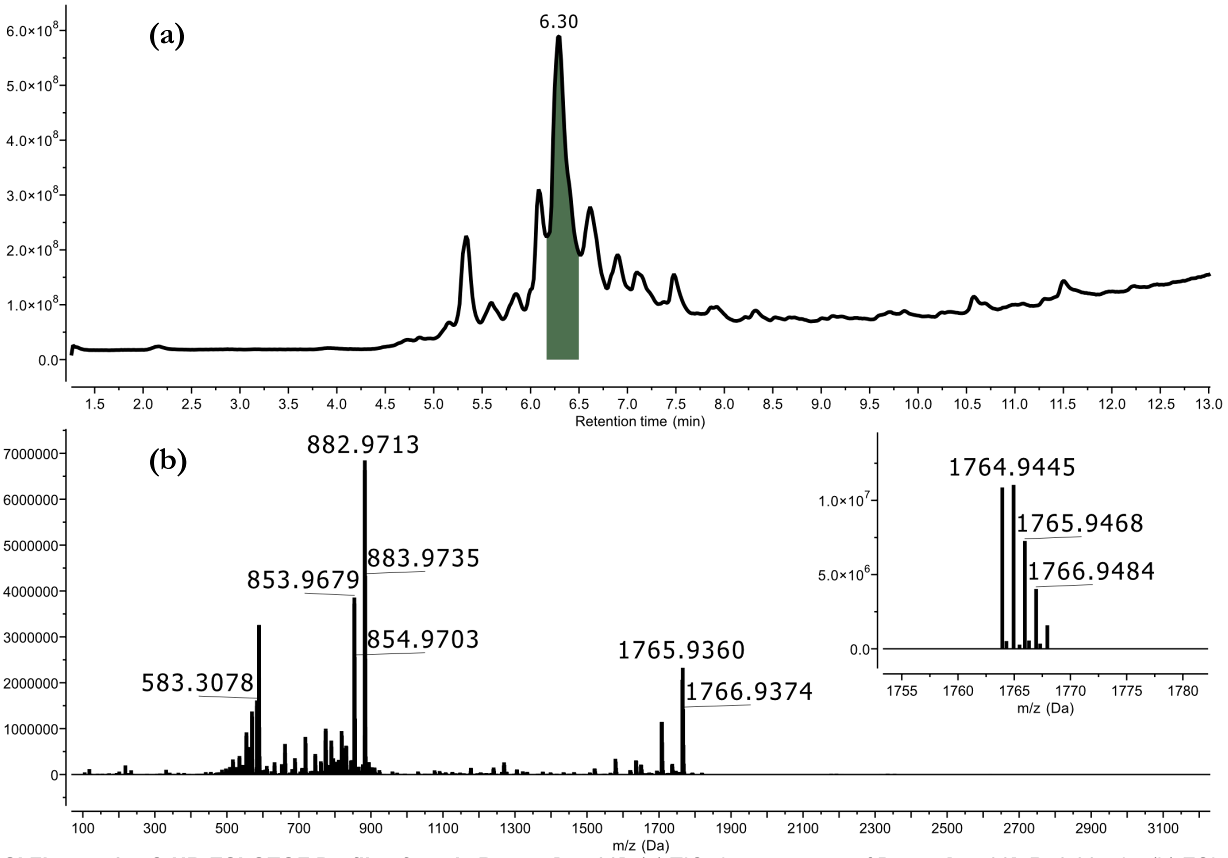

SI Figure 72. LC-HR-ESI-QTOF Profile of crude Barstar[75–90]. (a) TIC chromatogram of Barstar[75–90]; Rt 6.30 min. (b) ESI-TOF spectrum found within Rt 6.30 min (insert deconvoluted masses). Monoisotopic mass (ESI+) calcd. for $C_{77}H_{129}N_{21}O_{24}S$ 1763.9240, found 1763.9374. LCMS Gradient A (**Section 2.8**).

UHPLC of crude Barstar[75–90]

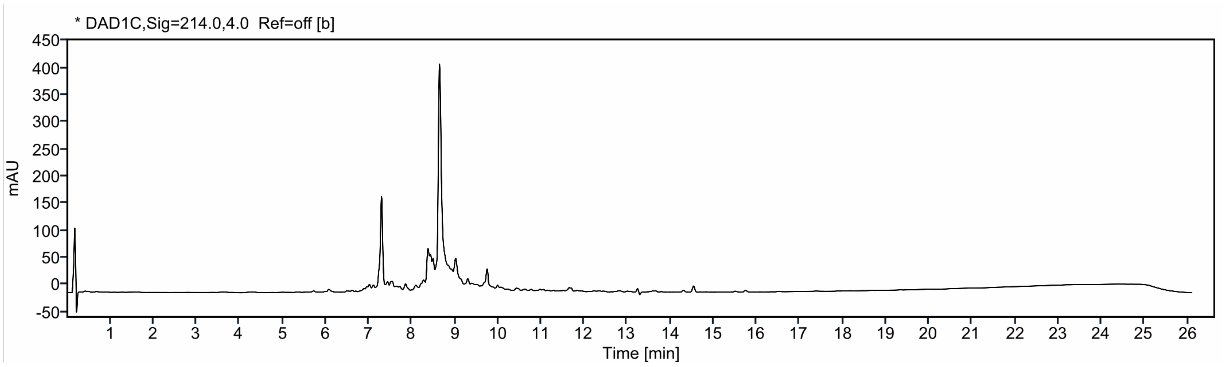

SI Figure 73. UHPLC profile of crude Barstar[75–90]. Rt 8.64 min (Agilent Zorbax 300SB-C18 RRHD column, 1.8 µm, 2.1 × 50 mm, 5–95% MeCN over 20 min, ca. 4.5%B/min), 52% purity based on Area Under Curve (AUC) at λ = 214 nm.

4.10 Rink Amide NovaGel (NovaGel-RAM, 0.25 mmol/g loading): Barstar[75–90]-ArgTag

10 20

H_2N-FREAKAEGCD ITIILS**RRRR** **RR**-$CONH_2$ (22 AA)

The peptide Barstar[75–90]-ArgTag was synthesized on commercially available Rink Amide NovaGel resin (0.25 mmol/g, 107.2 mg, 27 µmol), which was manually downloaded according to the procedure described in **Section 2.4,** using the standard AFPS protocol (**Section 2.2.2**) (**SI Figure 74**). Total synthesis time to afford resin-bound Barstar[75–90]-ArgTag was approximately 1 h. Cleavage of the peptidyl-resin (47 mg, approx. 12 µmol) according to the cleavage protocol described in **Section 2.6** afforded the crude peptide as a colorless solid (8.6 mg, mass confirmed by LC-HR-ESI-QTOF [**SI Figure 75**], 58% purity by UHPLC [**SI Figure 76**]).

UV-Vis synthesis trace

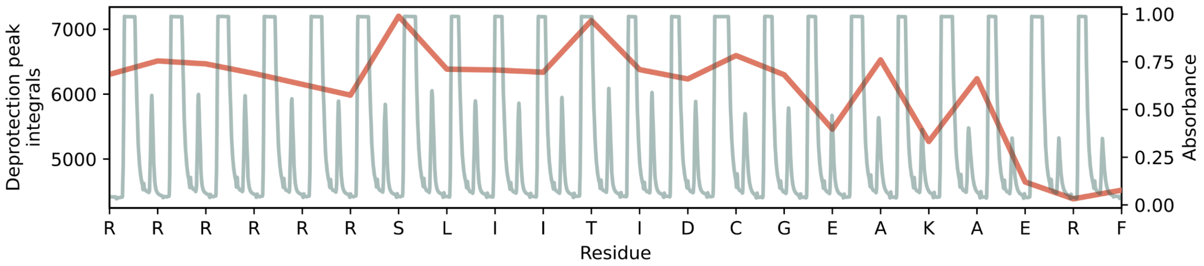

SI Figure 74. UV trace (λ = 310 nm) from AFPS of Barstar[75–90]-ArgTag (green) and deprotection peak integrals (red). Note: UV chromatograms are plotted against time and are not directly aligned with the sequence labels on the x-axis. Only the integrals of the deprotection peaks are aligned with the corresponding amino acid positions.

LC-QTOF of crude Barstar[75–90]-ArgTag

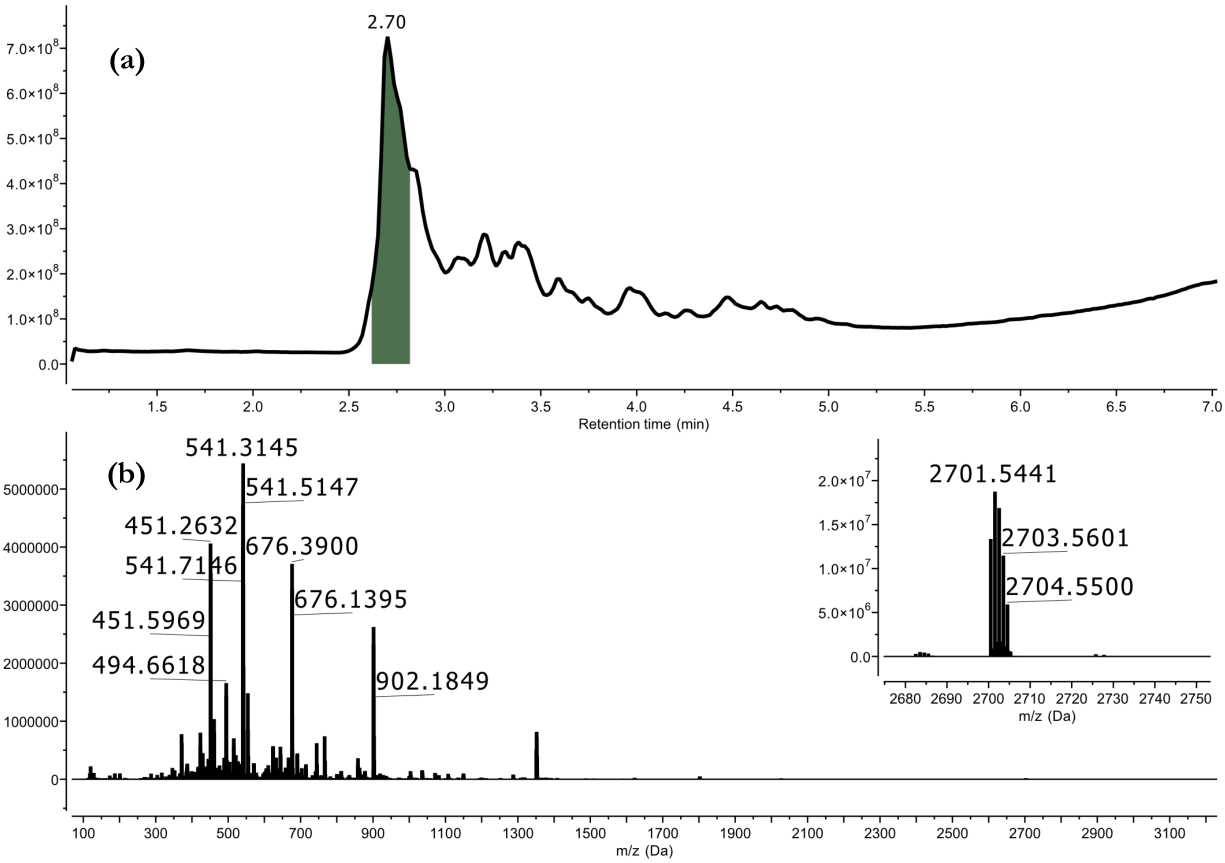

SI Figure 75. LC-HR-ESI-QTOF Profile of crude Barstar[75–90] bearing the ArgTag. (a) TIC chromatogram of Barstar[75–90]-ArgTag; Rt 2.70 min. (b) ESI-TOF spectrum found within Rt 2.70 min (insert: deconvoluted masses). Monoisotopic mass (ESI+) calcd. for $C_{113}H_{201}N_{45}O_{30}S$ 2700.5307, found 2700.5399. LCMS Gradient B (**Section 2.8**).

UHPLC of crude Barstar[75–90]-ArgTag

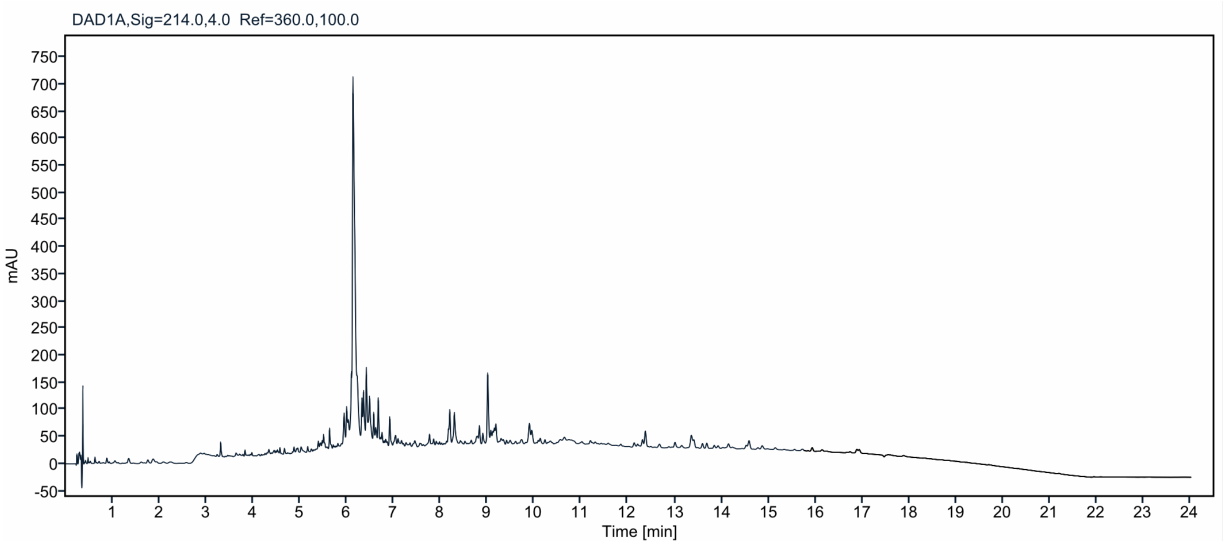

SI Figure 76. UHPLC profile of crude Barstar[75–90] bearing ArgTag. Rt 6.14 min (Agilent Zorbax 300SB-C18 RRHD column, 1.8 µm, 2.1 × 50 mm, 5–95% MeCN over 20 min, ca. 4.5%B/min), 58% purity based on Area Under Curve (AUC) at λ = 214 nm. Chromatogram measured on another device of the exact same type causing the retention time shift compared to other examples of Barstar[75–90] bearing ArgTag.

4.11 Rink Amide NovaGel (NovaGel-RAM, 0.54 mmol/g loading): Barstar[75–90]

```
                 10       16
H2N-FREAKAEGCD ITIILS-CONH2       (16 AA)
```

The peptide Barstar[75–90] was synthesized on commercially available Rink Amide NovaGel resin (0.54 mmol/g, 58.2 mg, 31 μmol) using the standard AFPS protocol (**Section 2.2.2**) (**SI Figure 77**). Total synthesis time to afford resin-bound Barstar[75–90] was approximately 0.8 h. Cleavage of the peptidyl-resin (44 mg, approx. 24 μmol) according to the cleavage protocol described in **Section 2.6** afforded the crude peptide as a colorless solid (13.3 mg, mass confirmed by LC-HR-ESI-QTOF [**SI Figure 78**], 23% purity by UHPLC [**SI Figure 79**]).

UV-Vis synthesis trace

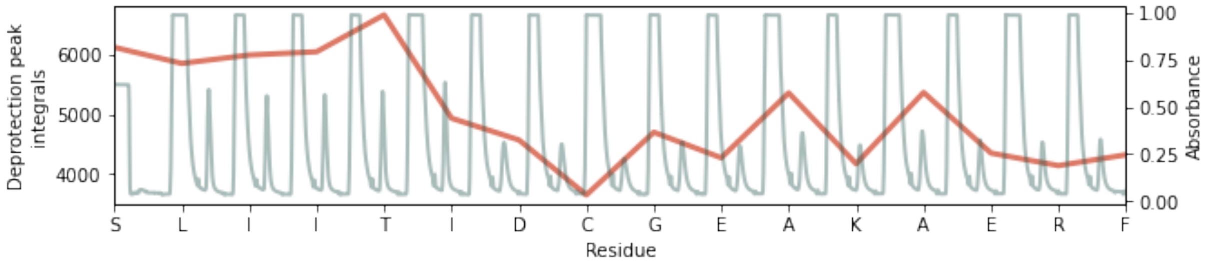

SI Figure 77. UV trace (λ = 310 nm) from AFPS of Barstar[75–90] (green) and deprotection peak integrals (red). Note: UV chromatograms are plotted against time and are not directly aligned with the sequence labels on the x-axis. Only the integrals of the deprotection peaks are aligned with the corresponding amino acid positions.

LC-QTOF-of crude Barstar[75–90]

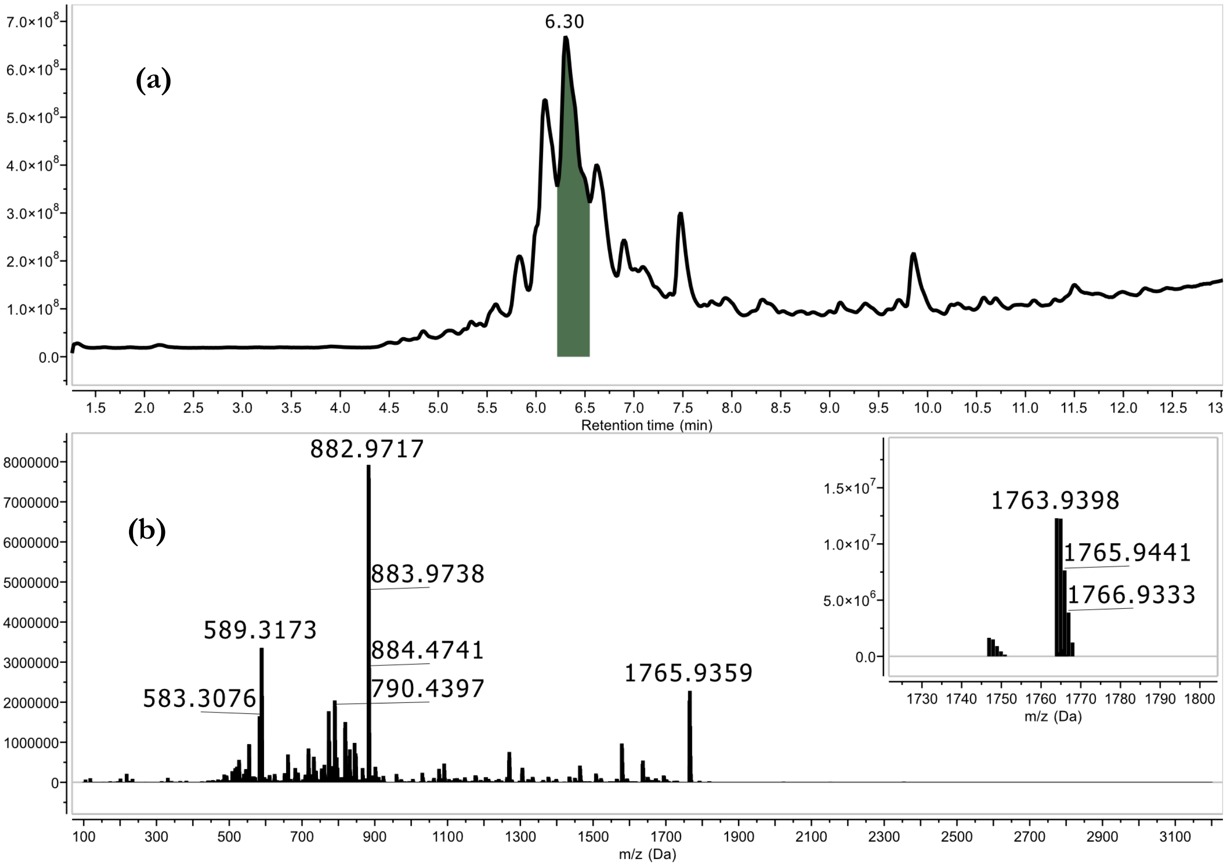

SI Figure 78. LC-HR-ESI-QTOF Profile of crude Barstar[75–90]. (a) TIC chromatogram of Barstar[75–90]; Rt 6.30 min. (b) ESI-TOF spectrum found within Rt 6.30 min (insert: deconvoluted masses). Monoisotopic mass (ESI+) calcd. for $C_{77}H_{129}N_{21}O_{24}S$ 1763.9240, found 1763.9398. LCMS Gradient A (**Section 2.8**).

UHPLC of crude Barstar[75–90]

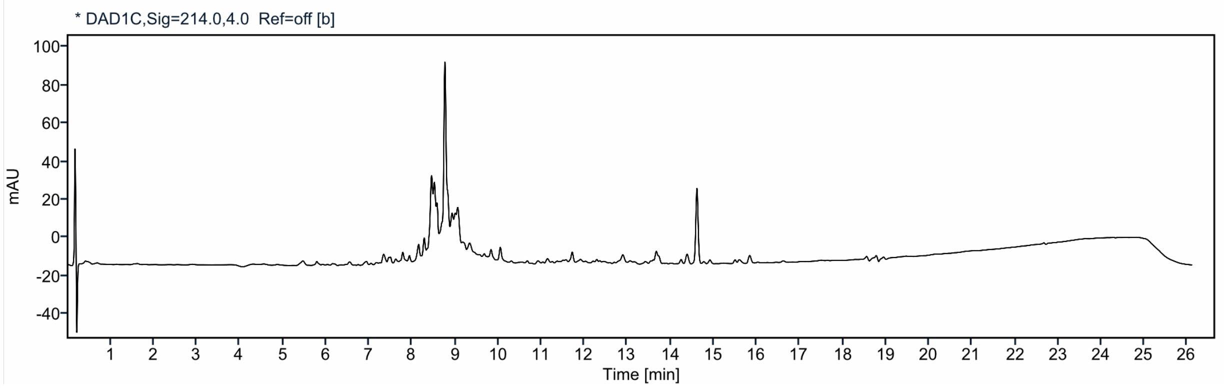

SI Figure 79. UHPLC profile of crude Barstar[75–90]. Rt 8.64 min (Agilent Zorbax 300SB-C18 RRHD column, 1.8 µm, 2.1 × 50 mm, 5–95% MeCN over 20 min, ca. 4.5%B/min), 23% purity based on Area Under Curve (AUC) at λ = 214 nm.

4.12 Rink Amide NovaGel (NovaGel-RAM, 0.54 mmol/g loading): Barstar[75–90]-ArgTag

10 20

H_2N-FREAKAEGCD ITIILS**RRRR RR**-$CONH_2$ (22 AA)

The peptide Barstar[75–90]-ArgTag was synthesized on commercially available Rink Amide NovaGel resin (0.54 mmol/g, 57 mg, 31 µmol) using the standard AFPS protocol (**Section 2.2.2**)

(**SI Figure 80**). Total synthesis time to afford resin-bound Barstar[75–90]-ArgTag was approximately 1 h. Cleavage of the peptidyl-resin (30 mg, approx. 16 µmol) according to the cleavage protocol described in **Section 2.6** afforded the crude peptide as a colorless solid (10.2 mg, mass confirmed by LC-HR-ESI-QTOF [**SI Figure 81**], 40% purity by UHPLC [**SI Figure 82**]).

UV-Vis synthesis trace

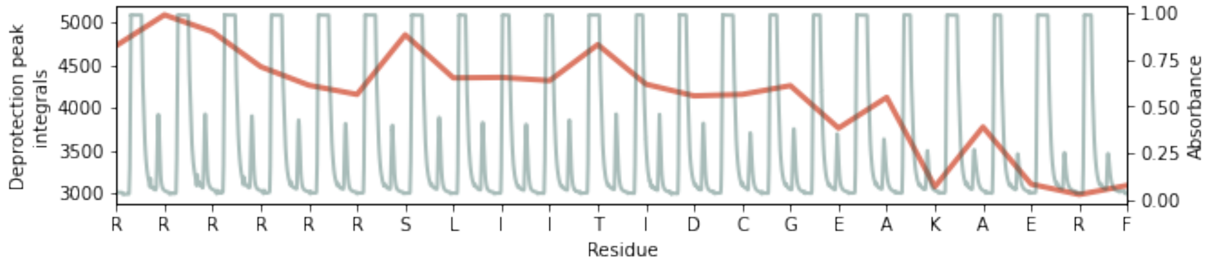

SI Figure 80. UV trace (λ = 310 nm) from AFPS of Barstar[75–90]-ArgTag (green) and deprotection peak integrals (red). Note: UV chromatograms are plotted against time and are not directly aligned with the sequence labels on the x-axis. Only the integrals of the deprotection peaks are aligned with the corresponding amino acid positions.

LC-QTOF-of crude Barstar[75–90]-ArgTag

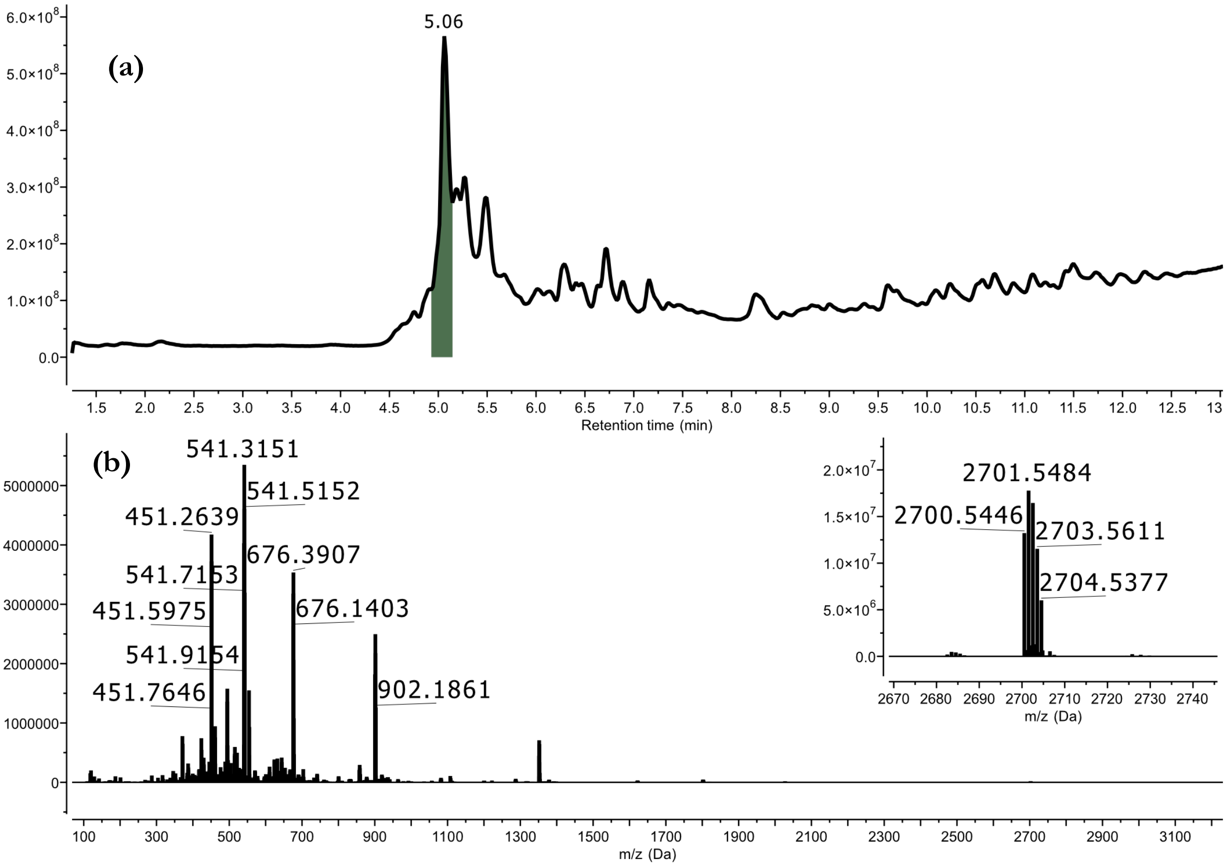

SI Figure 81. LC-HR-ESI-QTOF Profile of crude Barstar[75–90] bearing the ArgTag. (a) TIC chromatogram of Barstar[75–90]-ArgTag; Rt 5.06 min. (b) ESI-TOF spectrum found within Rt 5.06 min (insert: deconvoluted masses). Monoisotopic mass (ESI+) calcd. for $C_{113}H_{201}N_{45}O_{30}S$ 2700.5307, found 2700.5446. LCMS Gradient A (**Section 2.8**).

UHPLC of crude Barstar[75–90]-ArgTag

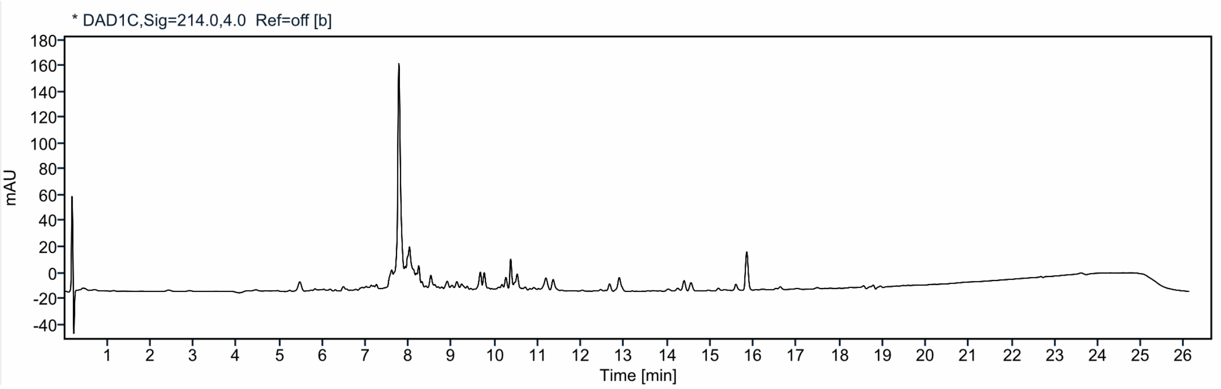

SI Figure 82. UHPLC profile of crude Barstar[75–90] bearing ArgTag. Rt 7.77 min (Agilent Zorbax 300SB-C18 RRHD column, 1.8 µm, 2.1 × 50 mm, 5–95% MeCN over 20 min, ca. 4.5%B/min), 40% purity based on Area Under Curve (AUC) at λ = 214 nm.

4.13 Rink Amide LiQ (LiQ-RAM, 0.21 mmol/g loading): Barstar[75–90]

```
           10         16
H2N-FREAKAEGCD ITIILS-CONH2       (16 AA)
```

The peptide Barstar[75–90] was synthesized on commercially available Rink Amide LiQ resin (0.21 mmol/g, 104.8 mg, 22 µmol), which was manually downloaded according to the procedure described in **Section 2.4,** using the standard AFPS protocol (**Section 2.2.2**) (**SI Figure 83**). Total synthesis time to afford resin-bound Barstar[75–90] was approximately 0.8 h. Cleavage of the peptidyl-resin (41 mg, approx. 8.6 µmol) according to the cleavage protocol described in **Section 2.6** afforded the crude peptide as a colorless solid (7.9 mg, mass confirmed by LC-HR-ESI-QTOF [**SI Figure 84**], 34% purity by UHPLC [**SI Figure 85**]).

UV-Vis synthesis trace

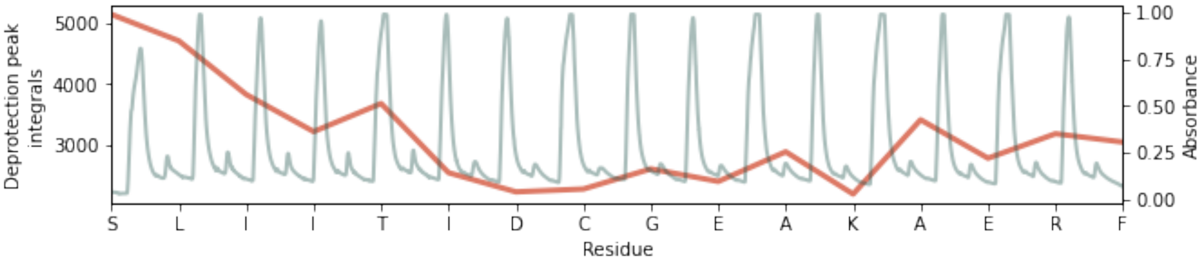

SI Figure 83. UV trace (λ = 310 nm) from AFPS of Barstar[75–90]-ArgTag (green) and deprotection peak integrals (red). Note: UV chromatograms are plotted against time and are not directly aligned with the sequence labels on the x-axis. Only the integrals of the deprotection peaks are aligned with the corresponding amino acid positions.

LC-QTOF of crude Barstar[75–90]

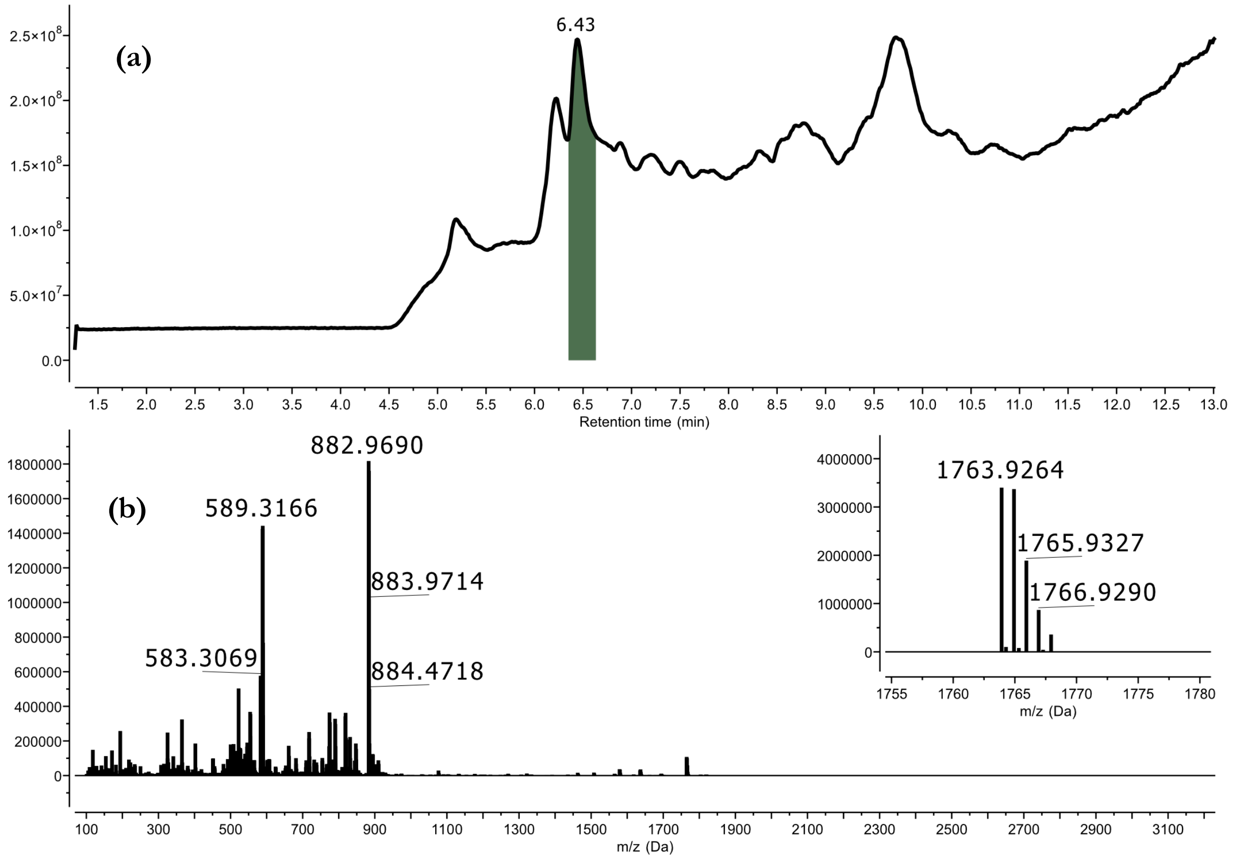

SI Figure 84. LC-HR-ESI-QTOF Profile of crude Barstar[75–90]. (a) TIC chromatogram of Barstar[75–90]; Rt 6.43 min. (b) ESI-TOF spectrum found within Rt 6.43 min (insert deconvoluted masses). Monoisotopic mass (ESI+) calcd. for $C_{77}H_{129}N_{21}O_{24}S$ 1763.9240, found 1763.9264. LCMS Gradient A (**Section 2.8**).

UHPLC of crude Barstar[75–90]

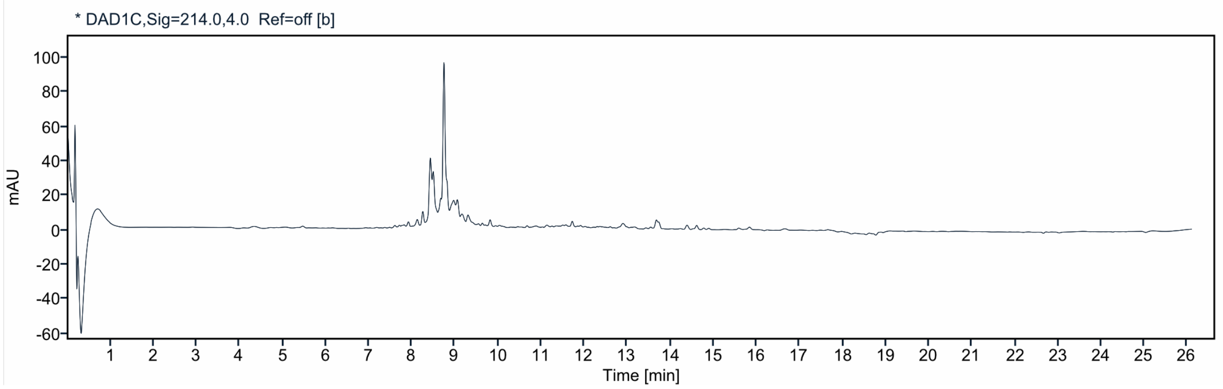

SI Figure 85. UHPLC profile of crude Barstar[75–90]. Rt 8.74 min (Agilent Zorbax 300SB-C18 RRHD column, 1.8 µm, 2.1 × 50 mm, 5–95% MeCN over 20 min, ca. 4.5%B/min), 34% purity based on Area Under Curve (AUC) at λ = 214 nm.

4.14 Rink Amide LiQ (LiQ-RAM, 0.21 mmol/g loading): Barstar[75–90]-ArgTag

```
                    10                  20
H2N-FREAKAEGCD  ITIILSRRRR RR-CONH2          (22 AA)
```

The peptide Barstar[75–90]-ArgTag was synthesized on commercially available Rink Amide LiQ resin (0.21 mmol/g, 104.0 mg, 22 µmol), which was manually downloaded according to the procedure described in **Section 2.4,** using the standard AFPS protocol (**Section 2.2.2**) (**SI Figure 86**). Total synthesis time to afford resin-bound Barstar[75–90]-ArgTag was approximately 1 h. Cleavage of the peptidyl-resin (56 mg, approx. 11.7 µmol) according to the cleavage protocol described in **Section 2.6** afforded the crude peptide as a colorless solid (9.1 mg, mass confirmed by LC-HR-ESI-QTOF [**SI Figure 87**], 64% purity by UHPLC [**SI Figure 88**]).

UV-Vis synthesis trace

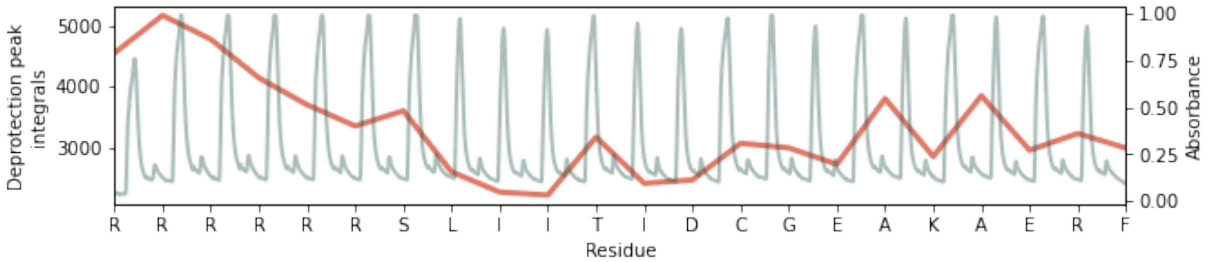

SI Figure 86. UV trace (λ = 310 nm) from AFPS of Barstar[75–90]-ArgTag (green) and deprotection peak integrals (red). Note: UV chromatograms are plotted against time and are not directly aligned with the sequence labels on the x-axis. Only the integrals of the deprotection peaks are aligned with the corresponding amino acid positions.

LC-QTOF of crude Barstar[75–90]-ArgTag

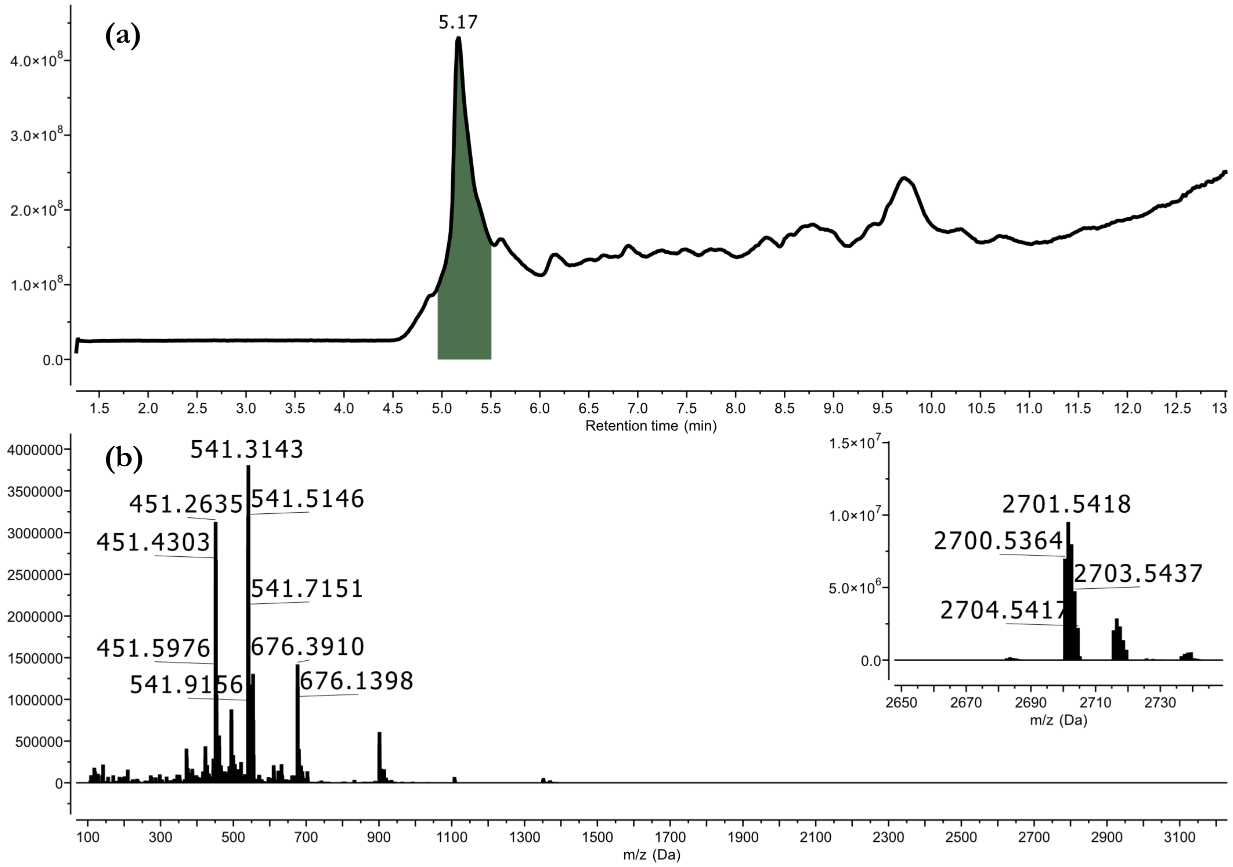

SI Figure 87. LC-HR-ESI-QTOF Profile of crude Barstar[75–90] bearing the ArgTag. (a) TIC chromatogram of Barstar[75–90]-ArgTag; Rt 5.17 min. (b) ESI-TOF spectrum found within Rt 5.17 min (insert: deconvoluted masses). Monoisotopic mass (ESI+) calcd. for $C_{113}H_{201}N_{45}O_{30}S$ 2700.5307, found 2700.5364. LCMS Gradient A (**Section 2.8**).

UHPLC of crude Barstar[75–90]-ArgTag

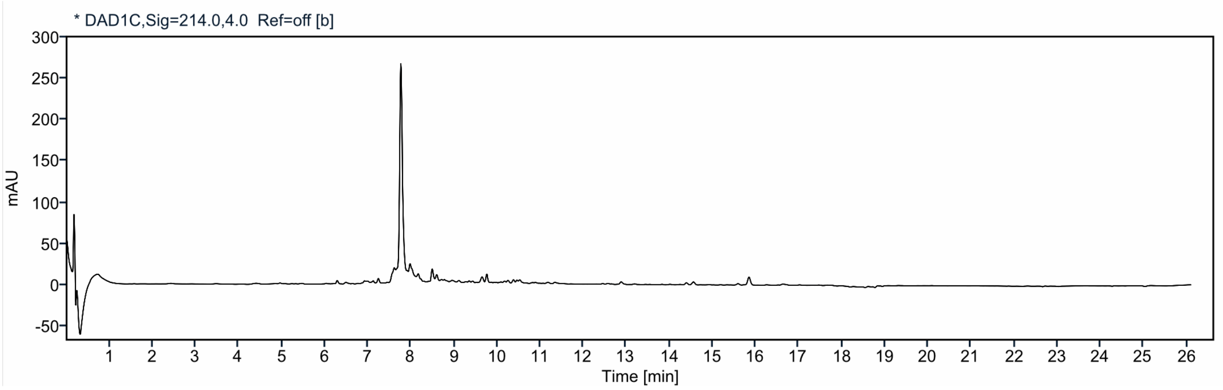

SI Figure 88. UHPLC profile of crude Barstar[75–90] bearing ArgTag. Rt 7.76 min (Agilent Zorbax 300SB-C18 RRHD column, 1.8 µm, 2.1 × 50 mm, 5–95% MeCN over 20 min, ca. 4.5%B/min), 64% purity based on Area Under Curve (AUC) at λ = 214 nm.

4.15 Rink Amide LiQ (LiQ-RAM, 0.50 mmol/g loading): Barstar[75–90]

```
                   10        16
  H2N-FREAKAEGCD ITIILS-CONH2       (16 AA)
```

The peptide Barstar[75–90] was synthesized on commercially available Rink Amide LiQ resin (0.50 mmol/g, 101.4 mg, 51 µmol) using the standard AFPS protocol (**Section 2.2.2**) (**SI Figure 89**). Total synthesis time to afford resin-bound Barstar[75–90] was approximately 0.8 h. Cleavage of the peptidyl-resin (32 mg, approx. 16 µmol) according to the cleavage protocol described in **Section 2.6** afforded the crude peptide as a colorless solid (11.2 mg, mass confirmed by LC-HR-ESI-QTOF [**SI Figure 90**], 30% purity by UHPLC [**SI Figure 91**]).

UV-Vis synthesis trace

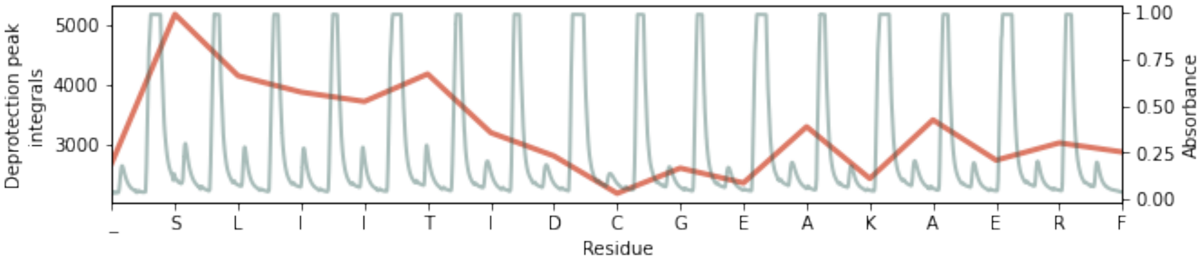

SI Figure 89. UV trace (λ = 310 nm) from AFPS of Barstar[75–90] (green) and deprotection peak integrals (red). Note: UV chromatograms are plotted against time and are not directly aligned with the sequence labels on the x-axis. Only the integrals of the deprotection peaks are aligned with the corresponding amino acid positions.

LC-QTOF of crude Barstar[75–90]

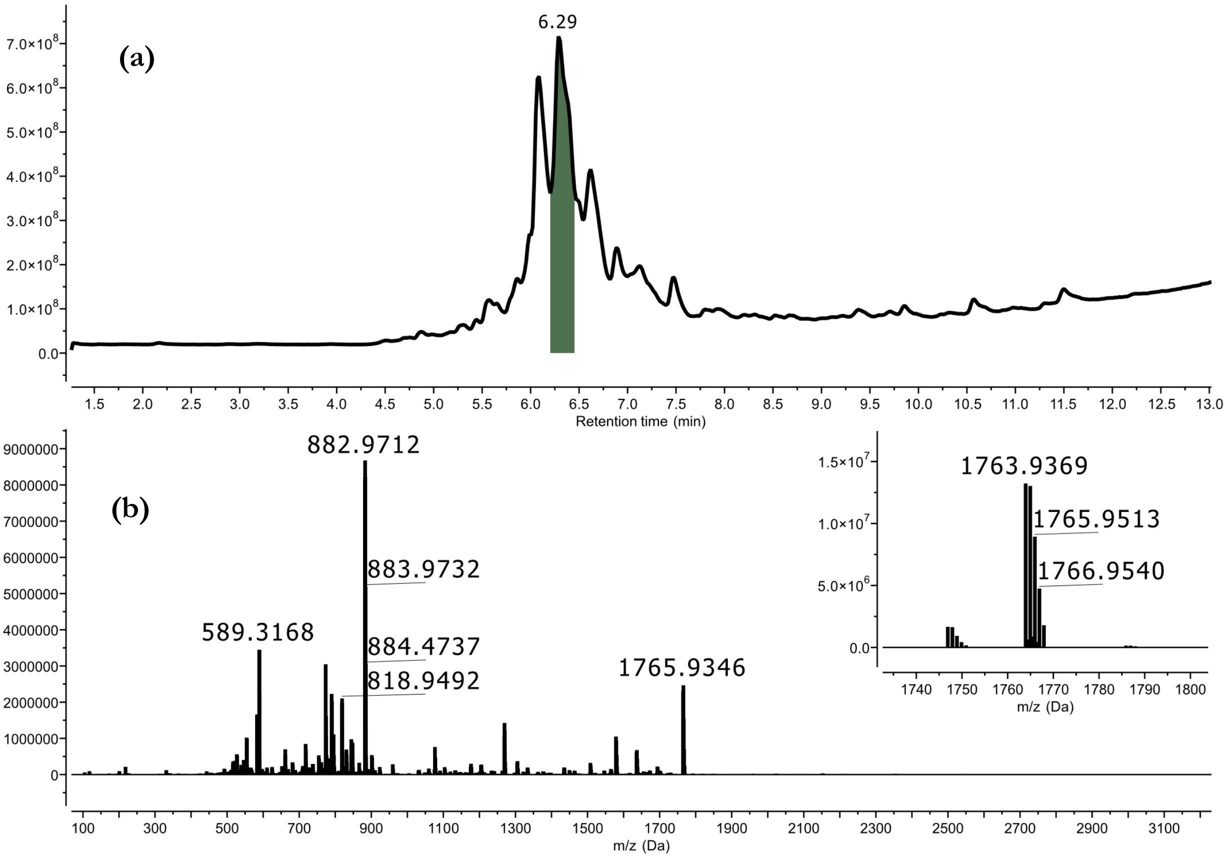

SI Figure 90. LC-HR-ESI-QTOF Profile of crude Barstar[75–90]. (a) TIC chromatogram of Barstar[75–90]; Rt 6.29 min. (b) ESI-TOF spectrum found within Rt 6.29 min (insert deconvoluted masses). Monoisotopic mass (ESI+) calcd. for $C_{77}H_{129}N_{21}O_{24}S$ 1763.9240, found 1763.9369. LCMS Gradient A (**Section 2.8**).

UHPLC of crude Barstar[75–90]

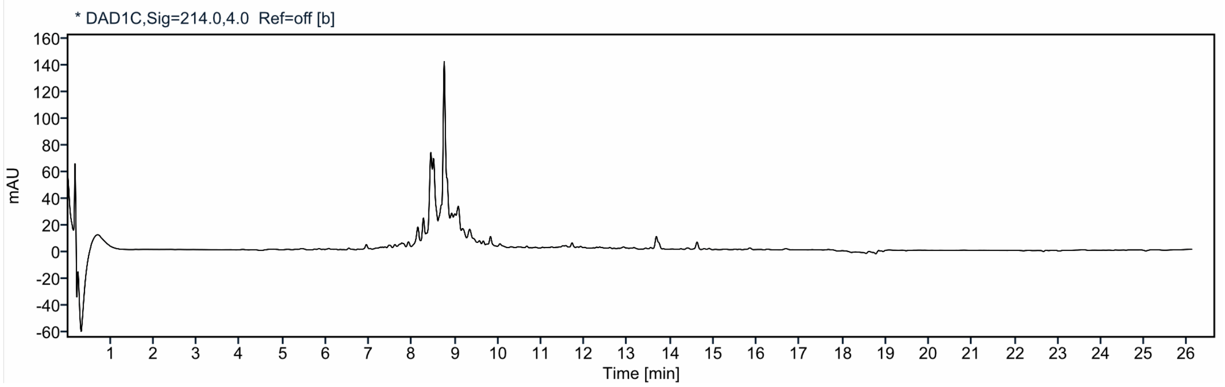

SI Figure 91. UHPLC profile of crude Barstar[75–90]. Rt 8.75 min (Agilent Zorbax 300SB-C18 RRHD column, 1.8 µm, 2.1 × 50 mm, 5–95% MeCN over 20 min, ca. 4.5%B/min), 30% purity based on Area Under Curve (AUC) at λ = 214 nm.

4.16 Rink Amide LiQ (LiQ-RAM, 0.50 mmol/g loading): Barstar[75–90]-ArgTag

10 20
H_2N-FREAKAEGCD ITIILS**RRRR RR**-$CONH_2$ (22 AA)

The peptide Barstar[75–90]-ArgTag was synthesized on commercially available Rink Amide LiQ resin (0.50 mmol/g, 101.6 mg, 51 µmol) using the standard AFPS protocol (**Section 2.2.2**) (**SI Figure 92**). Total synthesis time to afford resin-bound Barstar[75–90]-ArgTag was approximately 1 h. Cleavage of the peptidyl-resin (42 mg, approx. 21 µmol) according to the cleavage protocol described in **Section 2.6** afforded the crude peptide as a colorless solid (13.3 mg, mass confirmed by LC-HR-ESI-QTOF [**SI Figure 93**], 68% purity by UHPLC [**SI Figure 94**]).

UV-Vis synthesis trace

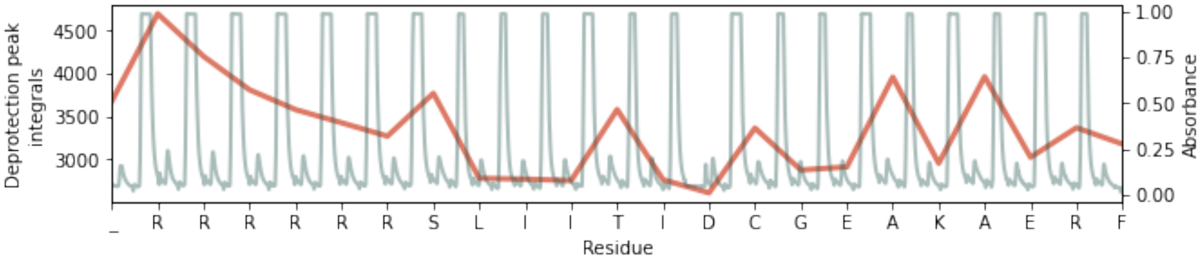

SI Figure 92. UV trace (λ = 310 nm) from AFPS of Barstar[75–90]-ArgTag (green) and deprotection peak integrals (red). Note: UV chromatograms are plotted against time and are not directly aligned with the sequence labels on the x-axis. Only the integrals of the deprotection peaks are aligned with the corresponding amino acid positions.

LC-QTOF of crude Barstar[75–90]-ArgTag

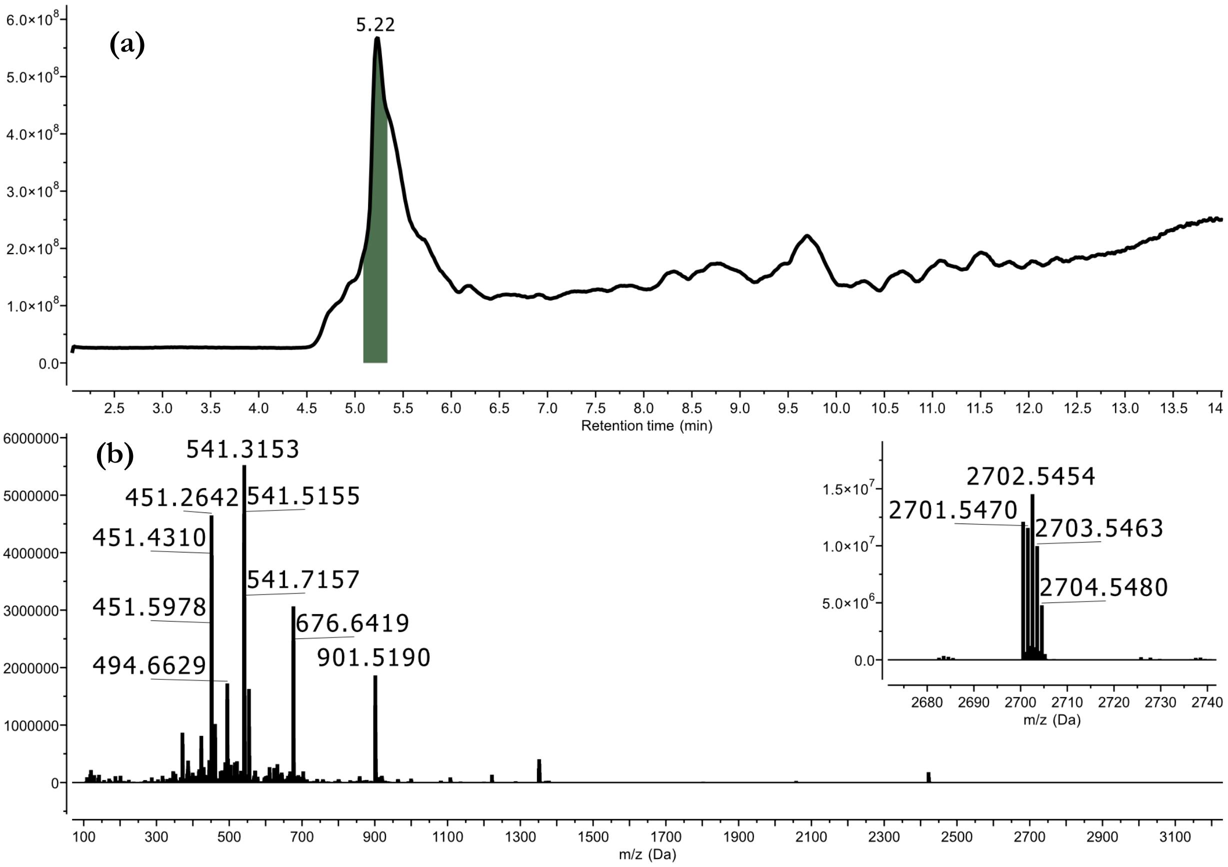

SI Figure 93. LC-HR-ESI-QTOF Profile of crude Barstar[75–90] bearing the ArgTag. (a) TIC chromatogram of Barstar[75–90]-ArgTag; Rt 5.22 min. (b) ESI-TOF spectrum found within Rt 5.22 min (insert: deconvoluted masses). Monoisotopic mass (ESI+) calcd. for $C_{113}H_{201}N_{45}O_{30}S$ 2700.5307, found 2700.5424. LCMS Gradient A (**Section 2.8**).

UHPLC of crude Barstar[75–90]-ArgTag

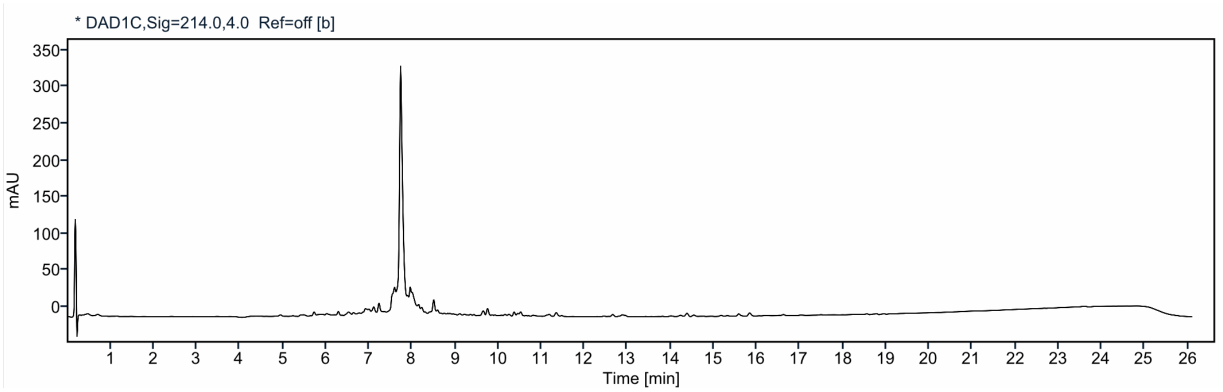

SI Figure 94. UHPLC profile of crude Barstar[75–90] bearing ArgTag. Rt 7.73 min (Agilent Zorbax 300SB-C18 RRHD column, 1.8 µm, 2.1 × 50 mm, 5–95% MeCN over 20 min, ca. 4.5%B/min), 68% purity based on Area Under Curve (AUC) at λ = 214 nm.

4.17 Rink Amide NovaPEG (NovaPEG-RAM, 0.20 mmol/g loading): Barstar[75–90]

```
            10         16
H2N-FREAKAEGCD ITIILS-CONH2      (16 AA)
```

The peptide Barstar[75–90] was synthesized on commercially available Rink Amide NovaPEG resin (0.20 mmol/g, 103.9 mg, 21 µmol) using the standard AFPS protocol (**Section 2.2.2**) (**SI Figure 95**). Total synthesis time to afford resin-bound Barstar[75–90] was approximately 0.8 h. Cleavage of the peptidyl-resin (40 mg, approx. 8 µmol) according to the cleavage protocol described in **Section 2.6** afforded the crude peptide as a colorless solid (6.1 mg, mass confirmed by LC-HR-ESI-QTOF [**SI Figure 96**], 51% purity by UHPLC [**SI Figure 97**]).

UV-Vis synthesis trace

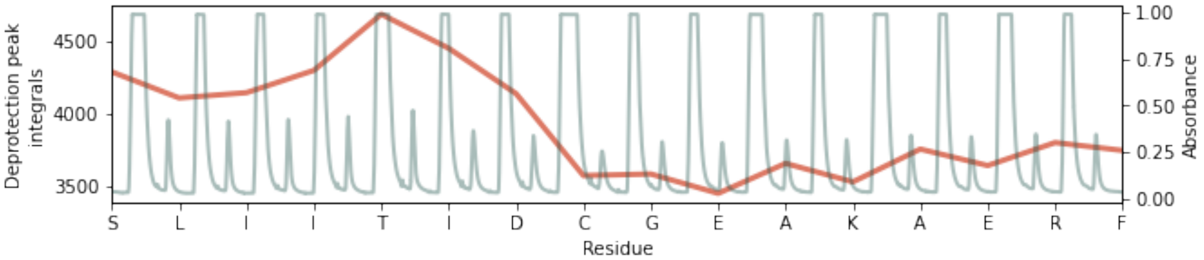

SI Figure 95. UV trace (λ = 310 nm) from AFPS of Barstar[75–90] (green) and deprotection peak integrals (red). Note: UV chromatograms are plotted against time and are not directly aligned with the sequence labels on the x-axis. Only the integrals of the deprotection peaks are aligned with the corresponding amino acid positions.

LC-QTOF of crude Barstar[75–90]

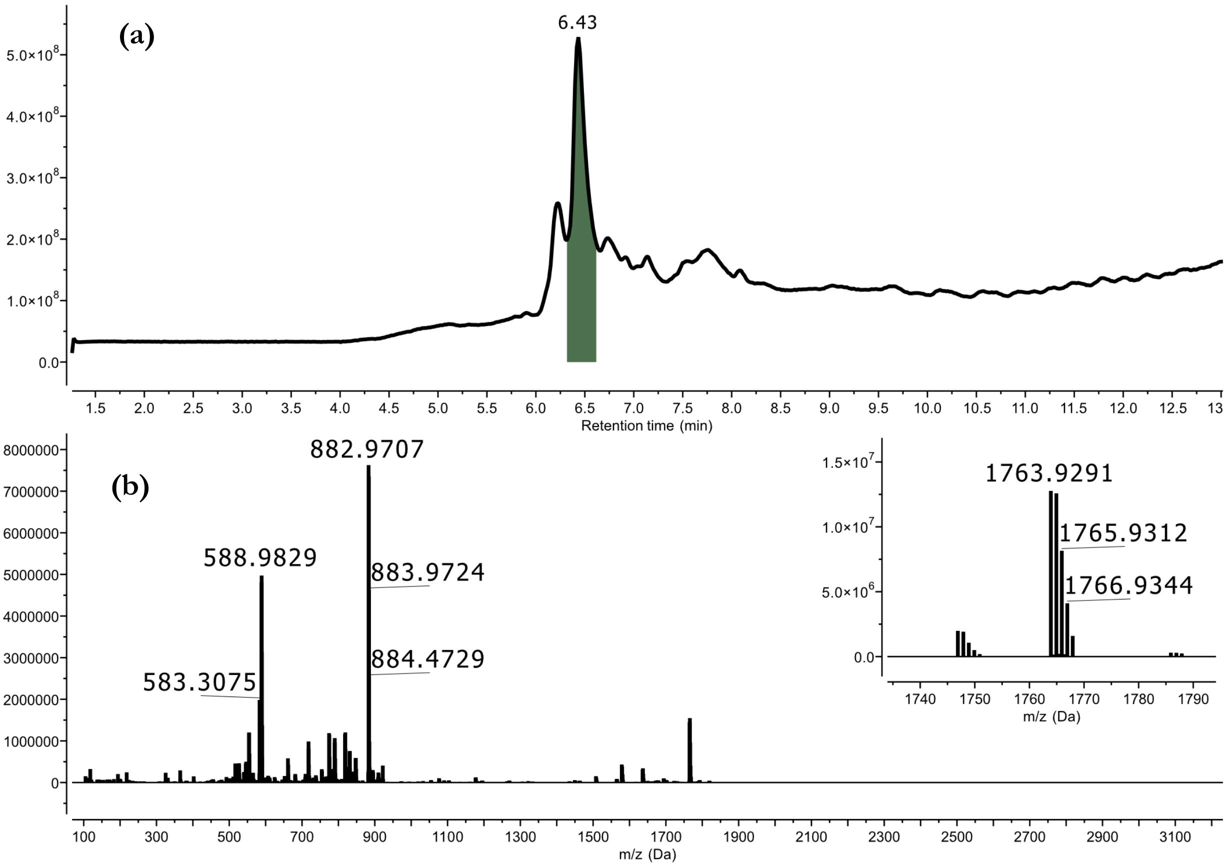

SI Figure 96. LC-HR-ESI-QTOF Profile of crude Barstar[75–90]. (a) TIC chromatogram of Barstar[75–90]; Rt 6.43 min. (b) ESI-TOF spectrum found within Rt 6.43 min (insert deconvoluted masses). Monoisotopic mass (ESI+) calcd. for $C_{77}H_{129}N_{21}O_{24}S$ 1763.9240, found 1763.9291. LCMS Gradient A (**Section 2.8**).

UHPLC of crude Barstar[75–90]

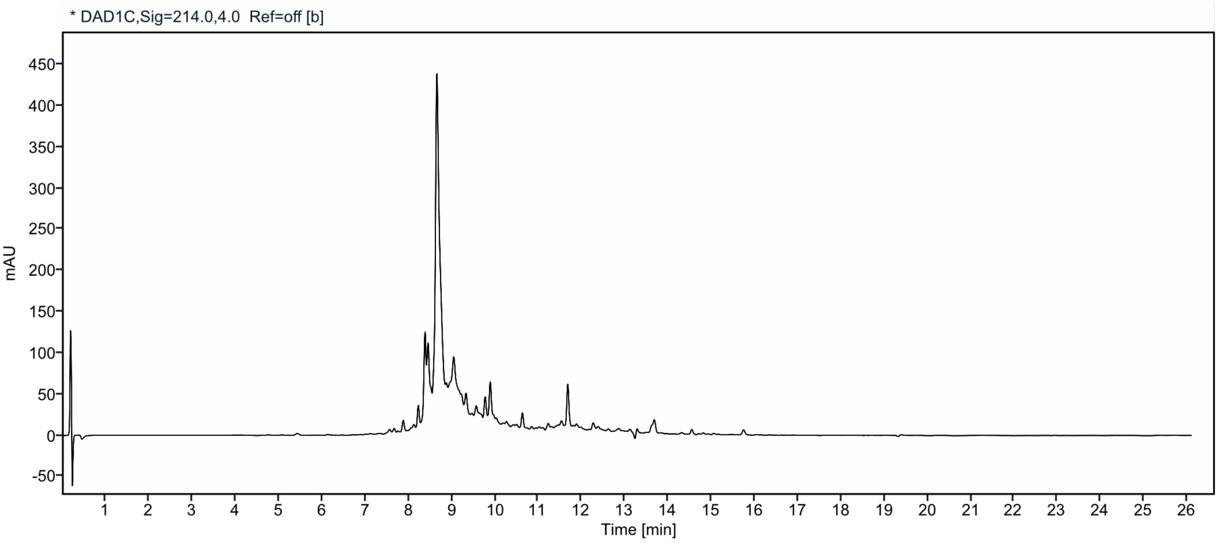

SI Figure 97. UHPLC profile of crude Barstar[75–90]. Rt 8.65 min (Agilent Zorbax 300SB-C18 RRHD column, 1.8 µm, 2.1 × 50 mm, 5–95% MeCN over 20 min, ca. 4.5%B/min), 51% purity based on Area Under Curve (AUC) at λ = 214 nm.

4.18 **Rink Amide NovaPEG (NovaPEG-RAM, 0.20 mmol/g loading):** Barstar[75–90]-ArgTag

```
                    10           20
H2N-FREAKAEGCD ITIILSRRRR RR-CONH2       (22 AA)
```

The peptide Barstar[75–90]-ArgTag was synthesized on commercially available Rink Amide NovaPEG resin (0.20 mmol/g, 102.6 mg, 21 μmol) using the standard AFPS protocol (**Section 2.2.2**) (**SI Figure 98**). Total synthesis time to afford resin-bound Barstar[75–90]-ArgTag was approximately 1 h. Cleavage of the peptidyl-resin (49 mg, approx. 10 μmol) according to the cleavage protocol described in **Section 2.6** afforded the crude peptide as a colorless solid (7.3 mg, mass confirmed by LC-HR-ESI-QTOF [**SI Figure 99**], 64% purity by UHPLC [**SI Figure 100**]).

UV-Vis synthesis trace

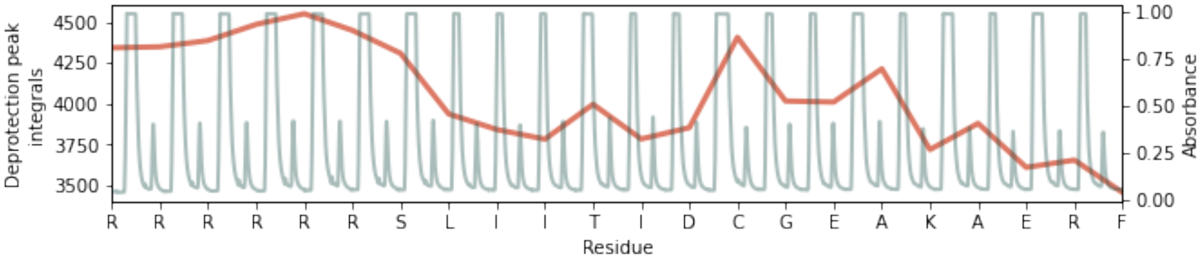

SI Figure 98. UV trace (λ = 310 nm) from AFPS of Barstar[75–90]-ArgTag (green) and deprotection peak integrals (red). Note: UV chromatograms are plotted against time and are not directly aligned with the sequence labels on the x-axis. Only the integrals of the deprotection peaks are aligned with the corresponding amino acid positions.

LC-QTOF of crude Barstar[75–90]-ArgTag

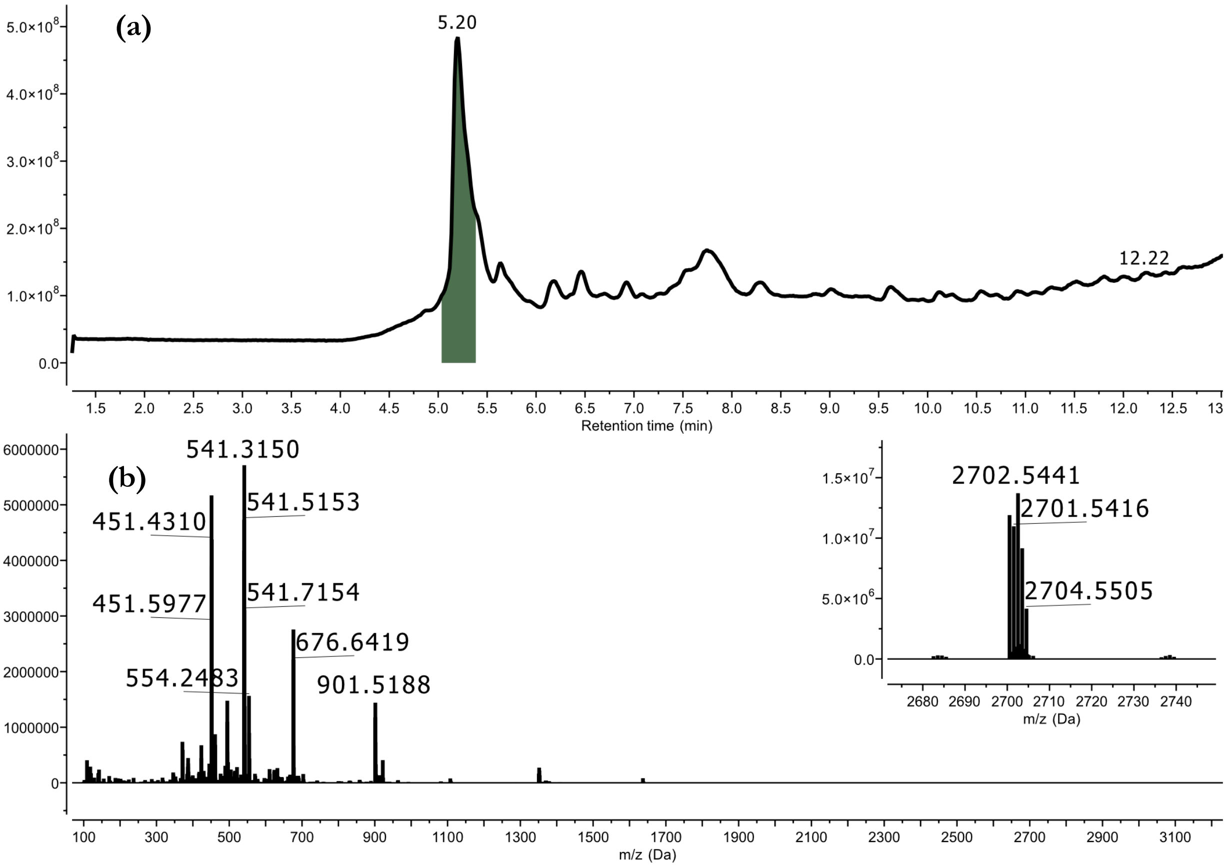

SI Figure 99. LC-HR-ESI-QTOF Profile of crude Barstar[75–90] bearing the ArgTag. (**a**) TIC chromatogram of Barstar[75–90]-ArgTag; Rt 5.20 min. (**b**) ESI-TOF spectrum found within Rt 5.20 min (insert: deconvoluted masses). Monoisotopic mass (ESI+) calcd. for $C_{113}H_{201}N_{45}O_{30}S$ 2700.5307, found 2700.5387. LCMS Gradient A (**Section 2.8**).

UHPLC of crude Barstar[75–90]-ArgTag

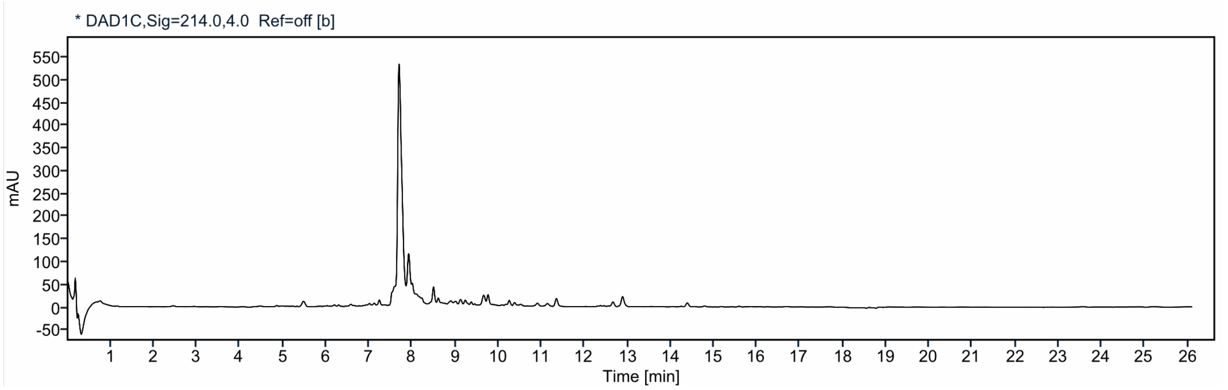

SI Figure 100. UHPLC profile of crude Barstar[75–90] bearing ArgTag. Rt 7.69 min (Agilent Zorbax 300SB-C18 RRHD column, 1.8 µm, 2.1 × 50 mm, 5–95% MeCN over 20 min, ca. 4.5%B/min), 64% purity based on Area Under Curve (AUC) at λ = 214 nm.

4.19 Rink Amide NovaPEG (NovaPEG-RAM, 0.41 mmol/g loading): Barstar[75–90]

```
          10        16
H2N-FREAKAEGCD ITIILS-CONH2      (16 AA)
```

The peptide Barstar[75–90] was synthesized on commercially available Rink Amide NovaPEG resin (0.41 mmol/g, 149.9 mg, 61 µmol) using the standard AFPS protocol (**Section 2.2.2**) (**SI Figure**

101). Total synthesis time to afford resin-bound Barstar[75–90] was approximately 0.8 h. Cleavage of the peptidyl-resin (49 mg, approx. 20 μmol) according to the cleavage protocol described in **Section 2.6** afforded the crude peptide as a colorless solid (12.4 mg, mass confirmed by LC-HR-ESI-QTOF [**SI Figure 102**], 26% purity by UHPLC [**SI Figure 103**]).

UV-Vis synthesis trace

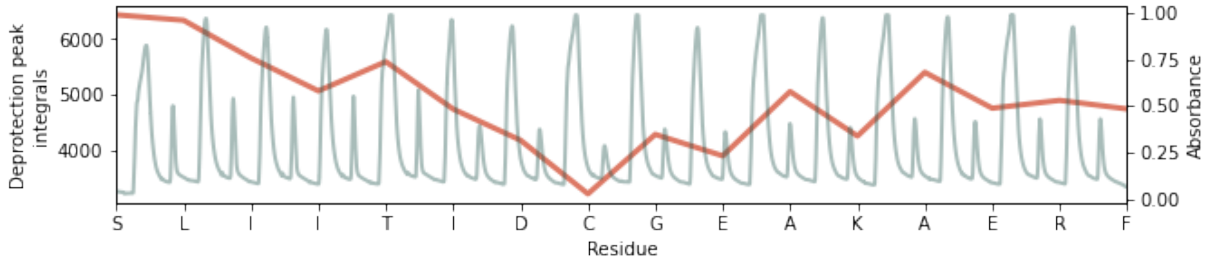

SI Figure 101. UV trace (λ = 310 nm) from AFPS of Barstar[75–90] (green) and deprotection peak integrals (red). Note: UV chromatograms are plotted against time and are not directly aligned with the sequence labels on the x-axis. Only the integrals of the deprotection peaks are aligned with the corresponding amino acid positions.

LC-QTOF of crude Barstar[75–90]

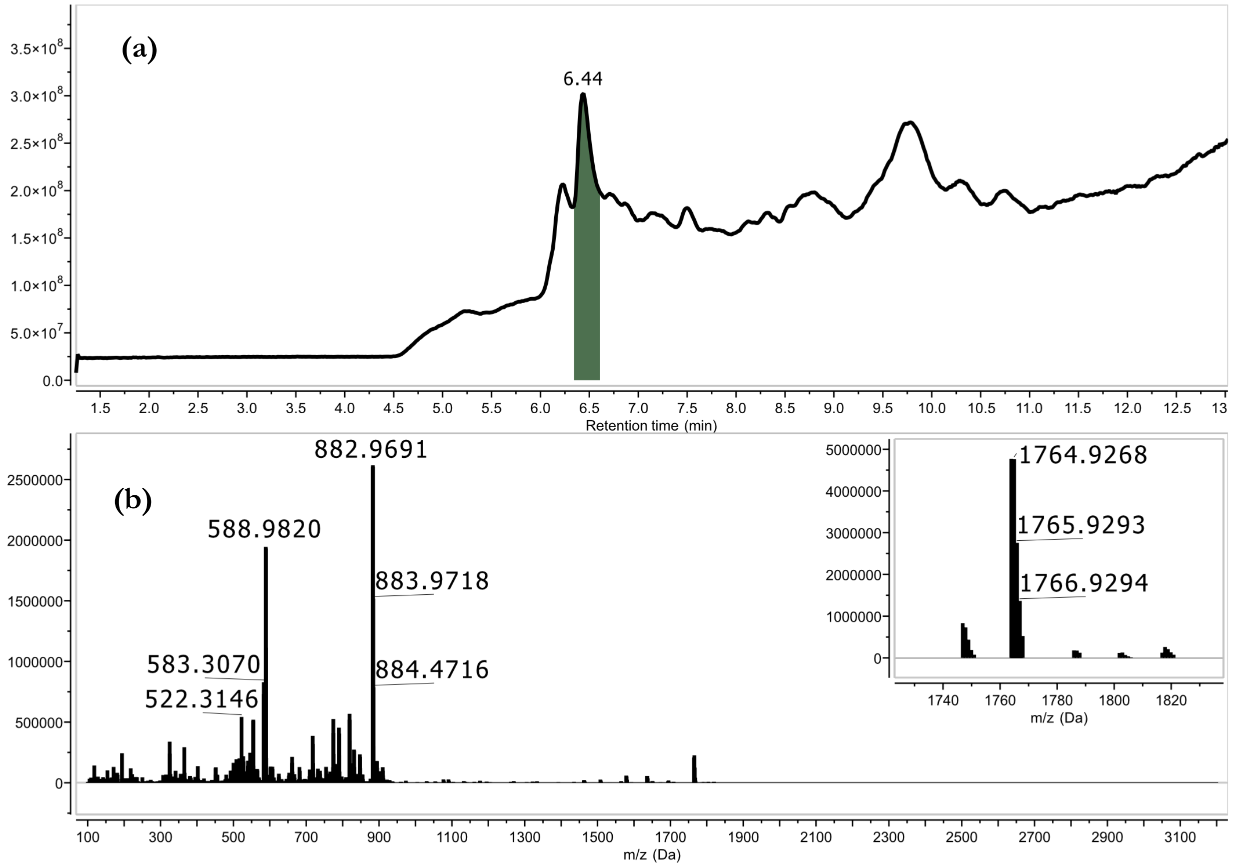

SI Figure 102. LC-HR-ESI-QTOF Profile of crude Barstar[75–90]. (a) TIC chromatogram of Barstar[75–90]; Rt 6.44 min. (b) ESI-TOF spectrum found within Rt 6.44 min (insert deconvoluted masses). Monoisotopic mass (ESI+) calcd. for $C_{77}H_{129}N_{21}O_{24}S$ 1763.9240, found 1763.9238. LCMS Gradient A (**Section 2.8**).

UHPLC of crude Barstar[75–90]

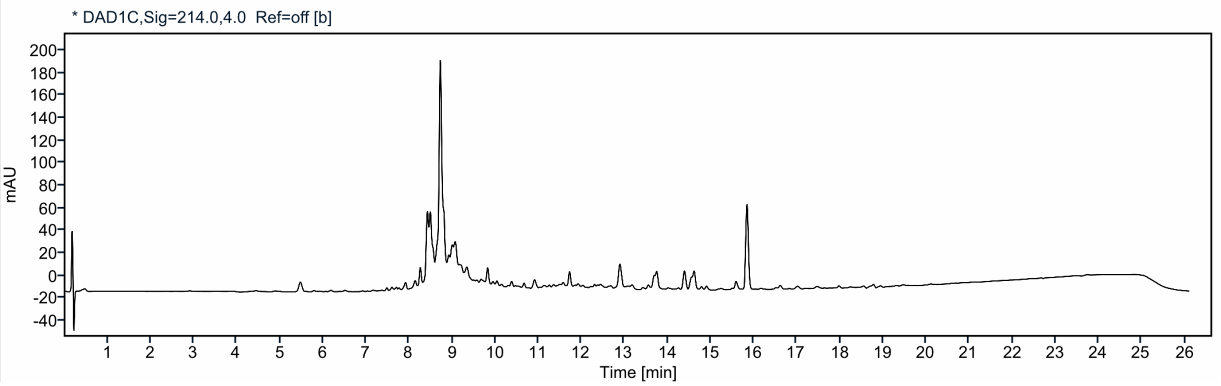

SI Figure 103. UHPLC profile of crude Barstar[75–90]. Rt 8.72 min (Agilent Zorbax 300SB-C18 RRHD column, 1.8 µm, 2.1 × 50 mm, 5–95% MeCN over 20 min, ca. 4.5%B/min), 26% purity based on Area Under Curve (AUC) at λ = 214 nm.

4.20 Rink Amide NovaPEG (NovaPEG-RAM, 0.41 mmol/g loading): Barstar[75–90]

```
                    10         20
H2N-FREAKAEGCD ITIILSRRRR RR-CONH2        (22 AA)
```

The peptide Barstar[75–90]-ArgTag was synthesized on commercially available Rink Amide NovaPEG resin (0.41 mmol/g, 104.1 mg, 43 µmol) using the standard AFPS protocol (**Section 2.2.2**) (**SI Figure 104**). Total synthesis time to afford resin-bound Barstar[75–90]-ArgTag was approximately 1 h. Cleavage of the peptidyl-resin (35 mg, approx. 14 µmol) according to the cleavage protocol described in **Section 2.6** afforded the crude peptide as a colorless solid (10.2 mg, mass confirmed by LC-HR-ESI-QTOF [**SI Figure 105**], 68% purity by UHPLC [**SI Figure 106**]).

UV-Vis synthesis trace

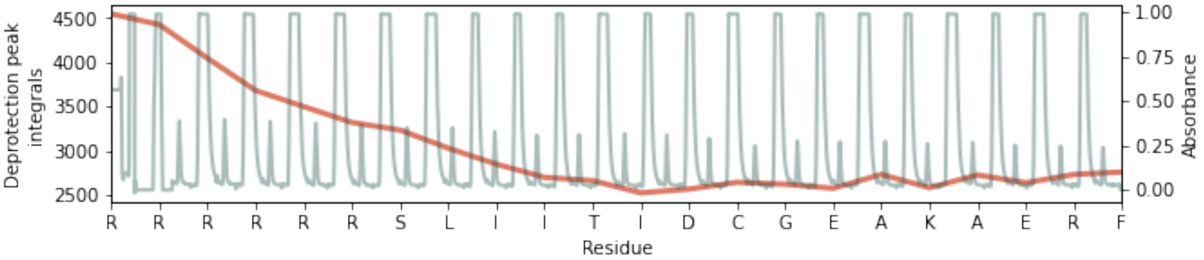

SI Figure 104. UV trace (λ = 310 nm) from AFPS of Barstar[75–90]-ArgTag (green) and deprotection peak integrals (red). Note: UV chromatograms are plotted against time and are not directly aligned with the sequence labels on the x-axis. Only the integrals of the deprotection peaks are aligned with the corresponding amino acid positions.

LC-QTOF of crude Barstar[75–90]-ArgTag

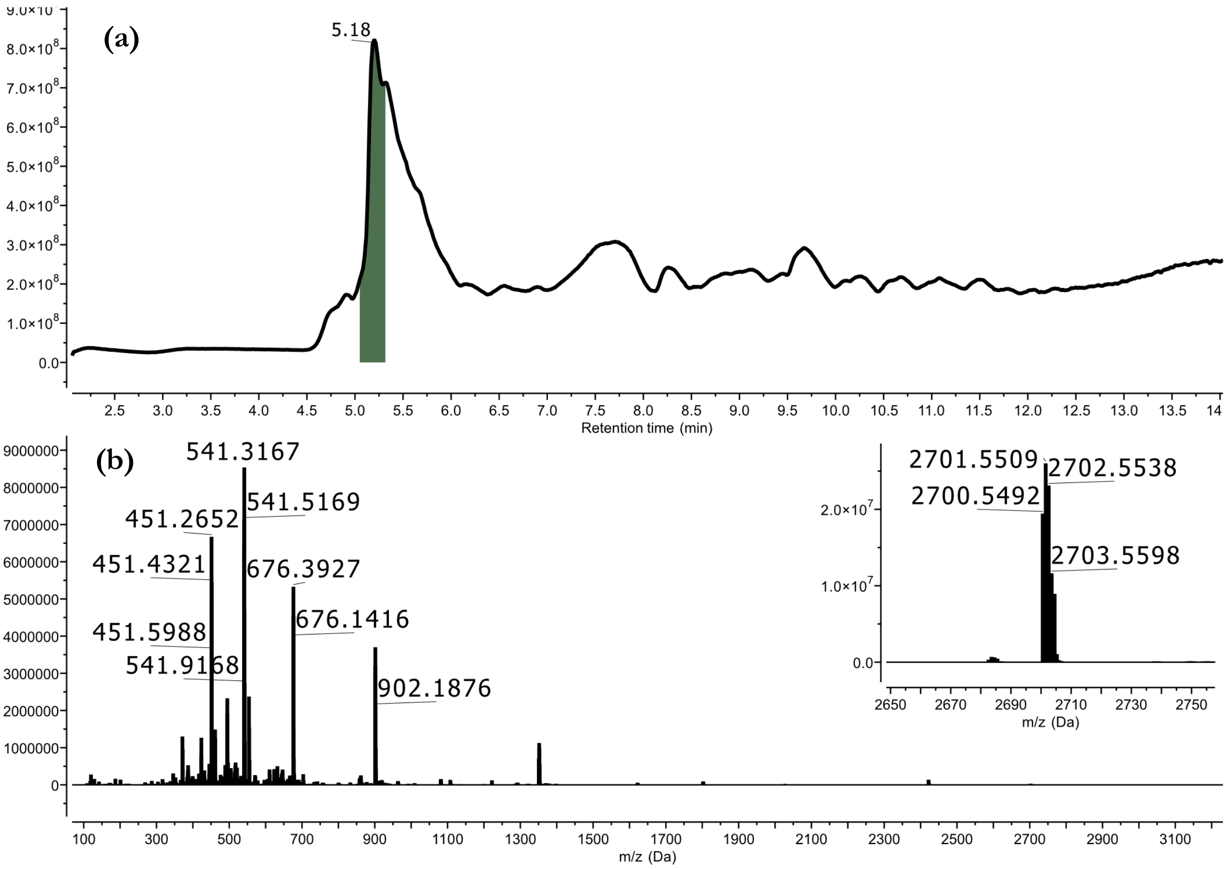

SI Figure 105. LC-HR-ESI-QTOF Profile of crude Barstar[75–90] bearing the ArgTag. (a) TIC chromatogram of Barstar[75–90]-ArgTag; Rt 5.18 min. (b) ESI-TOF spectrum found within Rt 5.18 min (insert: deconvoluted masses). Monoisotopic mass (ESI+) calcd. for $C_{113}H_{201}N_{45}O_{30}S$ 2700.5307, found 2700.5492. LCMS Gradient A (**Section 2.8**).

UHPLC of crude Barstar[75–90]-ArgTag

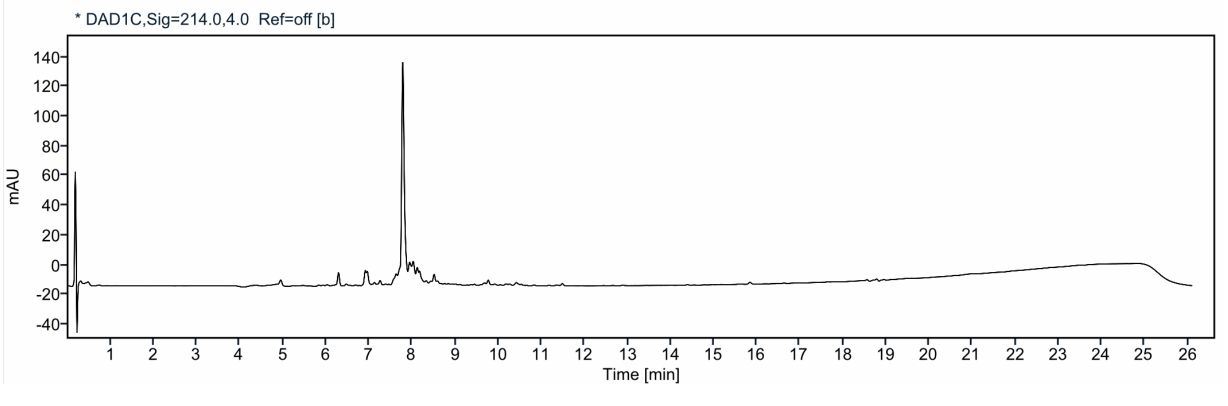

SI Figure 106. UHPLC profile of crude Barstar[75–90] bearing ArgTag. Rt 7.78 min (Agilent Zorbax 300SB-C18 RRHD column, 1.8 µm, 2.1 × 50 mm, 5–95% MeCN over 20 min, ca. 4.5%B/min), 68% purity based on Area Under Curve (AUC) at λ = 214 nm.

5 Evaluation of ArgTag on PeptiPilot system

5.1 2-CTC polystyrene (PS-2 CTC, 0.75 mmol/g loading): Barstar[75–90]

```
              10         16
H2N-FREAKAEGCD ITIILS-COOH        (16 AA)
```

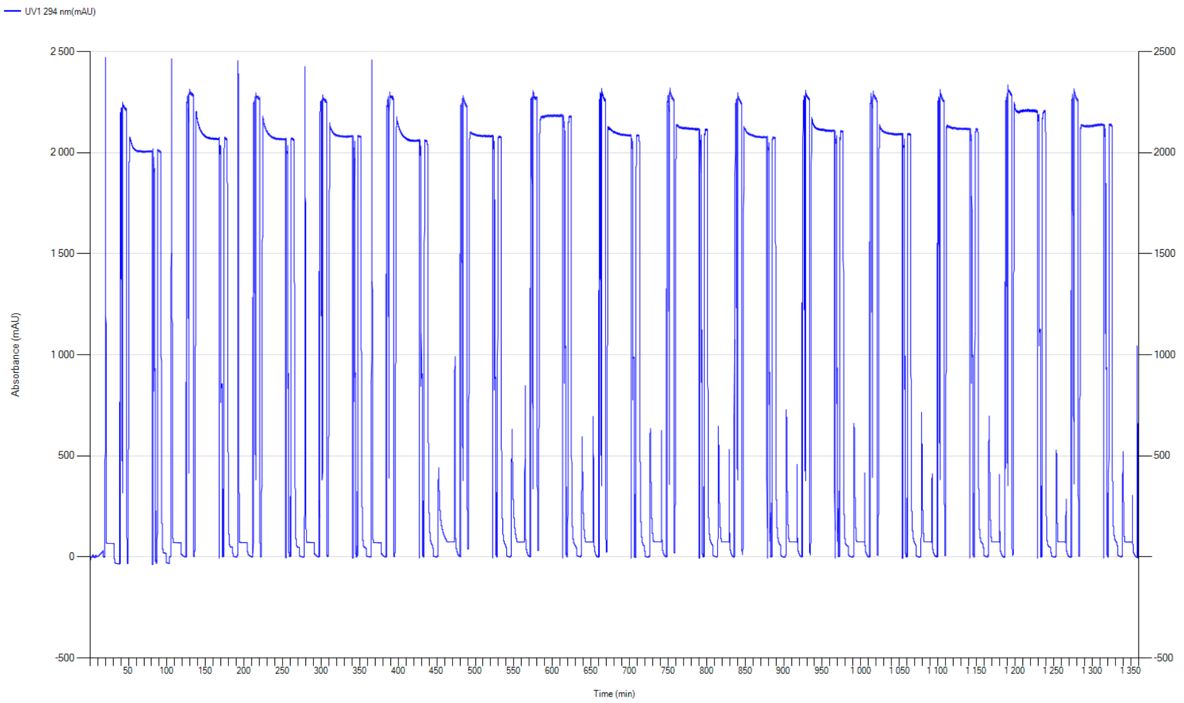

SI Figure 107. Run curve from PeptiPilot at 294 nm. Synthesis of Barstar[75–90].

The peptide Barstar[75–90] was synthesized on commercially available 2-CTC polystyrene resin onto which the Fmoc-Ser(tBu)-OH was manually pre-loaded. To pre-load, the resin (1.2 g) was washed with DMF (3 × 5 mL) and then swollen for 10 min. Neat DIPEA (1 mL) was added to the stock solution of Fmoc-Ser(tBu)-OH (0.4 M, 7 mL) in DMF, stirred for 1 min and then transferred to the swollen resin. The mixture was gently agitated for 1 h, then the resin was filtered off and washed with DMF (3 × 5 mL). This step was then repeated once before the resin was finally washed with DMF (3 × 5 mL), then DCM (3 × 5 mL). The resin dried under a flow of air and then under vacuum. The loading of the resin was determined according to **Section 2.5** to be 0.75 mmol/g. The synthesis of resin-bound Barstar[75–90] was performed according to **Section 2.2.3** and total synthesis time was approximately 19 h (**SI Figure 107**). Cleavage of the peptidyl-resin (71.3 mg, approx. 64 μmol) according to the cleavage protocol described in **Section 2.6** afforded the crude peptide as a colorless solid (56.5 mg, mass confirmed by LC-HR-ESI-QTOF [**SI Figure 108**], 27% purity by UHPLC [**SI Figure 109**]).

LC-QTOF of crude Barstar[75–90]

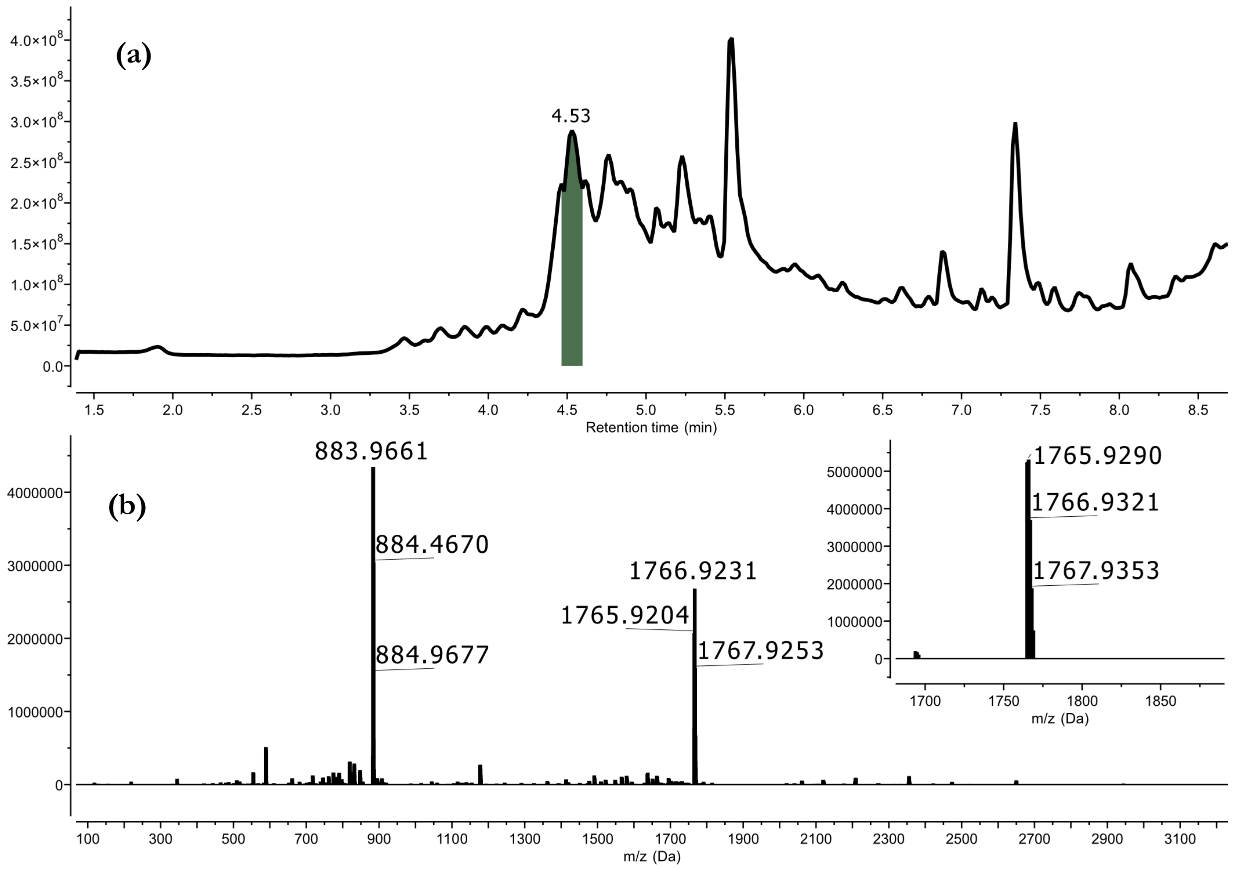

SI Figure 108. LC-HR-ESI-QTOF Profile of crude Barstar[75–90]. (a) TIC chromatogram of Barstar[75–90]; Rt 4.53 min. (b) ESI-TOF spectrum found within Rt 4.53 min (insert deconvoluted masses). Monoisotopic mass (ESI+) calcd. for $C_{77}H_{128}N_{20}O_{25}S$ 1764.9080, found 1764.9243. LCMS Gradient A (**Section 2.8**, different retention time because of new Agilent RRHD C18 column, length 10 cm).

UHPLC of crude Barstar[75–90]

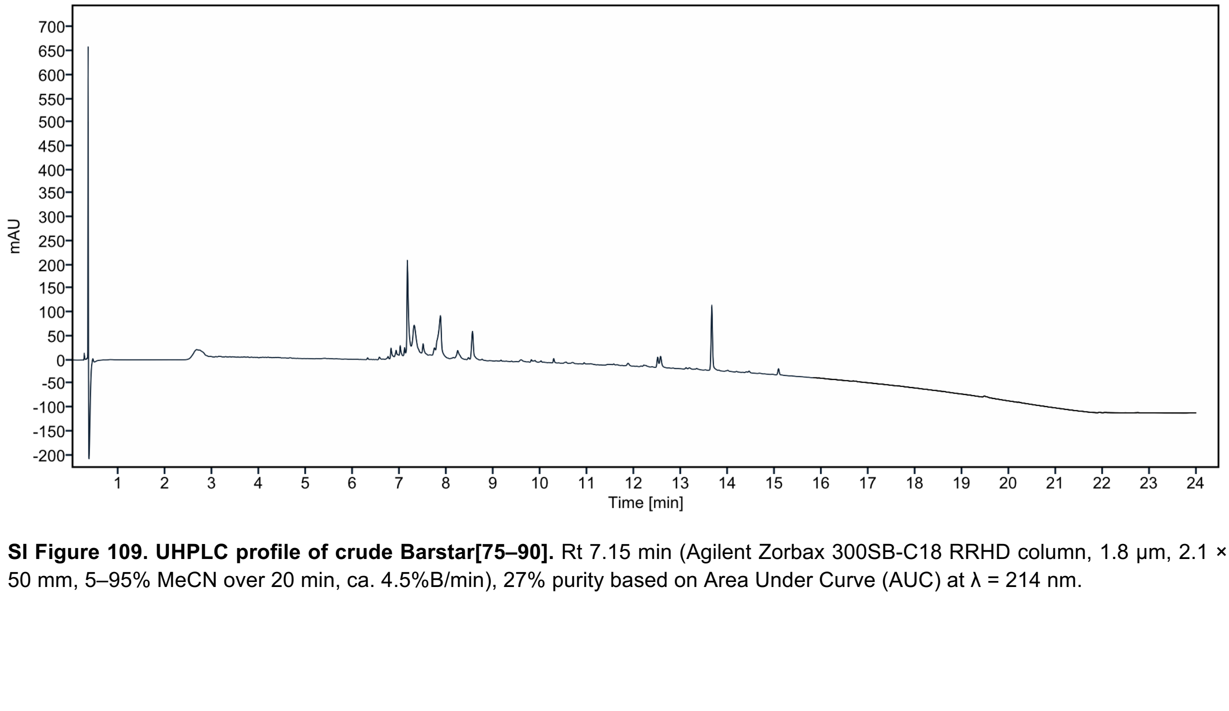

SI Figure 109. UHPLC profile of crude Barstar[75–90]. Rt 7.15 min (Agilent Zorbax 300SB-C18 RRHD column, 1.8 µm, 2.1 × 50 mm, 5–95% MeCN over 20 min, ca. 4.5%B/min), 27% purity based on Area Under Curve (AUC) at λ = 214 nm.

5.2 2-CTC polystyrene (PS-2 CTC, 0.69 mmol/g loading): Barstar[75–90]-ArgTag

```
                 10         20
H2N-FREAKAEGCD ITIILSRRRR RR-COOH        (22 AA)
```

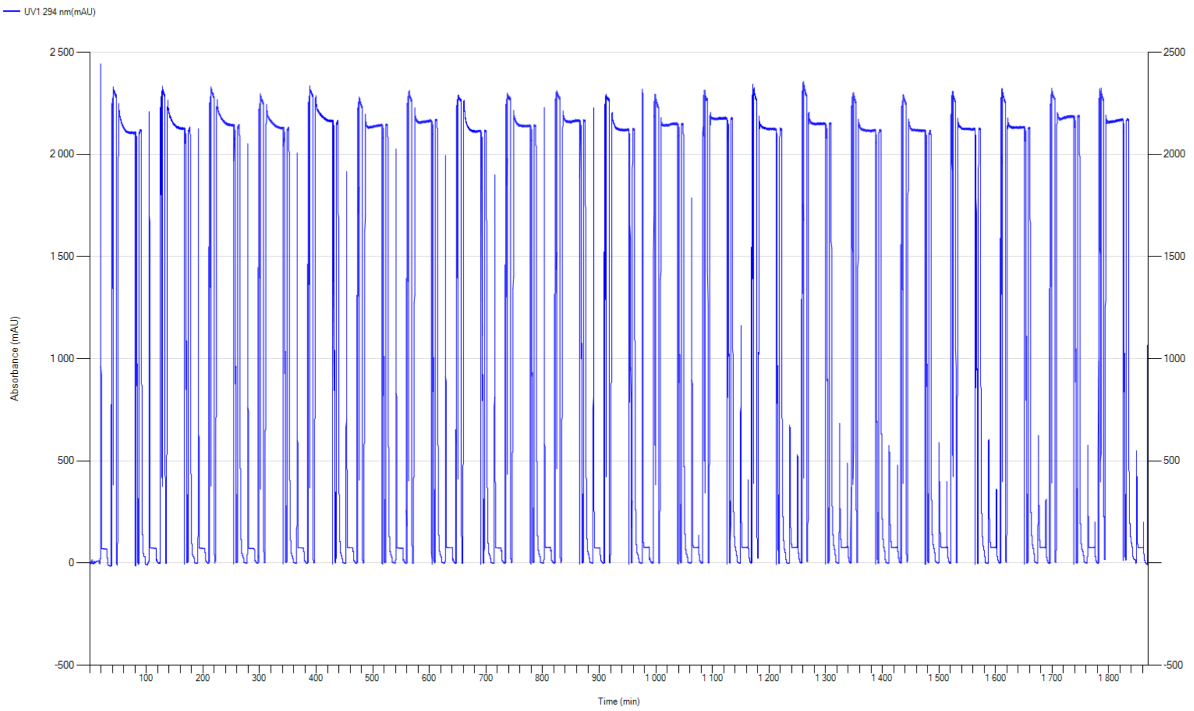

SI Figure 110. Run curve from PeptiPilot at 294 nm. Synthesis of Barstar[75–90]-ArgTag.

The peptide Barstar[75–90] was synthesized on commercially available 2-CTC polystyrene resin onto which the Fmoc-Arg(Pbf)-OH was manually pre-loaded. To pre-load, the resin (1.2 g) was washed with DMF (3 × 5 mL) and then swollen for 10 min. Neat DIPEA (1 mL) was added to the stock solution of Fmoc-Arg(Pbf)-OH (0.4 M, 7 mL) in DMF, stirred for 1 min and then transferred to the swollen resin. The mixture was gently agitated for 1 h, then the resin was filtered off and washed with DMF (3 × 5 mL). This step was then repeated once before the resin was finally washed with DMF (3 × 5 mL), then DCM (3 × 5 mL). The resin dried under a flow of air and then under vacuum. The loading of the resin was determined according to **Section 2.5** to be 0.69 mmol/g. The synthesis of resin-bound Barstar[75–90]-ArgTag was performed according to **Section 2.2.3** and total synthesis time was approximately 26 h (**SI Figure 110**). Cleavage of the peptidyl-resin (74 mg, approx. 67 μmol) according to the cleavage protocol described in **Section 2.6** afforded the crude peptide as a colorless solid (33.5 mg, mass confirmed by LC-HR-ESI-QTOF [**SI Figure 111**], 50% purity by UHPLC [**SI Figure 112**]).

LC-QTOF of crude Barstar[75–90]-ArgTag

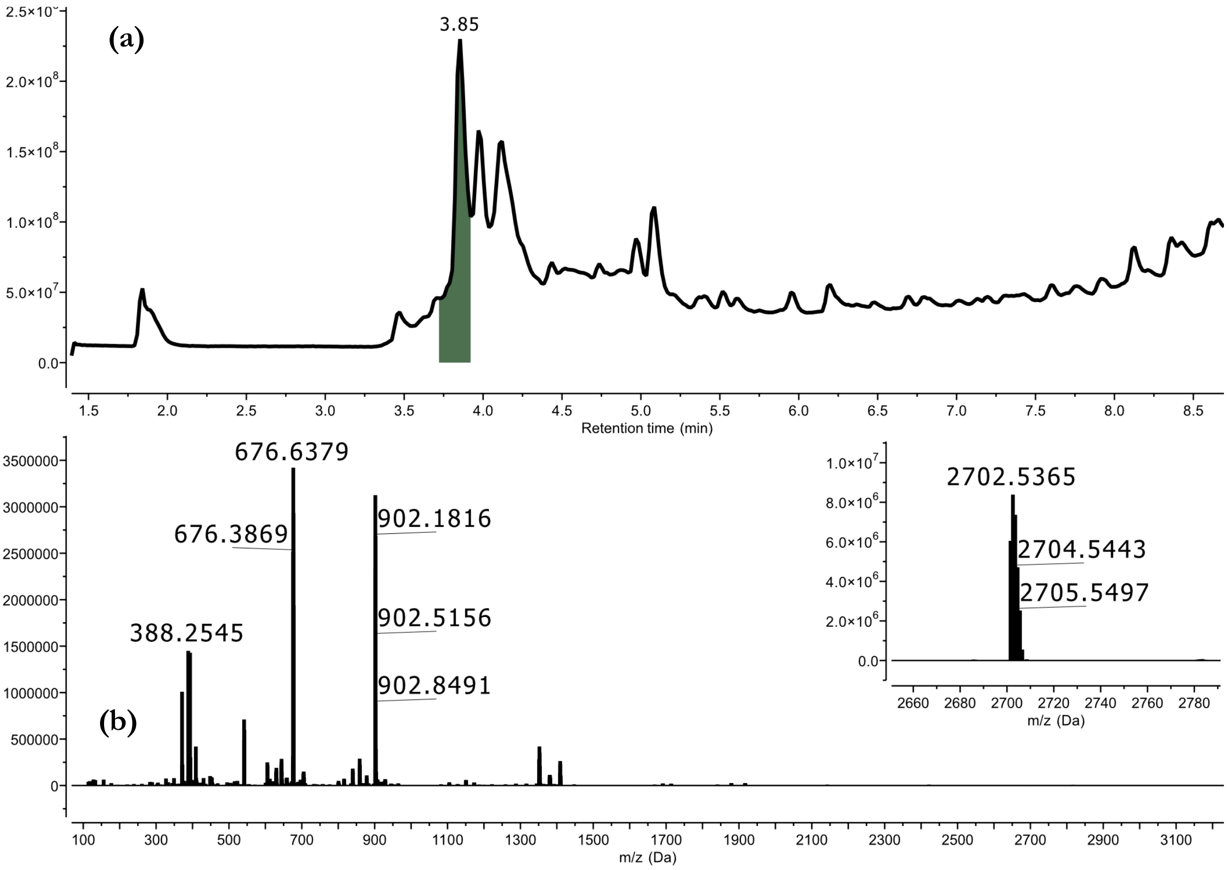

SI Figure 111. LC-HR-ESI-QTOF Profile of crude Barstar[75–90] bearing the ArgTag. (a) TIC chromatogram of Barstar[75–90]-ArgTag; Rt 3.85 min. (b) ESI-TOF spectrum found within Rt 3.85 min (insert: deconvoluted masses). Monoisotopic mass (ESI+) calcd. for $C_{113}H_{200}N_{44}O_{31}S$ 2701.5147, found 2701.5307. LCMS Gradient A (**Section 2.8**).

UHPLC of crude Barstar[75–90]-ArgTag

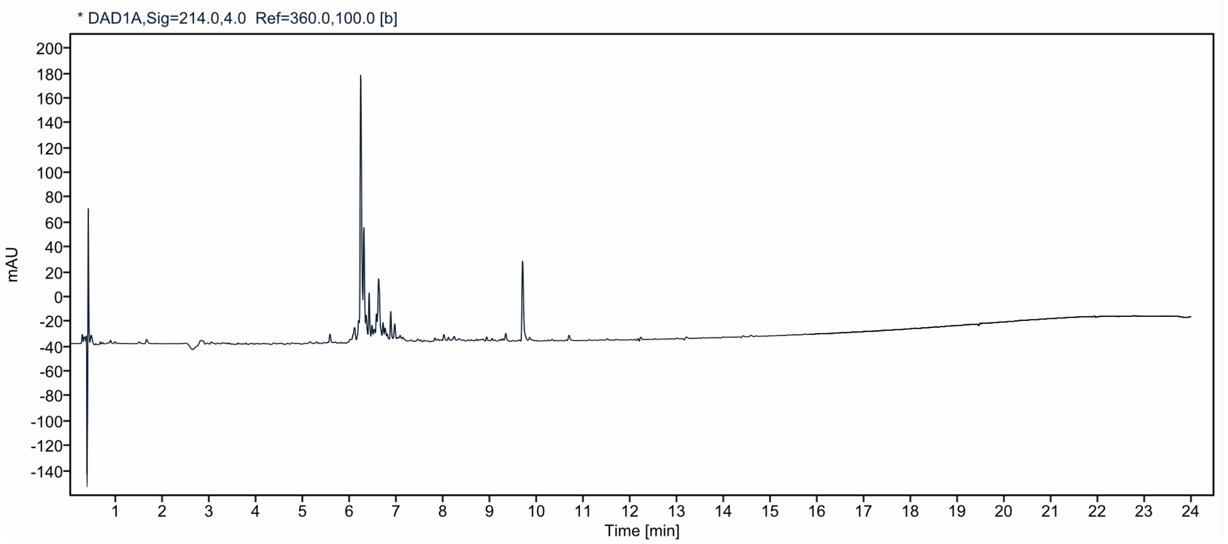

SI Figure 112. UHPLC profile of crude Barstar[75–90] bearing ArgTag. Rt 6.22 min (Agilent Zorbax 300SB-C18 RRHD column, 1.8 µm, 2.1 × 50 mm, 5–95% MeCN over 20 min, ca. 4.5%B/min), 50% purity based on Area Under Curve (AUC) at λ = 214 nm.

6 References

(1) Hartrampf, N.; Saebi, A.; Poskus, M.; Gates, Z. P.; Callahan, A. J.; Cowfer, A. E.; Hanna, S.; Antilla, S.; Schissel, C. K.; Quartararo, A. J.; Ye, X.; Mijalis, A. J.; Simon, M. D.; Loas, A.; Liu, S.; Jessen, C.; Nielsen, T. E.; Pentelute, B. L. Synthesis of Proteins by Automated Flow Chemistry. *Science,* **2020**, *368*, 980–987. https://doi.org/10.1126/science.abb2491.

(2) Prosser, L. C.; Talbott, J. M.; Garrity, R. P.; Raj, M. C-Terminal Arginine-Selective Cleavage of Peptides as a Method for Mimicking Carboxypeptidase B. *Org Lett* **2023**, *25* (33), 6206–6210. https://doi.org/10.1021/acs.orglett.3c02418.

(3) Folk, J. E. Carboxypeptidase B (Porcine Pancreas). *Methods Enzymol* **1970**, 504–508.

(4) Bürgisser, H.; Williams, E. T.; Jeandin, A.; Lescure, R.; Premanand, A.; Wang, S.; Hartrampf, N. A Versatile "Synthesis Tag" (SynTag) for the Chemical Synthesis of Aggregating Peptides and Proteins. *J. Am. Chem. Soc.* **2024**. https://doi.org/10.1021/jacs.4c14247.
